# Synthesis of the Tetrasaccharide Repeating Unit from *Klebsiella pneumoniae* Serotype K2 Capsular Polysaccharide

**DOI:** 10.1021/acsomega.5c03675

**Published:** 2025-06-23

**Authors:** Mohammad Tarique Anwar, Todd L. Lowary

**Affiliations:** † 71561 Institute of Biological Chemistry, Academia Sinica, Academia Road Section 2, #128, Nangang, Taipei 115, Taiwan; ‡ Institute of Biochemical Sciences, National Taiwan University, Taipei, 106, Taiwan

## Abstract

Described is the synthesis of the two tetrasaccharides (**1** and **2**) related to the repeating unit from the *Klebsiella pneumoniae* Serotype K2 capsular polysaccharide. The compounds differ by the presence or absence of an acetate ester on O-2 of the mannopyranose residue of the repeating unit. Κey challenges in the synthesis were the installation of three 1,2-*cis* glycosidic linkages, including a β-mannopyranoside, the selective introduction of an α-d-glucuronic acid residue, and the late-stage incorporation of an acetate ester in **2**. A convergent approach employing a key [2 + 2] glycosylation was initially explored, but the key C-2 inversion needed to install the β-mannopyranoside in the resulting tetrasaccharide failed. An alternative strategy, using a sequential glycosylation approach, was successful in providing both targets. The developed route can be adapted to provide analogs containing other modifications. Such compounds are useful probes for immunological and structural biology investigations.

## Graphical abstract

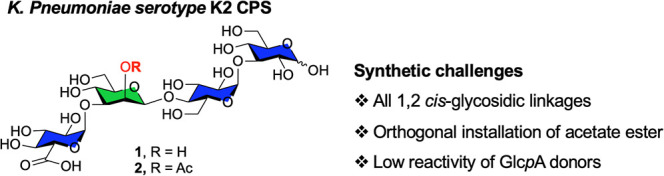

## Introduction

*Klebsiella pneumoniae* is a Gram-negative bacterium that causes life-threatening infections such as pneumonia, sepsis and urinary tract infections primarily among newborns and critically ill patients.^1,2^ The organism belongs to a group of highly virulent and multidrug resistant bacteria called the “ESKAPE” pathogens.^3^ In 2017,^4^ the World Health Organization designated these organisms as a public health priority in the fight against antibiotic resistance. The *K. pneumoniae* outer membrane is coated with carbohydrate structures including capsular polysaccharides (CPS, K Antigens) and lipopolysaccharide, which act as a defense against host immune responses and antimicrobial agents. These polysaccharides are also ligands for bacteriophage receptor binding proteins and substrates for phage depolymerases.^5,6^

To date, a total of 79 *K. pneumoniae* serotypes based on the CPS have been identified.^2^ Among the most prevalent is serotype K2, which is associated with community acquired pneumonia, sepsis and other invasive infections.^7–9^ The structure of the (K2 CPS is →4)-[α-d-Glc*p*A-(1→3)]-β-d-Man*p*-(1→4)-α-d-Glc*p*-(1→3)-β-d-Glc*p-*(1→,^10^ and the structure can be modified by nonstoichiometric acetylation).^11^ As part of a larger study focusing on the characterization of *K. pneumoniae* bacteriophage CPS depolymerases,^9,11,12^ we became interested in accessing both the K2 parent tetrasaccharide repeating unit and a derivative in which the Man*p* residue was acetylated on the C-2 hydroxyl group. In particular, preliminary immunological data suggested a difference in the ability of acetylated and deacylated forms of the products liberated by bacteriophage CPS depolymerases to elicit proinflammatory cytokines, with the position of the acetylation being important (Dr. Shih-Hsiung Wu, personal communication). We envisioned these compounds would be useful ligands in X-ray crystallographic studies and as substrates for immunological investigations. More generally, structurally defined synthetic oligosaccharide fragments are valuable biological probes for immunological investigations.^13,14^

Previous reports have described syntheses of the K2 CPS repeating unit as either the methyl^15^ or aminopentyl glycoside.^16^ Our goal was to access to the reducing sugars liberated from the CPS by a phage depolymerase.^11^ This enzyme cleaves the polysaccharide between the β-d-Glc*p* and β-d-Man*p* unit, giving a linear tetrasaccharide: α-d-Glc*p*A-(1→3)-β-d-Man*p*-(1→4)-α-d-Glc*p*-(1→3)-β-d-Glc*p*, as well as a dimer of this motif. Thus, the structures produced by these earlier routes were not of utility, given the presence of an aglycone and their frame-shifted structures, which differ from that produced by the enzyme. Instead, two targets—**1** and **2** (Scheme 1)—were chosen for synthesis.

**Scheme 1:**
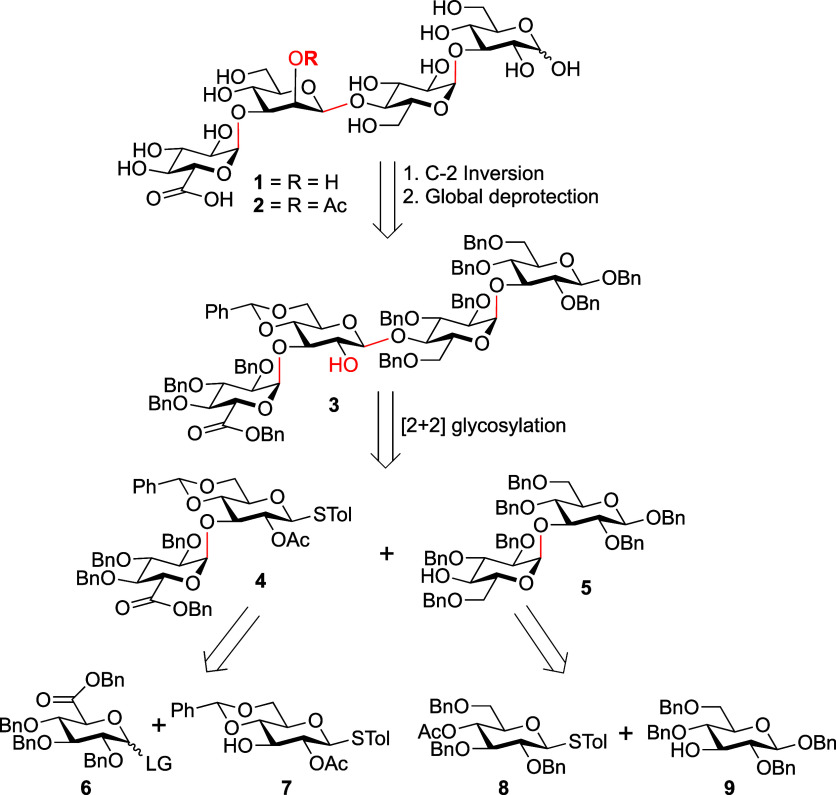
Retrosynthesis of Tetrasaccharides **1** and **2**

## Results and Discussion

### Retrosynthesis

The key challenges in the synthesis of **1** and **2** are (1) the stereoselective installation of three different 1,2-*cis*-glycosidic bonds—α-d-Glc*p*A-(1→3)-d-Man*p*, α-d-Glc*p*-(1→3)-d-Glc*p*, and β-d-Man*p*-(1→4)-d-Glc*p*, the latter of which has historically been particularly difficult to install with good stereocontrol; (2) developing a strategy for introducing the α-d-Glc*p*A residue (either directly or by oxidation of an α-d-Glc*p* residue postglycosylation); and (3) implementing a protecting group strategy for introducing and maintaining the acetate ester present in **2**. With regard to the first two challenges, previous syntheses of the frame-shifted derivatives of **1** employed either Crich β-mannosylation,^17^ or a redox strategy,^18^ to introduce the β-d-Man*p* residue. With regard to the second challenge, both earlier-reported routes installed the Glc*p*A residue by a late-stage O-6 oxidation of an α-d-Glc*p* residue in the oligosaccharide.

In designing our own route, we chose to employ an inversion strategy to introduce the β-d-Man*p* residue, which also allows the introduction of the base-labile acetate ester in **2** late in the synthetic route. We saw this as advantageous over the use of the Crich β-mannosylation,^17^ which would have required the use of a donor with an orthogonal nonparticipating group that would have needed to be removed and before acylation of O-2 of the β-d-Man*p* residue. Thus, as illustrated in Scheme 1, C-2 oxidation–reduction or C-2 inversion by triflation displacement^19^ of tetrasaccharide **3** followed by Pd-catalyzed hydrogenation would afford **1**; alternatively, acetylation of the inverted product and hydrogenation would produce **2**. The synthesis of tetrasaccharide **3** was to be achieved by a convergent [2 + 2] glycosylation strategy by coupling of **4** and **5**, each containing a 1,2-*cis*-glycosidic linkage. Notably, this strategy allowed this key glycosylation to be carried out using neighboring group participation ensuring good stereocontrol. To synthesize disaccharides **4** and **5**, the 1,2-*cis*-linkages were to be constructed by adopting donors C-2 nonparticipating groups and solvent effects.^20^ In contrast to the earlier synthetic work related to these structures, we decided to introduce the uronic acid residue from a suitably protected Glc*p*A donor (**6**), protected as a benzyl ester, which can be removed together with other benzyl ethers (Bn) in the final hydrogenation step.

### Synthesis of Disaccharide Building Blocks 4 and 5

The synthesis commenced with the assembly of disaccharide **5** (Scheme 2), which would serve as the acceptor in the [2 + 2] glycosylation. Commercially available diacetone-d-glucose was used as starting material and the C-3 hydroxyl group was first protected as an allyl ether, followed by acid hydrolysis of di-*O*-isopropylidine ketals and subsequent per-acetylation of the resulting reducing sugar to yield **10** in 85% yield over the three steps. Conversion of **10** into the corresponding trichloroacetimidate donor **11**, and glycosylation with benzyl alcohol in the presence of TMSOTf as the promoter, afforded **12** with β-selectivity (^3^ *J*_H‑1,H‑2_ = 8.0 Hz). The ester groups were removed by Zemplen deacetylation, followed by *O*-alkylation with benzyl bromide. Successive removal of the allyl ether via a two-step process involving an initial Ir-catalyzed^21^ isomerization followed by oxymercuration–demercuration with HgO and HgCl_2_ yielded monosaccharide acceptor **9** in excellent yield (79% over four steps).

**Scheme 2:**
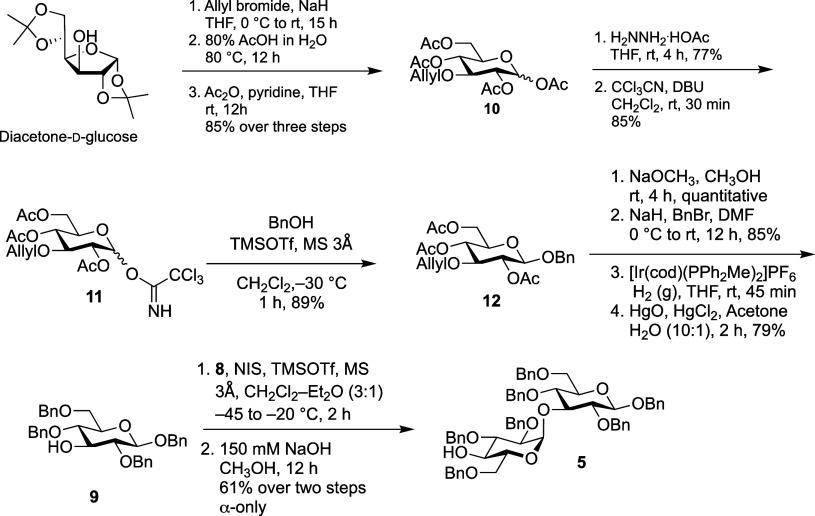
Synthesis of Acceptor **5**

To stereoselectively install the α-d-Glc*p*-(1→3)-d-Glc*p* glycosidic linkage, the previously reported thioglycoside donor **8** ^22^ and monosaccharide acceptor **9** were coupled together in the presence of NIS/TMSOTf as the activator. We initially explored adding *N*,*N*-dimethylformamide to enhance the α-selectivity of the glycosylation,^23^ but were unsuccessful due to slow activation of donor (up to 24 h) leading to hydrolysis after addition of acceptor. A shift was then made to rely on the α-directing effect of diethyl ether.^24^ An initial attempt in a 1:1 mixture of dichloromethane and diethyl ether resulted in a very slow reaction that failed to complete. However, in a 3:1 mixture of dichloromethane and diethyl ether, the reaction proceed efficiently (complete consumption of **9** was observed on TLC) to give a 3.5:1 α/β mixture of products. Unfortunately, due to their identical chromatographic mobilities, it was not possible to separate the mixture of glycoside products that were formed. However, after removal of the acetate ester using sodium hydroxide in methanol, the desired compound **5** was obtained in 61% yield (α only). Confirmation of the stereochemistry in the product of the glycosylation reaction was determined using ^1^H and ^13^C NMR spectroscopy. The ^3^ *J*_H‑1,H‑2_ of the newly introduced residue in the ^1^H NMR spectrum was 3.5 Hz and the chemical shift of the C-1 of this residue in the ^13^C NMR spectrum was 97.2 ppm; both data are consistent with the α-stereochemistry.^25^ It should be noted that deprotection of the acetate ester leading to **5** was sluggish. Under Zemplen deacetylation conditions using sodium methoxide concentrations ranging from catalytic to stoichiometric, or by prolonging the reaction time (48 h), the reaction failed to complete. We attribute this to steric hindrance by the benzyl groups surrounding the C-4 position in the α-linked Glc*p* residue, which appears to slow down the nucleophilic attack of methoxide onto the ester carbonyl and subsequent formation of the corresponding tetrahedral intermediate.

Next, we set out to synthesize disaccharide donor **4** (Scheme 3). Construction of glucuronic acid linkages are challenging due to the presence of the electron withdrawing C-5 carboxylic acid group, which reduces the donor reactivity.^26^ Therefore, activating benzyl protecting groups were chosen to improve the reactivity and, by virtue of their nonparticipating character, the α-stereoselectivity. Two different glucuronic acid donors—thioglycoside **14** and trichloroacetimidate **15**—were prepared starting from thioglycoside **13**, which was synthesized as previously described.^27^ First, regioselective reductive 4,6-*O*-benzylidine acetal cleavage with BH_3_·THF and TMSOTf yielded the isomer with a C-6 hydroxyl group, which was then oxidized using catalytic 2,2,6,6-tetramethyl-1-piperidinyloxyl (TEMPO) and excess [bis­(acetoxy)-iodo]­benzene (BAIB). Esterification of the carboxylic acid product upon treatment with benzyl bromide and sodium bicarbonate furnished **14** in 76% overall yield from **13**. Subsequent oxidative hydrolysis of thioglycoside with NIS in wet acetone followed by conversion of the lactol intermediate to the corresponding trichloroacetimidate donor with trichloroacetonitrile in the presence of 1,8-diazabicyclo[5.4.0]­undec-7-ene (DBU) provided **15** in 90% yield over the two steps. Donor **15** was then used to glycosylate the known acceptor **7** ^28^ using TMSOTf as the activator in a 3:1 mixture of dichloromethane and diethyl ether. This reaction provided **4** in 74% yield and with high α-selectivity. The stereochemistry of the Glc*p*A–Glc*p* linkage in **4** was established on the basis of ^1^H and ^13^C NMR spectroscopic data (^3^ *J*_H‑1/H‑2_ = 3.4 Hz and a C-1 chemical shift of 96.4 ppm).

**Scheme 3:**
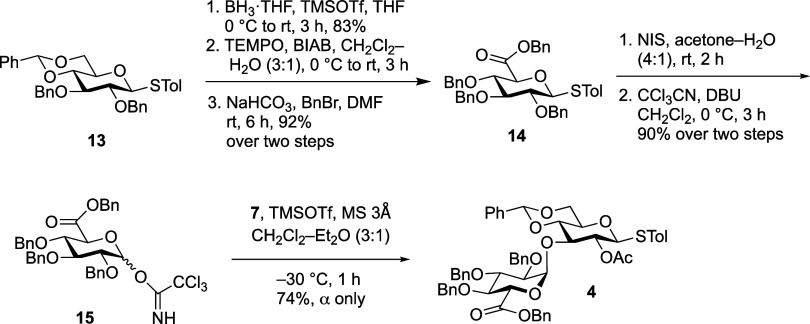
Synthesis of Disaccharide Donor **4**

**Scheme 4:**
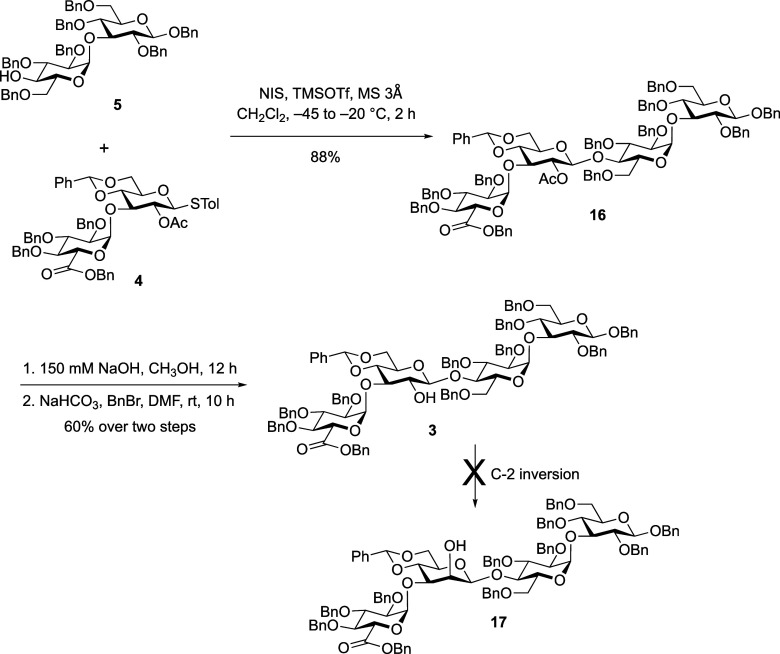
Attempted Synthesis of Tetrasaccharide **17**

### Synthesis of Tetrasaccharide 3

Having success in preparing the intermediate building blocks, the coupling of disaccharide donor **4** and acceptor **5** was explored (Scheme 4). The reaction, performed in the presence of NIS/TMSOTf as promoters in dichloromethane, proceeded efficiently providing exclusively the β-linked tetrasaccharide **16** (^3^ *J*_H‑1/H‑2_ = 8.0 Hz) in high yield (88%). We note that although the sluggishness in deprotecting the acetate ester in the precursor to **5** suggested this portion of the molecule was sterically hindered, the glycosylation was nevertheless not problematic. We hypothesize that the O-3 and O-6 benzyl protecting groups in the α-Glc*p* residue of compound **5** and the O-4 acetylated precursor adopt different orientations, with the latter experiencing greater congestion around the O-4 position than the former. However, this remains only a hypothesis, and we have not further investigated the reactivity differences between **5** and its O-4 acetylated derivative.

To carry out the inversion at C-2 in the benzylidene-protected Glc*p* residue in **3**, it was necessary to selectively remove the acetate ester on the β-linked Glc*p* moiety. This proved difficult as both the benzyl and acetate esters were removed simultaneously via saponification with sodium hydroxide in methanol. Thus, reinstallation of the benzyl ester was necessary and this was achieved using benzyl bromide in the presence of sodium bicarbonate providing **3** in 60% yield over the two steps. Unfortunately, all of our attempts to invert C-2 on the β-linked Glc*p* residue in **3**, either by triflation and then nucleophilic substitution or oxidation/reduction, failed (see Table 1). In all cases the starting alcohol was recovered during attempted triflation or oxidation. A redox approach had previously been employed^15^ in the synthesis of a frame-shifted derivative of this tetrasaccharide, but, in that case, the Glc*p* residue undergoing inversion was at the reducing end of the molecule and the aglycone was a methyl group. It appears that this position in **3** is sterically crowded due to the presence of adjacent α-Glc*p*A-(1→3) linkage and the carbohydrate aglycone.

**Table 1:** Conditions Attempted for C-2 Inversion of the Glc*p* Residue in **3**

entry	conditions	outcome
1	Tf_2_O, pyridine, 0 °C rt	starting material recovered
2	Tf_2_O, pyridine, DMAP 0 °C rt	starting material recovered
3	Dess–Martin periodinane, CH_2_Cl_2_, rt	starting material recovered
4	(1) oxalyl chloride, DMSO, –78 °C; (2) DIPEA	starting material recovered

### Linear Glycosylation Strategy

After failing to access the target via the [2 + 2] approach outlined above, we investigated a linear glycosylation approach as shown in Scheme 5. Our aim was to reduce the steric congestion around C-2 on the β-linked Glc*p* residue in the inversion step by using a trisaccharide substrate lacking the terminal α-linked Glc*p*A. Thus, disaccharide **5** was coupled with trichloroacetimidate donor **11** in the presence of TMSOTf as the promoter to yield the corresponding trisaccharide in 80% yield. The acetate groups were removed with sodium hydroxide, followed by regioselective installation of a 4,6-*O*-benzylidene acetal generating **18** in 87% yield. The anomeric stereochemistry of the terminal Glc*p* residue in **18** was established by measuring ^1^H and ^13^C NMR spectroscopy data (^3^ *J*_H‑1/H‑2_ = 7.4 Hz and a C-1 chemical shift of 103.7 ppm).

**Scheme 5:**
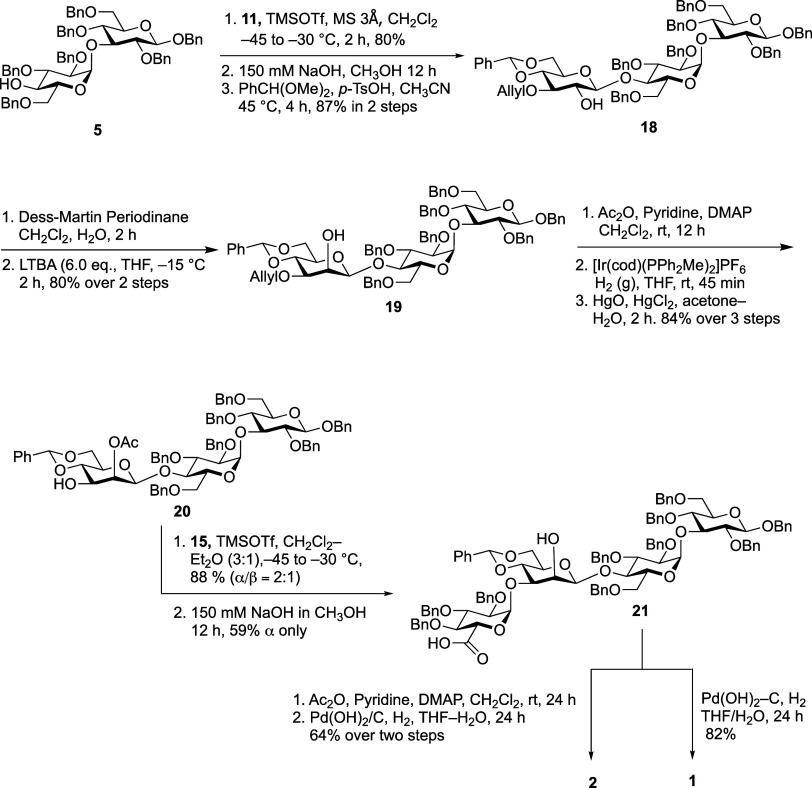
Sequential Glycosylation Strategy Leading to **1** and **2**

The C-2 hydroxy group of the terminal Glc*p* residue in **18** was oxidized with Dess–Martin periodinane to obtain a ketone intermediate. The hassle-free oxidation of **18** supports our hypothesis the that presence of the α-(1→3)-linked Glc*p*A residue in **3** prevented the successful oxidation in the tetrasaccharide. A subsequent highly β-*manno*-selective reduction of the ketone using a bulky reducing agent—lithium tritert-butoxyaluminum hydride (LTBA)—furnished **19** in 80% yield over the two-step redox transformation. Although 16% of the retention product (**3**) was also isolated, these results were considerably better than obtaining equimolar ratio of epimers with sodium borohydride (NaBH_4_) in comparable systems.^29^ The stereoselectivity of the reduction was determined from the ^1^ *J*_C1–H1_ (∼158 Hz) and *J*_H‑1/H‑2_ (7.4 Hz for β-*gluco*, changing to <1.0 Hz for β-*manno*). Protection of the C-2 hydroxyl group as an acetate ester and removal of the allyl ether via a two-step process furnished trisaccharide acceptor **20** in 84% yield over the three steps.

With a route developed to access trisaccharide **20**, all that remained to obtain **1** and **2** was the introduction of the α-linked Glc*p*A residue and deprotection. Initial attempts to couple **20** and thioglycoside **14** in the presence of NIS/TMSOTf failed to give the expected tetrasaccharide; instead, only donor decomposition and recovery of the acceptor was observed. The failure of **14** and **20** to engage in glycosylation may be the result of the reactivity of the Glc*p*A thioglycoside donor. Consequently, a trichloroacetimidate donor **15** was employed. Glycosylation of **20** in 3:1 dichloromethane–diethyl ether using TMSOTf as the promoter provided the tetrasaccharide in 88% yield, albeit with modest 1,2-*cis*-selectivity (α/β = 2:1). Unfortunately, the two diastereomers could not be separated from each other, or from the hydrolyzed donor. However, after the removal of ester groups with sodium hydroxide in methanol, we could separate the mixture and the desired compound **21** was isolated in 59% yield (α only). The stereochemistry of the Glc*p*A–Man*p* linkage was established on the basis of ^1^H and ^13^C NMR spectroscopy data (^3^ *J*_H‑1/H‑2_ = 3.6 Hz and a C-1 chemical shift of 97.3 ppm). One portion of tetrasaccharide **21** was treated with palladium hydroxide on charcoal in the presence of H_2(g)_ leading to **1** in good isolated yield (82%). To prepare the acetate derivative **2**, the other portion was treated with acetic anhydride in the presence of DMAP to acylate the C-2 hydroxyl group of the Man*p* residue. Hydrogenolysis of the acetylated compound gave **2** in 64% yield over two steps.

## Conclusion

We report a synthetic approach to the *K. pneumoniae* serotype K2 CPS tetrasaccharide repeating unit with/without the Man*p* residue acetylated at O-2. Two routes were explored, one involving a [2 + 2] glycosylation strategy and another employing a linear ([1 + 1 + 1 + 1]) approach. While both routes could be implemented to get a tetrasaccharide product, the compound obtained from the [2 + 2] strategy did not undergo a subsequent key inversion reaction. As such, the linear approach is preferred. All glycosylations proceeded in good yield and with acceptable stereoselectivity. Solvent participation was used to construct the 1,2 cis-Glc*p* and Glc*p*A linkages and the β-Man*p* linkage was installed indirectly by C-2 inversion of a β-Glc*p* residue. Thoughtful selection of orthogonal protecting groups on the β-Man*p* residue provide an opportunity for late step modification to access compounds modified on the C-2 hydroxyl group, in this case an acetate ester. Immunological investigations with **1** and **2**, and their use in structural biology investigations, are ongoing to elucidate the role of aceylation on pro-imflammatory cytokine stimulation.

## Experimental Section

### General Methods

All reagents were purchased from commercial sources and used without further purification unless otherwise noted. Solvents used in reactions were purified by passage through alumina and copper columns under argon. Unless otherwise noted, reactions were carried out at room temperature (rt), under argon, and were monitored by TLC on silica gel G-25F 254 (0.25 mm). TLC spots were detected under UV light and charring after staining with *p*-anisaldehyde in ethanol, acetic acid and H_2_SO_4_. All concentration/evaporation steps were carried out under reduced pressure on a rotary evaporator. Column chromatography was performed on silica gel 60 (40–60 μM) unless otherwise noted. ^1^H NMR spectra were recorded at 500 or 600 MHz and referenced to the solvent residual proton signal of CDCl_3_ (7.26 ppm) or D_2_O (4.79 ppm). ^13^C NMR spectra were recorded at 125 or 150 MHz and referenced to CDCl_3_ (77.06 ppm), CD_3_OD (3.31 ppm) or external acetone (31.07 ppm, D_2_O). Peak assignments were made based on 2D NMR analysis (^1^H–^1^HCOSY, HSQC, HMBC). In the case of oligosaccharides, ^1^H and ^13^C NMR assignments of each glucose residue are labeled as Glc_a_, Glc_b_ and Glc_c_ proceeding from the reducing end to the nonreducing end. For other sugar residues, mannose is labeled as “Man” and glucuronic acid as “GlcA”. For regions of the ^13^C spectra with significant signal overlap, the number of carbons contributing to each resonance was not identified. High-resolution ESI mass spectra were recorded on an Agilent Technologies 6220 spectrometer or a JMS-T100LP AccuTOF LC-plus 4G TOF mass spectrometer (JEOL, Tokyo, Japan) using samples in CH_3_OH and added NaCl. Spectra were obtained by voltage scan over a narrow range at a resolution of approximately 10,000.

#### α-d-Glucopyranosyluronate-(1→3)-β-d-mannopyranosyl-(1→4)-α-d-glucopyranosyl-(1→3)-d-glucopyranoside **(1)**

To a solution of compound **21** (30 mg, 0.0179 mmol) in THF–H_2_O (6.0 mL, 1:1) was added Pd­(OH)_2_–C (10.0 mg). The resulting solution was stirred under H_2(g)_ atmosphere for 24 h. Then, the catalyst was filtered through a pad of Celite and the filtrate was concentrated. The residue was purified by C-18 gel chromatography using 0 to 15% CH_3_OH in H_2_O as eluent to afford **1** (10 mg, 0.014 mmol, 82% yield) as a white foam. *R*_f_ = 0.15, EtOAc–CH_3_OH–H_2_O–AcOH = 5:2:1:0.5; ^1^H NMR (600 MHz, CDCl_3_, δ_H_): 5.38 (d, *J* = 3.9 Hz, 1H, H-1 GlcA), 5.36 (d, *J* = 3.9 Hz, 1H, H-1 GlcA), 5.30 (d, *J* = 3.9 Hz, 1H, H-1 Glc_b_), 5.23 (d, *J* = 3.8 Hz, 1H, H-1 α Glc_a_), 4.79 (s, 1H, H-1 Man), 4.66 (d, *J* = 8.0 Hz, 1H, H-1 β Glc_a_), 4.35 (d, *J* = 10.0 Hz, 1H, H-5 GlcA), 4.18 (d, *J* = 1.4 Hz, 1H, H-2 Man), 4.11–4.09 (m, 1H, H-3 Man), 3.95 (dd, *J* = 2.1, 12.4 Hz, 1H), 3.92–3.81 (m, 5H), 3.79–3.69 (m, 7H), 3.67–3.59 (m, 5H), 3.48–3.46 (m, 1H), 3.35–3.31 (m, 1H, H-2 β Glc_a_). ^13^C NMR (150 MHz, CDCl_3_, δ_C_): 173.2 (C=O), 100.7 (C-1, Glc_b_), 99.8 (C-1 Man), 98.7 (C-1, GlcA), 95.9 (C-1, β Glc_a_), 92.1 (C-1, α Glc_a_), 82.1, 81.3, 79.4, 78.7, 78.5, 76.0, 75.6, 72.7, 72.3, 71.4, 71.3, 71.2, 71.1, 70.5, 70.2, 70.0, 65.7, 60.8, 60.5, 60.3, 59.8, 59.7. HRMS (ESI) calcd for [M – H]^−^ C_24_H_39_O_22_: 679.1939; found, 679.1941.

#### α-d-Glucopyranosyluronate-(1→3)-2-*O*-acetyl-β-d-mannopyranosyl-(1→4)-α-d-glucopyranosyl-(1→3)-d-glucopyranoside (**2**)

To a solution of compound **21** (35 mg, 0.029 mmol) dissolved in pyridine (2.0 mL), cooled to 0 °C, acetic anhydride (9.0 μL, 0.087 mmol) was added followed by the addition of DMAP (1.6 mg, 0.007 mmol). The reaction mixture was gradually warmed to rt and stirred for 12 h, before being concentrated. The residue was diluted with EtOAc (25 mL) and the resulting organic solution was sequentially washed with 1 N HCl_(aq)_, saturated NaHCO_3(aq),_ H_2_O and brine. The organic layer was dried over MgSO_4_, filtered and concentrated before being moved to the next step without purification. To a solution of the crude residue in THF–H_2_O (6.0 mL, 1:1) was added Pd­(OH)_2_–C (10.0 mg). The resulting solution was stirred under a H_2(g)_ atmosphere for 24 h at rt. The catalyst was then filtered through a pad of Celite and the filtrate was concentrated. The resulting residue was purified by C-18 gel chromatography using 0 to 20% CH_3_OH in H_2_O as eluent to afford **2** (13 mg, 0.018 mmol, 64% yield) as a white foam. *R*_f_ = 0.20, EtOAc–CH_3_OH–H_2_O–AcOH = 5:2:1:0.5; ^1^H NMR (600 MHz, CDCl_3_, δ_H_): 5.56 (d, *J* = 3.3 Hz, 1H, H-2 Man), 5.35 (d, *J* = 3.9 Hz, 1H, H-1 GlcA), 5.33 (d, *J* = 3.9 Hz, 1H, H-1 GlcA), 5.26 (d, *J* = 3.5 Hz, 1H, H-1 Glc_b_), 5.23 (d, *J* = 3.8 Hz, 1H, H-1 α Glc_a_), 4.99 (s, 1H, H-1 Man), 4.65 (d, *J* = 8.0 Hz, 1H, H-1 β Glc_a_), 4.04–3.96 (m, 4H, H-3 Man, H-5 GlcA), 3.89 (dd, *J* = 2.2, 12 Hz, 1H), 3.86–3.81­(m, 3H), 3.78–3.75 (m, 3H), 3.73–3.68 (m, 3H), 3.65–3.53 (m, 5H), 3.49–3.46 (m, 1H), 3.33–3.30 (m, 1H), 2.21 (s, 3H, CH_3_CO). ^13^C NMR (150 MHz, CDCl_3_, δ_C_): 176.5 (CO), 173.0 (C=O), 100.0 (C-1, Glc_b_), 99.0 (C-1 Man), 98.8 (C-1, GlcA), 95.9 (C-1, β Glc_a_), 92.1 (C-1, α Glc_a_), 82.1, 79.7, 79.5, 79.4, 77.4, 76.2, 75.6, 72.7, 72.4, 72.1, 71.8, 71.7, 71.6, 71.3, 71.2, 71.1, 70.0, 66.8, 60.7, 60.5, 60.3, 59.6, 59.5, 20.2. HRMS (ESI) calcd for [M – H]^−^ C_26_H_41_O_23_: 721.2044; found, 721.2035.

#### Benzyl-3,4,6-tri-*O*-benzyl-α-d-glucopyranosyluronate-(1→3)-4,6-*O*-benzylidene-β-d-glucopyranosyl-(1→4)-2,3,6-tri-*O*-benzyl-α-d-glucopyranosyl-(1→3)-1,2,4,6-tetra-*O*-benzyl-β-d-glucopyranoside (**3**)

To a solution of tetrasaccharide **16** (40.0 mg, 0.022) in CH_3_OH (4.2 mL), was added 1 M NaOH (750 μL) and the mixture was stirred at rt. After 12 h of stirring, the reaction mixture was concentrated and the residue was dissolved in EtOAc (30.0 mL) and washed with H_2_O and brine. The organic layer was separated, dried over MgSO_4_, filtered and concentrated. The crude residue obtained was dried on the vacuum for 2 h and then dissolved in DMF (4.0 mL). Next, NaHCO_3_ (7.4 mg, 0.088 mmol) and BnBr (8.0 μL, 0.066 mmol) were added into the solution and the mixture was stirred for 10 h before being concentrated. The residue was diluted with EtOAc (30 mL) and the resulting organic solution was washed with H_2_O and brine. The organic layer was separated, dried over MgSO_4_, filtered and concentrated. The crude residue was purified by chromatography (EtOAc–Hexanes = 1:2) to afford **3** (23 mg, 0.013 mmol, 60% over two steps) as a white solid. *R*_f_ = 0.10, EtOAc–Hexanes = 1:2; ^1^H NMR (600 MHz, CDCl_3_, δ_H_): 7.35–7.26 (m, 8H, ArH), 7.31–7.26 (m, 18H, ArH), 7.24–7.22 (m, 16H, ArH), 7.20–7.14 (m, 8H, ArH), 7.10–7.08 (m, 6H, ArH), 7.06–7.04 (m, 2H, ArH), 6.91–6.90 (m, 2H, ArH), 5.56 (d, *J* = 3.5 Hz, 1H, H-1 GlcA), 5.47 (d, *J* = 3.5 Hz, 1H, H-1 Glc_b_), 5.30 (s, 1H, PhCH), 5.21 (d, *J* = 12.2 Hz, 1H, OCHPh, 5.11 (d, *J* = 12.2 Hz, 1H, OCHPh), 5.00 (d, *J* = 10.6 Hz, 1H, OCHPh), 4.98 (d, *J* = 10.6 Hz, 1H, OCHPh), 4.94 (d, *J* = 12.2 Hz, 1H, OCHPh), 4.92 (d, *J* = 10.2 Hz, 1H, OCHPh), 4.91 (d, *J* = 11.0 Hz, 1H, OCHPh), 4.87 (d, *J* = 11.5 Hz, 1H, OCHPh), 4.81 (d, *J* = 11.0 Hz, 1H, OCHPh), 4.78 (d, *J* = 11.0 Hz, 1H, OCHPh), 4.77 (d, *J* = 11.0 Hz, 1H, OCHPh), 4.72 (d, *J* = 11.5 Hz, 1H, OCHPh), 4.68 (d, *J* = 11.8 Hz, 1H, OCHPh), 4.67 (d, *J* = 12.0 Hz, 1H, OCHPh), 4.63 (d, *J* = 12.2 Hz, 1H, OCHPh), 4.55 (d, *J* = 11.6 Hz, 1H, OCHPh) 4.55–4.50 (m, 3H, OCHPh, H-1 Glc_a_), 4.49 (d, *J* = 11.6 Hz, 1H, OCHPh), 4.43 (d, *J* = 11.0 Hz, 1H, OCHPh), 4.31 (d, *J* = 12.2 Hz, 1H, OCHPh), 4.15–4.11 (m, 2H), 4.10 (d, *J* = 7.6 Hz, 1H, H-1 Glc_c_), 4.02 (app. t, *J* = 9.3 Hz, 1H), 3.97 (app. t, *J* = 9.2 Hz, 1H), 3.92–3.88 (m, 2H), 3.85–3.79 (m, 3H), 3.72 (dd, *J* = 2.2, 10.8 Hz, 1H), 3.70–3.64 (m, 3H), 3.57 (d, *J* = 9.3 Hz, 1H), 3.53–3.47 (m, 5H), 3.36 (d, *J* = 2.0, 10.8 Hz, 1H), 3.31 (app. t, *J* = 10.4 Hz, 1H), 3.21–3.19 (m, 1H), 2.91 (td, *J* = 5.0, 9.7 Hz, 1H), 1.88 (d, *J* = 4.2 Hz, 1H, OH). ^13^C NMR (150 MHz, CDCl_3_, δ_C_): 170.4 (C=O), 139.2, 138.6, 138.3, 138.2, 138.1, 137.9, 137.7, 137.5, 137.3, 137.1, 135.3, 129.4, 128.7, 128.6, 128.5, 128.4(7), 128.4(1), 128.3(8), 128.3(3), 128.3(0), 128.2(4), 128.2(2), 128.1(6), 128.1(2), 128.1(0), 128.0.127.9, 127.7, 127.6, 127.5(6), 127.5(3), 127.4, 127.2, 127.1, 127.0, 126.8, 126.4, 103.6 (C-1), 102.4 (C-1), 101.8 (C-1), 96.9 (C-1), 96.2 (C-1), 81.6, 80.8, 80.4, 80.2, 80.0, 79.1, 78.8, 78.1, 78.0, 77.6, 75.9, 75.8, 75.0, 74.9, 74.8, 74.1, 73.9, 73.6, 73.5, 73.4, 73.3, 71.1, 70.9, 70.3, 69.7, 68.7, 68.6, 67.8, 67.1, 65.5. HRMS (ESI) calcd for [M + NH_4_]^+^ C_108_H_110_O_22_: 1776.7827; found, 1776.7869.

#### *p*-Methylphenyl benzyl-3,4,6-tri-*O*-benzyl-α-d-glucopyranosyluronate-(1→3)-4,6-*O*-benzylidene-2-*O*-acetyl-1-thio-β-d-glucopyranoside (**4**)

A mixture of **7** (27.5 mg, 0.066 mmol) and **15** (72 mg, 0.099 mmol) was coevaporated three times with anhydrous toluene (3 mL × 3) and then dried in vacuo for 4 h. Activated pulverized 3 Å molecular sieves (150 mg), CH_2_Cl_2_ (3.0 mL) and diethyl ether (1.0 mL) were added and the mixture was stirred at rt for 30 min. The solution was cooled to −30 °C followed by a slow addition of TMSOTf (5.4 μL, 0.029 mmol). After stirring for 1 h at −30 °C, the reaction mixture was neutralized by the addition of Et_3_N (50 μL). The solid was filtered through a pad of Celite and the filtrate was concentrated. The residue was dissolved in EtOAc (20.0 mL) and then washed with saturated NaHCO_3(aq)_, H_2_O and brine. The organic layer was separated, dried over MgSO_4_, filtered and concentrated. The residue was then purified by chromatography (EtOAc–Hexanes 1:5) to afford compound **4** (48 mg, 0.33 mmol, 74% yield, α only) as a white solid. *R*_f_ = 0.35, EtOAc–Hexanes 1:5; ^1^H NMR (600 MHz, CDCl_3_, δ_H_): 7.37–7.36 (m, 3H, ArH), 7.33–7.32 (m, 4H, ArH), 7.30–7.22 (m, 12H, ArH), 7.19–7.07 (m, 8H, ArH), 6.95–6.94 (m, 2H, ArH), 5.60 (d, *J* = 3.4 Hz, 1H, H-1 GlcA), 5.40 (s, 1H, PhCH), 5.17 (d, *J* = 12.0 Hz, 1H, OCHPh), 5.12 (app. t, *J* = 10.0 Hz, 1H, H-2, Glc), 5.08 (d, *J* = 12.0 Hz, 1H, OCHPh), 4.89 (d, *J* = 10.8 Hz, 1H, OCHPh), 4.70 (d, *J* = 10.5 Hz, 1H, OCHPh), 4.68 (d, *J* = 10.0 Hz, 1H, OCHPh), 4.67 (d, *J* = 11.2 Hz, 1H, OCHPh), 4.52 (d, *J* = 12.4 Hz, 1H, OCHPh), 4.35–4.31 (m, 3H, H-3 GlcA, H-6_b_ Glc), 4.27 (d, *J* = 10.0 Hz, 1H, H-5 GlcA), 4.19 (app. t, *J* = 9.2 Hz, 1H, H-3 Glc), 3.86 (app. t, *J* = 9.4 Hz, 1H), 3.82 (app. t, *J* = 9.5 Hz, 1H, H-4 Glc), 3.77 (app. t, *J* = 10.2 Hz, 1H, H-6_a_ Glc), 3.61 (app. t, *J* = 9.4 Hz, 1H, H-4 GlcA), 3.56 (m, 1H, H-5 Glc), 3.44 (dd, *J* = 3.5, 10.0 Hz, 1H, H-2 GlcA), 2.36 (s, 3H, PhCH_3_), 2.06 (s, 3H, CH_3_CO). ^13^C NMR (150 MHz, CDCl_3_, δ_C_): 169.7 (CO), 169.6, 138.6, 138.5, 138.2, 137.6, 136.7, 134.8, 133.4, 129.8, 129.5, 128.7, 128.6, 128.5, 128.3, 128.2, 128.1, 127.9, 127.6, 127.5, 127.4, 127.3, 126.3, 102.0 (PhCH), 96.4 (C-1, GlcA), 87.4 (C-1, Glc), 81.8, 80.5, 79.4, 77.9, 75.8, 75.0, 74.7, 71.5, 70.9, 70.3, 70.2, 68.7, 67.5, 21.2, 20.9. HRMS (ESI) calcd for [M + NH_4_]^+^ C_56_H_60_O_12_NS: 970.3836; found, 970.3851.

#### Benzyl 2,3,6-tri-*O*-benzyl-α-d-glucopyranosyl-(1→3)-2,4,6-tri-*O*-benzyl-β-d-glucopyranoside (**5**)

A mixture of **9** (300 mg, 0.55 mmol) and **8** (498 mg, 0.83 mmol) was coevaporated three times with anhydrous toluene (8 mL × 3) and then dried in vacuo for 4 h. Activated pulverized 3 Å molecular sieves (1.0 g), CH_2_Cl_2_ (15.0 mL) and diethyl ether (5.0 mL) were added and the mixture was stirred at rt for 30 min. Next, solution was cooled to −45 °C and then NIS (247 mg, 1.10 mmol) was added followed by a slow addition of TMSOTf (30 μL, 0.165 mmol). The reaction mixture was stirred at −45 °C for 30 min and then at −20 °C for 90 min. The reaction was neutralized by the addition of Et_3_N (100 μL) and then the solid was filtered through a pad of Celite and the resulting solution was concentrated. The residue was dissolved in EtOAc (50.0 mL) and then washed with saturated Na_2_S_2_O_3(aq)_, H_2_O and brine. The organic layer was separated, dried over MgSO_4_, filtered and concentrated. The residue was then purified by chromatography to afford the disaccharide as an α/β mixture. Next, to a solution of this disaccharide in CH_3_OH (17.0 mL), was added 1 M NaOH (3.0 mL) and the solution was allowed to stir at rt for 12 h. IR-120 (H^+^) resin was added to neutralize the solution. The resin was filtered and the resulting solution was concentrated. The residue was purified by chromatography (EtOAc–Hexanes = 1:5) to afford **5** (326 mg, 0.33 mmol, 61% yield, α only) as a white solid. *R*_f_ = 0.25, EtOAc–Hexanes = 1:5; ^1^H NMR (600 MHz, CDCl_3_, δ_H_): 7.37–7.24 (m, 21H, ArH), 7.23–7.21 (m, 8H, ArH), 7.19–7.14 (m, 4H, ArH), 7.07–7.06 (m, 2H, ArH), 5.55 (d, *J* = 3.5 Hz, 1H, H-1 Glc_b_), 4.97 (d, *J* = 11.5 Hz, 1H, OCHPh), 4.94 (d, *J* = 10.2 Hz, 1H, OCHPh), 4.93 (d, *J* = 11.5 Hz, 1H, OCHPh), 4.92 (d, *J* = 11.4 Hz, 1H, OCHPh), 4.77 (d, *J* = 11.4 Hz, 1H, OCHPh), 4.66 (d, *J* = 12.2 Hz, 1H, OCHPh), 4.65 (d, *J* = 11.2 Hz, 1H, OCHPh), 4.64 (d, *J* = 11.8 Hz, 1H, OCHPh), 4.63 (d, *J* = 12.2 Hz, 1H, OCHPh), 4.58 (d, *J* = 11.8 Hz, 1H, OCHPh), 4.54 (d, *J* = 12.2 Hz, 1H, OCHPh), 4.50 (d, *J* = 11.8 Hz, 1H, OCHPh), 4.50 (d, *J* = 7.8 Hz, 1H, H-1 Glc_a_), 4.45 (d, *J* = 12.2 Hz, 1H, OCHPh), 4.32 (d, *J* = 12.2 Hz, 1H, OCHPh), 4.15 (dt, *J* = 3.6, 10.0 Hz, 1H, H-5, Glc_b_), 3.95 (app. t, *J* = 9.0 Hz, 1H, H-3 Glc_a_), 3.85 (app. t, *J* = 9.4 Hz, 1H, H-3 Glc_b_), 3.79 (app. t, *J* = 9.6 Hz, 1H, H-4 Glc_a_), 3.72 (dd, *J* = 1.9, 10.8 Hz, 1H, H-6_a_ Glc_a_), 3.68 (dd, *J* = 4.4, 10.8 Hz, 1H, H-6_b_ Glc_a_), 3.63 (dt, *J* = 2.6, 9.7 Hz, 1H, H-4 Glc_b_), 3.54 (dt, *J* = 7.8 Hz, 1H, H-2, Glc_a_), 3.50 (dd, *J* = 3.5, 9.8 Hz, 1H, H-2, Glc_b_), 3.48–3.45 (m, 1H, H-5 Glc_a_), 3.40–3.34 (m,2H, H-6 and H-7, Glc_b_), 2.08 (d, *J* = 3.0, 1H, OH). ^13^C NMR (150 MHz, CDCl_3_, δ_C_): 138.8, 138.2(4), 138.2(2), 138.1(6), 138(4) 137.9, 137.4, 128.6, 128.5, 128.4, 128.3, 128.2(8), 128.2(2), 128.1, 127.9, 127.8, 127.7(6), 127.7(3), 127.6(7), 127.6(3), 127.5(9), 127.5, 127.4, 127.0, 102.7 (C-1, Glc_a_), 97.2 (C-1, Glc_b_), 81.4, 80.4, 79.4, 79.2, 78.7, 75.2, 74.6, 74.5, 73.6, 73.5, 73.4, 73.3, 71.2, 71.1, 69.9, 69.2, 68.7. HRMS (ESI) calcd for [M + NH_4_]^+^ C_61_H_68_NO_11_: 990.4803; found, 990.4787.

#### Benzyl 2,4,6-tri-*O*-benzyl-β-d-glucopyranoside (**9**)

To a solution of **12** (380 mg, 0.87 mmol) in CH_3_OH (10 mL), was added NaOCH_3_ (15 mg, 0.25 mmol). The reaction mixture was stirred at rt for 4 h and then quenched by the addition of IR-120 (H^+^) resin. The resin was filtered, the resulting solution was concentrated and the residue was then dried in vacuo for 3 h. The residue was dissolved in DMF (10 mL) and the solution was cooled to 0 °C before NaH (118 mg, 4.91 mmol) was added. After stirring for 1 h, benzyl bromide (380 μL, 3.2 mmol) was added slowly. The reaction mixture was gradually warmed to rt and stirred for 12 h. Next, saturated NH_4_Cl_(aq)_ was added to neutralize the reaction mixture, followed by evaporation of the reaction solvent. The residue was diluted with EtOAc (30 mL) and the resulting organic solution was washed with H_2_O and brine. The organic layer was separated, dried over MgSO_4_, filtered and concentrated. The residue was purified by chromatography (EtOAc–Hexanes = 1:5, *R*_f_ = 0.40) to afford the benzylated intermediate (480 mg, 0.827 mmol, 95%). Next, to a solution of the benzylated intermediate in THF (11.0 mL), hydrogen-activated [Ir­(cod)­(Ph_2_MeP)_2_]­PF_6_ (63 mg, 0.074 mmol, dissolved in 5 mL THF) was added and the solution was stirred at rt for 45 min. The reaction solvent was evaporated and the residue was dried in vacuo for 1 h. The residue obtained was dissolved in acetone–water (15 mL, 10:1), followed by the addition of HgO (72 mg, 0.331 mmol) and HgCl_2_ (494 mg, 1.82 mmol). The solution was stirred at rt for 2 h and then concentrated. The residue was purified by chromatography (EtOAc–Hexanes = 1:5) to afford **9** (372 mg, 0.69 mmol, 79% yield) as a colorless oil. *R*_f_ = 0.25, EtOAc–Hexanes = 1:5; ^1^H NMR (600 MHz, CDCl_3_, δ_H_): 7.40–7.25 (m, 20H, ArH), 5.00 (d, *J* = 11.0 Hz, 1H, OCHPh), 4.98 (d, *J* = 11.0 Hz, 1H, OCHPh), 4.86 (d, *J* = 11.2 Hz, 1H, OCHPh), 4.68 (d, *J* = 11.2 Hz, 1H, OCHPh), 4.67 (d, *J* = 12.0 Hz, 1H, OCHPh) 4.64 (d, *J* = 12.0 Hz, 1H, OCHPh), 4.58 (d, *J* = 11.2 Hz, 1H, OCHPh), 4.57 (d, *J* = 12.0 Hz, 1H, OCHPh), 4.52 (d, *J* = 7.8 Hz, 1H, H-1), 3.80 (dd, *J* = 1.9, 10.7 Hz, 1H, H-6a), 3.75–3.71 (m, 2H, H-6b, H-3), 3.58 (app. t, *J* = 9.0 Hz, 1H, H-4), 3.50–3.47 (m, 1H, H-5), 3.38 (app. t, *J* = 7.8 Hz, 1H, H-2). 2.47 (br s, 1H, OH). ^13^C NMR (150 MHz, CDCl_3_, δ_C_): 138.3(3), 138.3(2), 138.0, 137.4, 128.5, 128.5, 128.2, 128.0, 127.9, 127.8, 127.7, 127.7, 102.2 (C-1), 81.2, 77.2, 76.7, 74.8, 74.5, 74.4, 73.5, 71.1, 68.8. HRMS (ESI) calcd for [M + NH_4_]^+^ C_34_H_40_O_6_N: 558.2864; found, 558.2850.

#### 3-*O*-Allyl-1,2,4,6-tetra-*O*-acetyl-d-glucopyranose (**10**)

To a solution of diacetone-d-glucose (5.000 g, 19.230 mmol) in THF (100 mL) was added NaH (830 mg, 34.6 mmol) at 0 °C and then the mixture was stirred for 20 min followed by slow addition of allyl bromide (1.99 mL, 23.076 mmol). The reaction mixture was gradually warmed to rt and stirred for 15 h. Next, excess NaH was quenched by the addition of saturated NH_4_Cl_(aq)_ and then the solvent was evaporated. The residue was diluted with EtOAc (100 mL) and the resulting organic solution was washed with H_2_O and brine. The organic layer was dried over MgSO_4_, filtered and concentrated. Next, to a solution of this residue in acetic acid (40 mL) was added H_2_O (10 mL). The reaction mixture was stirred at 80 °C for 12 h, then it was cooled to rt and concentrated. The remaining acetic acid from the mixture was removed by coevaporation with toluene (10 mL × 3 times) and further drying in vacuo for 2 h. The residue obtained was dissolved in pyridine (20 mL), cooled to 0 °C then acetic anhydride (11.768 mL, 115.35 mmol) was added followed by the addition of DMAP (446.13 mg, 1.923 mmol). The reaction mixture was gradually warmed to rt and stirred for 12 h before being concentrated. The residue was diluted with EtOAc (100 mL) and the resulting organic solution was washed with 1 N HCl_(aq)_, saturated NaHCO_3(aq),_ H_2_O and brine. The organic layer was separated, dried over MgSO_4_, filtered and concentrated. The residue was purified by chromatography (EtOAc–Hexanes = 1:1) to afford compound **10** (6.3 g, 85% over 3 steps, 9:1 β/α) as a white solid. *R*_f_ = 0.55, EtOAc–Hexanes = 1:1; for β isomer: ^1^H NMR (500 MHz, CDCl_3_, δ_H_): 5.76–5.68 (m, 1H), 5.59 (d, *J* = 8.0 Hz, 1H, H-1), 5.17–5.08 (m, 2H), 5.06–5.01 (m, 2H), 4.18–4.15 (m, 1H), 4.05–4.03 (m, 3H), 3.69–3.68 (m, 1H), 3.60 (app. t, *J* = 8.0 Hz, 1H, H-4), 2.04 (s, 3H, CH_3_CO), 2.03 (s, 3H, CH_3_CO), 2.02 (s, 6H, CH_3_CO). ^13^C NMR (125 MHz, CDCl_3_, δ_C_): 170.5 (C=O), 169.1 (C=O), 169.0 (C=O), 168.9 (C=O), 133.9, 117.0, 91.8 (C-1), 79.5, 73.0, 72.8, 71.3, 68.9, 61.7, 20.7, 20.66, 20.6, 20.58. For α isomer: ^1^H NMR (500 MHz, CDCl_3_, δ_H_): 6.24 (d, *J* = 2.4 Hz, 1H, H-1), 5.76–5.68 (m, 1H), 5.17–5.08 (m, 2H), 5.06–5.01 (m, 2H), 4.96–4.94 (m, 1H), 4.18–4.15 (m, 1H), 4.05–4.03 (m, 3H), 3.96–3.95 (m, 1H), 3.79 ((app. t, *J* = 9.5 Hz, 1H, H-4), 2.11 (s, 3H, CH_3_CO), 2.02 (s, 6H, CH_3_CO) 1.87 (s, 3H, CH_3_CO) ^13^C NMR (125 MHz, CDCl_3_, δ_C_): 170.5 (C=O), 169.1 (C=O), 169.0 (C=O), 168.9 (C=O), 134.2, 116.6, 89.3 (C-1), 76.4, 73.4,71.2, 70.1, 63.1, 20.7, 20.66, 20.6, 20.58. HRMS (ESI) calcd for [M + NH_4_]^+^ C_17_H_28_NO_10_: 406.1715; found, 406.1708.

#### 3-*O*-Allyl-2,4,6-tri-*O*-acetyl-d-glucopyranosyl trichloroacetimidate (**11**)

To a solution of **10** (4.700 g, 12.05 mmol)) in THF (100 mL) was added hydrazine acetate (1.662 g, 18.075 mmol) and then the solution was stirred at rt for 4 h before being concentrated. The residue was diluted with EtOAc (100 mL) and the resulting organic solution was washed with H_2_O and brine. The organic layer was dried over MgSO_4_, filtered and concentrated. The residue was purified by chromatography (EtOAc–Hexanes = 1:2, *R*_f_ = 0.25) to afford the hemiacetal intermediate (3.15 g, 9.27 mmol, 77%). To a solution of the hemiacetal intermediate from the previous step (480 mg, 1.41 mmol) in CH_2_Cl_2_ (14 mL) was added trichloroacetonitrile (580 μL, 5.64 mmol) and the solution was cooled to 0 °C. Next, DBU (32.15 μL, 0.21 mmol, dissolved in 1.0 mL CH_2_Cl_2_) was added and the mixture was stirred 30 min and then concentrated. The residue was purified by chromatography (EtOAc–Hexanes = 1:2) to afford **11** (610 mg, 1.2 mmol, 85%, only α isomer isolated) as a white solid. *R*_f_ = 0.55, EtOAc–Hexanes = 1:2; ^1^H NMR (500 MHz, CDCl_3_, δ_H_): 8.6 (s, 1H, NH), 6.48 (d, *J* = 3.5 Hz, 1H, H-1), 5.81–5.73 (m, 1H, CH_2_=CH), 5.18 (dd, *J* = 1.4, 17.0 Hz, 1H, CH_2_=CH), 5.12 (d, *J* = 10 Hz, 1H, CH_2_=CH), 5.10 (app. t, *J* = 9.5 Hz, 1H, H-4), 4.99 (dd, *J* = 3.6, 10.0 Hz, 1H, H-2), 4.18–4.14 (m, 2H), 4.08–4.05 (m, 3H), 3.93 (app. t, *J* = 10 Hz, 1H, H-3), 2.06 (s, 3H, CH_3_CO), 2.03 (s, 3H, CH_3_CO), 2.01 (s, 3H, CH_3_CO). ^13^C NMR (125 MHz, CDCl_3_, δ_C_): 170.6 (C=O), 169.7 (C=O), 169.2 (C=O), 160.6, 134.2, 116.9, 93.4 (C-1), 90.9 (CCl_3_), 76.5, 73.7, 71.9, 70.5, 68.9, 61.7, 20.8, 20.7, 20.6. HRMS (ESI) calcd for [M + H]^+^ C_17_H_23_Cl_3_NO_9_: 490.0433; found, 490.0433.

#### Benzyl 3-*O*-allyl-2,4,6-tri-*O*-acetyl-benzyl-β-d-glucopyranoside (**12**)

A solution of **11** (502 mg, 1.02 mmol), benzyl alcohol (133 μL, 1.23 mmol) and activated pulverized 3 Å molecular sieve (630 mg) in CH_2_Cl_2_ (15 mL) was stirred at rt for 1 h. The solution was cooled to −30 °C and then TMSOTf (37 μL, 0.2 mmol) was added slowly. The reaction mixture was stirred at −30 °C for 1 h and then Et_3_N (100 μL) was added. The solution was filtered through a pad of Celite and the resulting solution was concentrated. The residue was dissolved in EtOAc and then washed with saturated NaHCO_3(aq)_, H_2_O and brine. The organic layer was separated, dried over MgSO_4_, filtered and concentrated. The residue was then purified by chromatography (EtOAc–Hexanes = 1:3) to afford **12** (397 mg, 0.91 mmol, 89%) as a colorless oil. *R*_f_ = 0.10, EtOAc–Hexanes = 1:3; ^1^H NMR (500 MHz, CDCl_3_, δ_H_): 7.35–7.27 (m, 5H, ArH), 5.78–5.71 (m, 1H, CH_2_=CH), 5.18 (dd, *J* = 1.5, 17.0 Hz, 1H, CH_2_=CH) 5.11 (dd, *J* = 1.5, 10.0 Hz, 1H, CH_2_=CH) 5.06 (app. t, *J* = 9.5 Hz, 1H, H-4), 5.05 (app. t, *J* = 9.5 Hz, 1H, H-2), 4.87 (d, *J* = 12.4 Hz, 1H, OCH_2_CH=CH_2_), 4.59 (d, *J* = 12.4 Hz, 1H, OCH_2_CH=CH_2_), 4.44 (d, *J* = 8.0 Hz, 1H, H-1), 4.22 (dd, *J* = 5.1, 12.0 Hz, 1H, H-6a), 4.14 (dd, *J* = 2.5, 12.0 Hz, 1H, H-6b), 4.05–4.03 (m, 2H, OCH_2_Ph), 3.57–3.53 (m, 2H, H-3, H-5), 2.09 (s, 3H, CH_3_CO), 2.06 (s, 3H, CH_3_CO), 2.04 (s, 3H, CH_3_CO). ^13^C NMR (125 MHz, CDCl_3_, δ_C_): 170.7­(C=O), 169.3 (C=O), 169.1 (C=O), 136.9, 134.2, 128.4, 127.8, 127.7, 116.9, 99.4 (C-1), 79.7, 72.7, 72.4, 72.0, 70.4, 69.6, 62.3, 20.8, 20.77, 20.73. HRMS (ESI) calcd for [M + NH_4_]^+^ C_22_H_32_NO_9_: 454.2082; found, 454.2072.

#### *p*-Methylphenyl benzyl-2,3,4-*O*-benzyl-1-thio-β-d-glucopyranosyluronate (**14**)

To a solution of **13** (500 mg, 0.92 mmol) in THF (9.0 mL) was added 1 M BH_3_·THF (2.7 mL, 2.7 mmol). The solution was cooled to 0 °C, followed by slow addition of TMSOTf (41.7 μL, 0.225 mmol). The reaction mixture was gradually warmed to rt and stirred for 3 h, at which point Et_3_N (400 μL) was added and the solution was concentrated. The crude mixture was then purified by chromatography (EtOAc–Hexanes = 1:5, *R*_f_ = 0.55) to afford a colorless oily intermediate (415 mg, 0.75 mmol). The residue obtained was dissolved in CH_2_Cl_2_–H_2_O (12.0 mL, 3:1), cooled to 0 °C and then bis-acetoxyiodobenzene (603 mg, 1.87 mmol) and TEMPO (18 mg, 0.11 mmol) were added. The reaction mixture was gradually warmed to rt and stirred for 4 h before being concentrated. The residue was diluted with EtOAc (30 mL) and the resulting organic solution was washed with saturated Na_2_S_2_O_3(aq)_, H_2_O and brine. The organic layer was separated, dried over MgSO_4_, filtered and concentrated. The crude residue obtained was dried in vacuo for 2 h and then dissolved in DMF (10 mL). Next, NaHCO_3_ (252 mg, 3.0 mmol) and BnBr (184 μL, 1.5 mmol) were added and then solution was to stirred for 6 h. The reaction mixture was concentrated, the residue diluted with EtOAc (30 mL) and the resulting organic solution was washed with H_2_O and brine. The organic layer was separated, dried over MgSO_4_, filtered and concentrated. The residue was purified by chromatography (EtOAc–Hexanes = 1:5) to afford compound **14** (382 mg, 0.69 mmol, 76% over three steps) as a white foam. *R*_f_ = 0.44, EtOAc–Hexanes = 1:5; ^1^H NMR (500 MHz, CDCl_3_, δ_H_): 7.53–7.51 (m, 2H, ArH), 7.46–7.44 (m, 2H, ArH), 7.41–7.30 (m, 16H, ArH), 7.20–7.19 (m, 2H, ArH), 7.13–7.11 (m, 2H, ArH), 5.24 (s, 2H, OCH_2_Ph), 4.96 (d, *J* = 10.4 Hz, 1H, OCHPh), 4.92 (d, *J* = 11.0 Hz, 1H, OCHPh), 4.88 (d, *J* = 11.0 Hz, 1H, OCHPh), 4.78 (d, *J* = 10.8 Hz, 2H, OCHPh), 4.68 (d, *J* = 9.8 Hz, 1H, H-1), 4.58 (d, *J* = 10.8 Hz, 1H, OCHPh), 4.00 (d, *J* = 9.8 Hz, 1H, H-5), 3.90 (app. t, *J* = 9.5 Hz, 1H, H-4), 3.76 (app. t, *J* = 9.0 Hz, 1H, H-3), 3.56 (app. t, *J* = 9.3 Hz, 1H, H-2), 2.38 (s, 3H, PhCH_3_). ^13^C NMR (125 MHz, CDCl_3_, δ_C_): 168.1 (C=O), 138.2, 138.0, 137.8, 135.1, 133.1, 129.8, 129.0, 128.6, 128.5, 128.4, 128.2, 128.0, 127.9, 127.8, 88.4 (C-1), 85.9, 80.2, 79.3, 78.2, 75.9, 75.5, 75.1, 67.3. HRMS (ESI) calcd for [M + H]^+^ C_41_H_41_O_6_S: 661.2618; found, 661.2618.

#### Benzyl 2,3,4-*O*-benzyl-d-glucopyranosyluronyl trichloroacetimidate (**15**)

To a solution of **14** (800 mg, 1.21 mmol) in acetone–water (15 mL, 4:1) was added NIS (545 mg, 2.42 mmol) and the solution was stirred at rt for 2 h. Excess NIS was quenched by the addition of saturated Na_2_S_2_O_3(aq)_ and the solvent was evaporated. The residue was diluted with EtOAc (40 mL) and the resulting organic solution was washed with H_2_O and brine. The organic layer was dried over MgSO_4_, filtered and concentrated. The crude mixture was then purified by chromatography (EtOAc–Hexanes = 1:5, *R*_f_ = 0.10) to afford hemiacetal intermediate (609 mg, 1.09 mmol, 90% yield). Next, to a solution of the hemiacetal intermediate from the previous step in CH_2_Cl_2_ (15 mL) was added trichloroacetonitrile (436 μL, 4.36 mmol) and the solution was cooled to 0 °C before DBU (25 μL, 0.16 mmol, dissolved in 1.0 mL CH_2_Cl_2_) was added. The reaction mixture was gradually warmed to rt and stirred for 2 h before being concentrated. The residue was purified by chromatography (EtOAc–Hexanes = 1:2) to afford **15** (668 mg, 0.95 mmol, 90% yield, α/β 10:1) as a white foam. *R*_f_ = 0.40, EtOAc–Hexanes = 1:2; for α isomer: ^1^H NMR (600 MHz, CDCl_3_, δ_H_): 8.68 (s, 1H, NH), 7.31–7.28 (m, 15H, ArH), 7.26–7.25 (m, 3H, ArH), 7.12–7.11 (m, 2H, ArH), 6.53 (d, *J* = 3.5 Hz, 1H, H-1), 5.16 (d, *J* = 12.2 Hz, 1H, OCHPh), 5.14 (d, *J* = 12.0 Hz, 1H, OCHPh), 4.93 (d, *J* = 10.8 Hz, 1H, OCHPh), 4.81 (d, *J* = 10.8 Hz, 1H, OCHPh), 4.75 (d, *J* = 10.8 Hz, 1H, OCHPh), 4.74 (d, *J* = 11.6 Hz, 1H, OCHPh), 4.69 (d, *J* = 11.6 Hz, 1H, OCHPh), 4.45 (d, *J* = 10.0 Hz, 1H, H-5), 4.44 (d, *J* = 10.6 Hz, 1H, OCHPh), 4.06 (app. t, *J* = 9.4 Hz, 1H, H-3), 3.83 (app. t, *J* = 9.8 Hz, 1H, H-4), 3.80 (dd, *J* = 3.5, 9.4 Hz, 1H, H-2). ^13^C NMR (150 MHz, CDCl_3_, δ_C_): 168.6 (C=O), 161.1, 138.3, 137.7, 137.6, 134.9, 128.6, 128.5(4), 128.5(1), 128.4(6), 128.4(0), 128.3(9), 128.1, 128.0, 127.9, 127.8, 127.7, 127.3, 94.0 (C-1), 91.0, 80.4, 78.9, 78.8, 75.8, 75.3, 73.1, 72.8, 67.4. For β isomer: ^1^H NMR (600 MHz, CDCl_3_, δ_H_): 8.74 (s, 1H, NH), 7.31–7.28 (m, 15H, ArH), 7.26–7.25 (m, 3H, ArH), 7.12–7.11 (m, 2H, ArH), 5.89 (d, *J* = 7.2 Hz, 1H, H-1), 5.16 (d, *J* = 12.2 Hz, 1H, OCHPh), 5.14 (d, *J* = 12.0 Hz, 1H, OCHPh), 4.92 (d, *J* = 11.2 Hz, 1H, OCHPh), 4.86 (d, *J* = 11.2 Hz, 1H, OCHPh), 4.79 (d, *J* = 11.2 Hz, 1H, OCHPh), 4.74 (d, *J* = 11.5 Hz, 1H, OCHPh), 4.72 (d, *J* = 10.2 Hz, 1H, OCHPh), 4.49 (d, *J* = 10.8 Hz, 1H, OCHPh), 4.15 (d, *J* = 9.8 Hz, 1H, H-5), 3.95 (app. t, *J* = 9.6 Hz, 1H, H-4), 3.83 (app. t, *J* = 9.8 Hz, 1H, H-3) 3.78 (app. t, *J* = 8.4 Hz, 1H, H-2). ^13^C NMR (150 MHz, CDCl_3_, δ_C_): 168.1 (C=O), 160.9, 138.1, 137.7, 137.6, 134.9, 128.6, 128.54, 128.51, 128.46, 128.40, 128.39, 128.1, 128.0, 127.9, 127.8, 127.75, 127.3, 98.0 (C-1), 91.0, 83.6, 80.4, 78.9, 78.8, 75.6, 74.9, 74.8, 67.4. HRMS (ESI) calcd for [M + NH_4_]^+^ C_36_H_38_Cl_3_O_7_N_2_: 715.1739; found, 715.1759.

#### Benzyl (benzyl 3,4,6-tri-*O*-benzyl-α-d-glucopyranosyluronate)-(1→3)-4,6-*O*-benzylidene-2-*O*-acetyl-β-d-glucopyranosyl-(1→4)-2,3,6-tri-*O*-benzyl-α-d-glucopyranosyl-(1→3)-2,4,6-tri-*O*-benzyl-β-d-glucopyranoside (**16**)

A mixture of **5** (28.0 mg, 0.029 mmol) and **4** (36 mg, 0.038 mmol) was coevaporated three times with anhydrous toluene (3 mL × 3) then dried in vacuo for 4 h. Activated pulverized 3 Å molecular sieves (150 mg) and CH_2_Cl_2_ (3.0 mL) were added and the mixture was stirred at rt for 30 min. The solution was cooled to −45 °C, and then NIS (10 mg, 0.043 mmol) was added followed by slow addition of TMSOTf (2.1 μL, 0.11 mmol). The reaction mixture was stirred at −45 °C for 30 min and then at −20 °C for 90 min before Et_3_N (50 μL) was added. The mixture was filtered through a pad of Celite and the resulting solution was concentrated. The residue was dissolved in EtOAc (20.0 mL) and then washed with saturated Na_2_S_2_O_3(aq)_, H_2_O and brine. The organic layer was separated, dried over MgSO_4_, filtered and concentrated. The residue was then purified by chromatography to (EtOAc–Hexanes = 1:3) to afford **16** (46 mg, 0.025 mmol, 88% yield) as a white solid. *R*_f_ = 0.30, EtOAc–Hexanes = 1:3; ^1^H NMR (600 MHz, CDCl_3_, δ_H_): 7.37–7.26 (m, 28H, ArH), 7.25–7.22 (m, 15H, ArH), 7.20–7.14 (m, 5H, ArH), 7.12–7.08 (m, 8H, ArH), 7.05–7.04 (m, 2H, ArH), 6.96–6.95 (m, 2H, ArH), 5.58 (d, *J* = 3.3 Hz, 1H, H-1, GlcA), 5.49 (d, *J* = 3.7 Hz, 1H, H-1, Glc_b_), 5.25 (s, 1H, PhCH), 5.17 (d, *J* = 12.2 Hz, 1H, OCHPh), 5.06 (d, *J* = 12.2 Hz, 1H, OCHPh), 5.04 (app. t, *J* = 9.0 Hz, 1H, H-2, Glc_c_), 4.97 (d, *J* = 11.2 Hz, 1H, OCHPh), 4.92 (d, *J* = 11.7 Hz, 1H, OCHPh), 4.91 (d, *J* = 11.2 Hz, 1H, OCHPh), 4.89 (d, *J* = 11.0 Hz, 1H, OCHPh), 4.84 (d, *J* = 11.2 Hz, 1H, OCHPh), 4.79 (d, *J* = 11.2 Hz, 1H, OCHPh), 4.70 (d, *J* = 11.2 Hz, 1H, OCHPh), 4.69 (d, *J* = 10.8 Hz, 1H, OCHPh), 4.68 (d, *J* = 11.6 Hz, 1H, OCHPh), 4.64 (d, *J* = 11.8 Hz, 1H, OCHPh), 4.63 (d, *J* = 12.0 Hz, 1H, OCHPh), 4.60 (d, *J* = 12.2 Hz, 1H, OCHPh), 4.57 (d, *J* = 11.6 Hz, 1H, OCHPh), 4.55–4.50 (m, 3H, OCHPh), 4.47 (d, *J* = 7.8 Hz, 1H, H-1, Glc_a_) 4.43 (d, *J* = 11.4 Hz, 1H, OCHPh), 4.37 (d, *J* = 8.0 Hz, 1H, H-1, Glc_c_), 4.33 (d, *J* = 12.2 Hz, 1H, OCHPh), 4.31 (d, *J* = 11.2 Hz, 1H, OCHPh), 4.20 (d, *J* = 11.7 Hz, 1H, OCHPh), 4.17 (d, *J* = 9.8 Hz, 1H, H-5 GlcA), 4.16–4.14 (m, 1H), 4.05 (d, *J* = 5.0, 10.6 Hz, 1H), 3.91–3.81 (m, 5H), 3.72–3.64 (m, 5H), 3.62–3.57 (m, 2H), 3.50–3.46 (m, 3H, H-2 GlcA, H-2 Glc_b_), 3.44–3.41 (m, 1H), 3.31 (app. t, *J* = 10.4 Hz, 1H), 3.01 (td, *J* = 5.0, 5.0, 10.0 Hz, 1H), 1.87 (s, 3H, CH_3_CO). ^13^C NMR (150 MHz, CDCl_3_, δ_C_): 169.7 (C=O), 169.2 (C=O), 139.4, 138.5, 138.4, 138.3, 138.2, 138.1, 137.8, 137.6, 137.3, 136.8, 134.8, 129.5, 128.8, 128.7, 128.6, 128.5, 128.4, 128.3, 128.2(8), 128.2(2), 128.1(9), 128.1(3), 128.07, 128.02, 127.8(5), 127.8(3), 127.6(7), 127.6(4), 127.5(9), 127.5(5), 127.5(2), 127.4, 127.3(5), 127.3(0), 127.1, 126.3, 102.8 (C-1 Glc_a_), 101.8 (PhCH) 100.7 (C-1, Glc_c_), 97.6 (C-1, Glc_b_), 96.2 (C-1, GlcA), 82.1, 81.3, 80.4, 79.7, 79.57, 79.51, 79.1, 78.1, 77.9, 75.8, 75.0, 74.7, 74.1, 73.9, 73.6, 73.5, 73.2, 68.8, 68.6, 67.7, 67.5, 65.4, 20.8. HRMS (ESI) calcd for [M + NH_4_]^+^ C_110_H_116_O_23_N: 1818.7933; found, 1818.8024.

#### Benzyl 3-*O*-allyl-4,6-*O*-benzylidene-β-d-glucopyranosyl-(1→4)-2,3,6-tri-*O*-benzyl-α-d-glucopyranosyl-(1→3)-2,4,6-tri-*O*-benzyl-β-d-glucopyranoside (**18**)

A mixture of **9** (150.0 mg, 0.154 mmol) and **11** (120 mg, 0.231 mmol) was coevaporated three times with anhydrous toluene (10 mL × 3) then dried in vacuo for 4 h. Activated pulverized 3 Å molecular sieve (500 mg) and CH_2_Cl_2_ (10.0 mL) were added and the mixture was stirred at rt for 30 min. The solution was cooled to −45 °C and then TMSOTf (11.5 μL, 0.61 mmol) was slowly added. The reaction mixture was stirred at −45 °C for 30 min and then at −30 °C for 90 min before Et_3_N (100 μL) was added. The mixture was filtered through a pad of Celite and the resulting solution was concentrated. The residue was dissolved in EtOAc (50.0 mL) and then washed with H_2_O and brine. The organic layer was separated, dried over MgSO_4_, filtered and concentrated. The residue was then purified by chromatography (EtOAc–Hexanes = 1:3, *R*_f_ = 0.10) to afford the corresponding trisaccharide (160 mg, 0.123 mmol, 80% yield). To a solution of this trisaccharide (160.0 mg, 0.123) in CH_3_OH (8.5 mL), was added 1 M NaOH (1.5 mL) and the solution was allowed to stir at rt for 12 h before being concentrated. The residue was dissolved in EtOAc (50.0 mL) and then washed with H_2_O and brine. The organic layer was dried over MgSO_4_, filtered and concentrated. The crude residue obtained was dried for 2 h and then dissolved in CH_3_CN (10.0 mL). To the solution was added benzaldehyde dimethyl acetal (38 μL, 0.246 mmol) and camphorsulfonic acid (11.4 mg, 0.049 mmol) and the reaction mixture was stirred at 45 °C for 4 h before being gradually warmed to rt. At this point, Et_3_N (200 μL) was added and the solution was concentrated. The residue was dissolved in EtOAc (50.0 mL) and then washed with H_2_O and brine. The organic layer was separated, dried over MgSO_4_, filtered and concentrated. The residue was then purified by chromatography (EtOAc–Hexanes = 1:3) to afford **18** (130 mg, 0.103 mmol, 87% yield over three steps) as a white solid. *R*_f_ = 0.45, EtOAc–Hexanes = 1:3; ^1^H NMR (600 MHz, CDCl_3_, δ_H_): 7.46–7.44 (m, 2H, ArH), 7.38–7.21 (m, 30H, ArH), 7.19–7.15 (m, 2H, ArH), 7.13–7.08 (m, 4H, ArH), 7.07–7.05 (m, 2H, ArH), 5.96–5.89 (m, 1H, CH_2_=CH), 5.54 (d, *J* = 3.6 Hz, 1H, H-1 Glc_b_), 5.42 (s, 1H, PhCH), 5.32 (dd, *J* = 1.7, 17.0 Hz, 1H, CH_2_=CH), 5.19 (dd, *J* = 1.7, 10.5 Hz, 1H, CH_2_=CH), 4.97 (d, *J* = 11.0 Hz, 1H, OCHPh), 4.96 (d, *J* = 12.0 Hz, 1H, OCHPh), 4.94 (d, *J* = 11.5 Hz, 2H, OCHPh), 4.83 (d, *J* = 11.2 Hz, 1H, OCHPh), 4.71 (d, *J* = 10.8 Hz, 1H, OCHPh), 4.66 (d, *J* = 11.6 Hz, 1H, OCHPh), 4.65 (d, *J* = 12.0 Hz, 1H, OCHPh), 4.63 (d, *J* = 12.2 Hz, 2H, OCHPh), 4.56 (d, *J* = 11.8 Hz, 1H, OCHPh), 4.52 (d, *J* = 11.8 Hz, 2H, OCHPh), 4.51 (d, *J* = 7.7 Hz, 1H, H-1 Glc_a_), 4.47 (d, *J* = 11.5 Hz, 1H, OCHPh), 4.44 (d, *J* = 7.4 Hz, 1H, H-1 Glc_c_), 4.38–4.35 (m, 2H, OCHPh, OCH_2_CH=CH_2_), 4.26–4.21 (m, 2H, OCH_2_CH=CH_2_), 3.97–3.91 (m, 4H, H-3 Glc_a_, H-4 Glc_b_, H6_a_ Glc_c_), 3.78 (app. t, *J* = 9.3 Hz, 1H, H-3 Glc_b_), 3.72 (dd, *J* = 1.8, 10.8 Hz, 1H, H6_b_ Glc_c_), 3.68 (dd, *J* = 4.2, 10.8 Hz, 1H, H6_a_ Glc_b_), 3.64 (dd, *J* = 2.6, 11.3 Hz, 1H, H-6_a_ Glc_a_), 3.52 (app. t, *J* = 8.4 Hz, 1H, H-2 Glc_a_) 3.51 (dd, 3.6, 9.5 Hz, 1H, H-2 Glc_b_), 3.48–3.45 (m,1H), 3.46 (app. t, *J* = 9.2 Hz, 1H, H-4Glc_c_), 3.43–3.41 (m, 1H, H-6_b_ Glc_a_), 3.41 (app. t, *J* = 10.5 Hz, 1H, H-4 Glc_a_), 3.33 (app. t, *J* = 9.0 Hz, 1H, H-3 Glc_c_), 3.30 (td, *J* = 2.4, 9.0 Hz, 1H, H-2 Glc_c_), 3.06 (td, *J* = 5.0, 9.7 Hz, 1H, H-5 Glc_a_), 2.65 (d, *J* = 2.6 Hz, 1H, OH). ^13^C NMR (150 MHz, CDCl_3_, δ_C_): 139.3, 138.4, 138.2, 138.1, 137.9, 137.7, 137.4, 137.3, 135.2, 129.0, 129.9, 128.4, 128.3, 128.2, 128.16, 128.13, 127.9, 127.8, 127.83, 127.81, 127.7, 127.6, 127.58, 127.51, 127.3, 127.2, 127.12, 126.9, 126.0, 116.8, 103.7 (C-1, Glc_c_), 102.6 (C-1, Glc_a_), 101.0 (PhCH), 97.2 (C-1, Glc_b_), 81.2, 80.7, 80.2, 80.1, 79.4, 79.2, 78.7, 77.6, 75.1, 74.8, 74.7, 74.1, 73.8, 73.7, 73.5, 73.4, 71.0, 69.8, 68.7, 68.6, 68.1, 66.1. HRMS (ESI) calcd for [M + Na]^+^ C_77_H_82_NaO_16_: 1285.5495; found, 1285.5504.

#### Benzyl 3-*O*-allyl-4,6-*O*-benzylidene-β-d-mannopyranosyl-(1→4)-2,3,6-tri-*O*-benzyl-α-d-glucopyranosyl-(1→3)-2,4,6-tetra-*O*-benzyl-β-d-glucopyranoside (**19**)

To a solution of **18** (80 mg, 0.063 mmol) in CH_2_Cl_2_ (4.0 mL) was added Dess–Martin Periodinane (DMP, 80 mg, 0.19 mmol). The resultant mixture was then stirred at rt for 2 h before excess DMP was quenched by the addition of saturated Na_2_S_2_O_3(aq)_. To this mixture was added CH_2_Cl_2_ and the resultant mixture was then washed with H_2_O, saturated NaHCO_3(aq)_ and brine. The organic layer was dried over MgSO_4_, filtered and concentrated. The crude residue obtained was coevaporated with toluene (3 mL × 3) and then dried in vacuo for 1 h. The crude residue was redissolved in THF (4.0 mL) and the resultant solution was subsequently cooled to −15 °C. Next, lithium tritert-butoxyaluminum hydride (LTBA, 96 μL, 0.378 mmol) was added slowly and the mixture was stirred at −15 °C for 2 h before AcOH (200 μL) was added to quench the excess LTBA. Then reaction mixture was gradually warmed to rt and concentrated and the residue was dissolved in EtOAc (25 mL) and then washed with saturated NaHCO_3(aq)_, H_2_O and brine. The organic layer was dried over MgSO_4_, filtered and concentrated. The residue was then purified by chromatography (EtOAc–Hexanes = 1:3) to afford **19** (63 mg, 0.050 mmol, 80% yield over two steps) as a white solid. *R*_f_ = 0.20, EtOAc–Hexanes = 1:3; ^1^H NMR (600 MHz, CDCl_3_, δ_H_): 7.46–7.44 (m, 2H, ArH), 7.38–7.27 (m, 25H, ArH), 7.24–7.19 (m, 6H, ArH), 7.17–7.14 (m, 1H, ArH), 7.12–7.07 (m, 4H, ArH), 7.03–7.02 (m, 2H, ArH), 5.99–5.92 (m, 1H, CH_2_=CH), 5.53 (d, *J* = 3.7 Hz, 1H, H-1 Glc_b_), 5.45 (s, 1H, PhCH), 5.33–5.29 (m,1H, CH_2_=CH), 5.21–5.19 (m, 1H, CH_2_=CH), 5.05 (d, *J* = 10.8 Hz, 1H, OCHPh), 4.97 (d, *J* = 10.0 Hz, 1H, OCHPh), 4.96 (d, *J* = 11.5 Hz, 1H, OCHPh), 4.92 (d, *J* = 11.5 Hz, 1H, OCHPh), 4.78 (d, *J* = 10.8 Hz, 1H, OCHPh), 4.70 (d, *J* = 11.6 Hz, 1H, OCHPh), 4.66 (d, *J* = 11.6 Hz, 1H, OCHPh), 4.62 (d, *J* = 12.1 Hz, 1H, OCHPh), 4.61 (d, *J* = 10.7 Hz, 1H, OCHPh), 4.58 (d, *J* = 12.2 Hz, 1H, OCHPh), 4.53 (d, *J* = 12.2 Hz, 1H, OCHPh), 4.52 (d, *J* = 11.6 Hz, 1H, OCHPh), 4.52 (d, *J* = 7.8 Hz, 1H, H-1 Glc_a_), 4.44 (d, *J* = 11.6 Hz, 1H, OCHPh), 4.34 (s, 1H, H-1 Man), 4.24–4.20 (m, 1H, OCH_2_CH=CH_2_), 4.17 (d, *J* = 12.0 Hz, 1H, OCHPh), 4.14–4.10 (m, 2H, OCH_2_CH=CH_2_, H-5 Man), 4.01 (dd, *J* = 4.8, 10.5 Hz, 1H, H-6_a_ Glc_a_), 3.95 (app. t, *J* = 9.2 Hz, 1H, H-3 Glc_b_), 3.92 (app. t, *J* = 9.0 Hz, 1H, H-3 Glc_a_), 3.91–3.87 (m, 2H, H-4 Man, H-4 Glc_b_), 3.78 (app. t, *J* = 9.0 Hz, 1H, H-4 Glc_a_), 3.71 (dd, *J* = 2.0, 11.0 Hz, 1H, H-6_a_ Glc_b_), 3.66 (dd, *J* = 4.5, 10.9 Hz, 1H, H6_b_ Glc_a_), 3.64 (d, *J* = 3.0 Hz, 1H, H-2 Man), 3.52–3.43 (m, 4H, H-2 Glc_b_, H-5 Glc_a_, H-6_b_ Glc_a_), 3.24 (dd, *J* = 2.0, 11.3 Hz, 1H, H-6_a_ Man), 3.18 (d, *J* = 3.0, 9.6 Hz, 1H, H-3 Man), 3.05 (dd, *J* = 2.2, 11.2 Hz, 1H, H-6_b_ Man), 3.01 (td, *J* = 4.9, 9.7 Hz, 1H, H-5 Glc_b_), 2.2 (br s, 1H, OH). ^13^C NMR (150 MHz, CDCl_3_, δ_C_): 139.1, 138.5, 138.2, 138.1, 137.9, 137.6, 137.5, 137.3, 134.8, 128.8, 128.5, 128.4, 128.3, 128.2(9), 128.2(4), 128.1(9), 128.1(1), 128.0, 127.9, 127.8(4), 127.8(0), 127.7, 127.6, 127.5, 127.4, 127.33, 127.30, 126.9, 126.0, 116.9, 102.5 (C-1 Glc_a_), 101.4 (PhCH), 101.3 (C-1 Man), 97.4 (C-1, Glc_b_), 80.5, 80.1, 79.4, 78.9, 78.7, 78.1, 77.4, 77.0, 75.1, 74.7, 74.1, 73.9, 73.7, 73.6, 73.5, 71.5, 71.0, 69.6, 69.5, 68.6, 68.5, 67.9, 66.7. HRMS (ESI) calcd for [M + NH_4_]^+^ C_77_H_86_O_16_N: 1280.5969; found, 1280.5941.

#### Benzyl 2-*O*-acetyl-4,6-*O*-benzylidene-β-d-mannopyranosyl-(1→4)-2,3,6-tri-*O*-benzyl-α-d-glucopyranosyl-(1→3)-2,4,6-tri-*O*-benzyl-β-d-glucopyranoside (**20**)

To a solution of **19** (45 mg, 0.036 mmol) dissolved in pyridine (4.0 mL), cooled to 0 °C, acetic anhydride (8 μL, 0.08 mmol) was added followed by the addition of DMAP (1.6 mg, 0.007 mmol). The reaction mixture was gradually warmed to rt and stirred for 12 h and then concentrated. The residue was diluted with EtOAc (30 mL), and then the resulting organic solution was washed with 1 N HCl_(aq)_, saturated NaHCO_3(aq),_ H_2_O and brine. The organic layer was separated dried over MgSO_4_, filtered and concentrated. The crude residue obtained was dissolved in THF (2.0 mL) and then hydrogen-activated [Ir­(cod)­(Ph_2_MeP)_2_]­PF_6_ (2.7 mg, 0.0032 mmol, dissolved in 2 mL THF) was added. The solution was stirred at rt for 45 min and then the solvent was evaporated. After drying in vacuo for 1 h, the residue obtained was redissolved in acetone–water (4.0 mL, 10:1), followed by the addition of HgO (3.2 mg, 0.014 mmol) and HgCl_2_ (21 mg, 0.079 mmol). The mixture was stirred at rt for 2 h and then concentrated. The crude residue was purified by chromatography (EtOAc–Hexanes = 1:3) to afford **20** (372 mg, 0.69 mmol, 84% yield over three steps) as a white solid. *R*_*f*_ = 0.10, EtOAc–Hexanes = 1:3; ^1^H NMR (600 MHz, CDCl_3_, δ_H_): 7.47–7.46 (m, 2H, ArH), 7.39–7.27 (m, 26H, ArH), 7.22–7.20 (m, 5H, ArH), 7.15–7.14 (m, 1H, ArH), 7.12–7.09 (m, 2H, ArH),7.07–7.06 (m 2H, ArH), 7.01–7.00 (m, 2H, ArH), 5.51 (d, *J* = 3.8 Hz, 1H, H-1 Glc_b_), 5.46 (s, 1H, PhCH), 5.13 (d, *J* = 3.4 Hz, 1H, H-2 Man), 5.01 (d, *J* = 10.5 Hz, 1H, OCHPh), 4.96 (d, *J* = 11.7 Hz, 1H, OCHPh), 4.92 (d, *J* = 11.7 Hz, 1H, OCHPh), 4.72 (d, *J* = 11.7 Hz, 1H, OCHPh), 4.71 (d, *J* = 10.5 Hz, 1H, OCHPh), 4.67 (d, *J* = 11.8 Hz, 1H, OCHPh), 4.63 (d, *J* = 12.0 Hz, 1H, OCHPh), 4.61 (d, *J* = 12.0 Hz, 1H, OCHPh), 4.59 (d, *J* = 11.0 Hz, 1H, OCHPh), 4.53 (d, *J* = 7.8 Hz, 1H, H-1 Glc_a_), 4.52 (d, *J* = 12.0 Hz, 1H, OCHPh), 4.47 (d, *J* = 11.7 Hz, 1H, OCHPh), 4.40 (d, *J* = 11.7 Hz, 1H, OCHPh), 4.34 (s, 1H, H-1 Man), 4.14 (m, 2H, OCHPh, H-5 Glc_b_), 4.01 (dd, *J* = 4.9, 10.5 Hz, 1H, H-6_a_ Man), 3.94 (app. t, *J* = 10.0 Hz, 1H, H-4 Glc_b_), 3.90 (app. t, *J* = 9.0 Hz, 1H, H-3 Glc_a_), 3.88 (app. t, *J* = 9.2 Hz, 1H), 3.75 (app. t, *J* = 9.2 Hz, 1H, H-3 Glc_b_), 3.72–3.64 (m, 3H, H-6_b_ Man), 3.51–3.43 (m, 5H, H-2 Glc_b_, H-2 Glc_a_, H-3 Man), 3.17 (dd, *J* = 1.0, 11.0 Hz, 1H, H-6_a_ Glc_b_), 3.06 (dd, *J* = 1.7, 11.0 Hz, 1H, H-6_b_ Glc_b_), 3.01 (dt, *J* = 5.0, 9.7 Hz, 1H), 2.22 (br s, 1H, OH), 2.05 (s, 3H, CH_3_CO). ^13^C NMR (150 MHz, CDCl_3_, δ_C_): 170.4 (C=O), 139.3, 138.3, 138.1, 138.0, 137.6, 137.2, 137.2, 129.3, 128.6, 128.5, 128.4, 128.3(8), 128.3(0), 128.2(7), 128.2(0), 128.1, 127.9, 127.8, 127.7, 127.6(2), 127.6(0), 127.5, 127.3(6), 127.3(2), 127.2, 126.9, 126.2, 102.7 (C-1 Glc_a_), 102.1 (PhCH), 98.6 (C-1 Man), 97.8 (C-1, Glc_b_), 80.6, 79.9, 79.4, 78.7, 78.6, 74.9, 74.7, 74.0, 73.9, 73.6, 73.4, 71.3, 71.0, 69.8, 69.4, 68.7, 68.4, 67.6, 66.5, 20.8. HRMS (ESI) calcd for [M + Na]^+^ C_76_H_80_NaO_17_: 1287.5287; found, 1287.5287.

#### Benzyl 2,3,4-tri-*O*-benzyl-α-d-glucopyranosyluronate-(1→3)-4,6-*O*-benzylidene-β-d-mannopyranosyl-(1→4)-2,3,6-tri-*O*-benzyl-α-d-glucopyranosyl-(1→3)-2,4,6-tri-*O*-benzyl-β-d-glucopyranoside (**21**)

A mixture of acceptor **20** (40.0 mg, 0.031 mmol) and donor **15** (60 mg, 0.082 mmol) was coevaporated three times with anhydrous toluene (5.0 mL × 3) then dried in vacuo for 4 h. Activated pulverized 3 Å molecular sieves (200 mg) and CH_2_Cl_2_ (3.0 mL), Et_2_O (1.0 mL) were added and the mixture was stirred at rt for 30 min. The solution was cooled to −45 °C followed by slow addition TMSOTf (3.0 μL, 0.016 mmol). The reaction mixture was stirred at −45 °C for 30 min and then at −30 °C for 90 min before Et_3_N (50 μL) was added. The solution was filtered through a pad of Celite and the resulting solution was concentrated. The residue was dissolved in EtOAc (25.0 mL) and then washed with H_2_O and brine. The organic layer was dried over MgSO_4_, filtered and concentrated. The residue was then purified by chromatography to (EtOAc–Hexanes = 1:3, *R*_f_ = 0.10) to afford an α/β mixture of tetrasaccharide (46 mg, 0.027 mmol, 88% yield). To a solution of this tetrasaccharide (46.0 mg, 0.027) in CH_3_OH (6.8 mL), was added 1 M NaOH (1.2 mL) and the solution was allowed to stir at rt. After 12 h, then solution was concentrated and the residue was dissolved in EtOAc (30.0 mL) and then washed with H_2_O and brine. The organic layer was separated, dried over MgSO_4_, filtered and concentrated. Purification by chromatography (EtOAc–Hexanes = 1:1) afforded **21** (27 mg, 0.016 mmol, 59% yield) as a white solid. *R*_f_ = 0.20, EtOAc–Hexanes = 1:1; ^1^H NMR (600 MHz, CDCl_3_, δ_H_): 7.37–7.36 (m, 2H, ArH), 7.33–7.26 (m, 35H, ArH), 7.22–7.18 (m, 6H, ArH), 7.16–7.08 (m, 8H, ArH), 7.04–7.02 (m, 2H, ArH),6.98–6.97 (m 2H, ArH), 5.53 (d, *J* = 3.7 Hz, 1H, H-1 Glc_b_), 5.37 (s, 1H, PhCH), 5.30 (d, *J* = 3.6 Hz, 1H, H-1 GlcA), 5.04 (d, *J* = 11.0 Hz, 1H, OCHPh), 4.98 (d, *J* = 11.0 Hz, 1H, OCHPh), 4.95 (d, *J* = 12.0 Hz, 1H, OCHPh), 4.92 (d, *J* = 11.0 Hz, 1H, OCHPh), 4.90 (d, *J* = 11.7 Hz, 1H, OCHPh), 4.89 (d, *J* = 10.7 Hz, 1H, OCHPh), 4.79 (d, *J* = 10.9 Hz, 1H, OCHPh), 4.78 (d, *J* = 10.7 Hz, 1H, OCHPh), 4.71 (d, *J* = 11.0 Hz, 1H, OCHPh), 4.69 (d, *J* = 11.8 Hz, 1H, OCHPh), 4.66 (d, *J* = 12.0 Hz, 1H, OCHPh), 4.63–4.61 (m, 2H, OCHPh), 4.57–4.51 (m, 4H, OCHPh), 4.51 (d, *J* = 7.7 Hz, 1H, H-1 Glc_a_), 4.45 (d, *J* = 11.5 Hz, 1H, OCHPh), 4.43 (d, *J* = 10.2 Hz, 1H, OCHPh), 4.36 (d, *J* = 12.2 Hz, 1H, OCHPh), 4.26 (s, 1H, H-1 Man), 4.16 (d, *J* = 12.2 Hz, 1H, OCHPh), 4.13–4.12 (m, 1H), 4.09 (app. t, *J* = 9.3 Hz, 1H), 4.01 (dd, *J* = 4.9, Hz, 10.5 Hz, 1H, H-6_a_ Man), 3.98 (app. t, *J* = 9.6 Hz, 1H, H-4 Man), 3.94–3.87 (m, 3H), 3.78 (app. t, *J* = 9.2 Hz, 1H), 3.74–3.69 (m, 3H), 3.67 (dd, *J* = 4.5, 11.0 Hz, 1H), 3.52–3.43 (m, 6H, H-2GlcA, H-2 Glc_b_, H-2 Glc_a,_, H-2 Man, H-6_b_ Man), 3.28 (dd, *J* = 1.7, 11.2 Hz, 1H), 3.01 (dd, *J* = 2.0, 11.4 Hz, 1H), 2.97 (td, *J* = 5.0, 9.8 Hz, 1H, H-5 Man). ^13^C NMR (150 MHz, CDCl_3_, δ_C_): 170.0 (C=O), 139.1, 138.4, 138.2, 138.1, 137.9, 137.7, 137.6, 137.5, 137.3, 129.3, 128.6, 128.5, 128.4, 128.3, 128.2, 128.1(9), 128.1(6), 128.0(9), 128.0(1), 127.8(6), 127.8(2), 127.7(9), 127.7(6), 127.7(0), 127.6, 127.5, 127.4, 127.3, 126.9, 126.3, 102.5 (C-1 Glc_a_), 102.1 (PhCH), 99.7 (C-1 Man), 97.3 (C-1 GlcA, C-1 Glc_b_), 80.6, 80.3, 80.0, 79.0, 78.9, 78.8, 78.7, 78.3, 77.7, 75.9, 75.7, 75.4, 75.1, 74.7, 74.2, 73.9, 73.6, 73.5, 73.4, 71.3, 71.0, 70.9, 69.6, 69.4, 68.6, 68.5, 67.9, 66.6. HRMS (ESI) calcd for [M + NH_4_]^+^ C_101_H_108_O_22_N: 1686.7358; found, 1686.7406.

## Supplementary Material



## Notes

^1^H and ^13^C NMR spectra of previously unreported compounds (compounds **1**–**5**, **9**–**12**, **14**–**16** and **18**–**21**) (PDF)

## References

[1] Bengoechea J. A., Sa Pessoa J. (2019). *Klebsiella pneumoniae* infection biology:
living to counteract host defences. FEMS Microbiol.
Rev..

[2] Pan Y.-J., Lin T.-L., Chen C.-T., Chen Y.-Y., Hsieh P.-F., Hsu C.-R., Wu M.-C., Wang J.-T. (2015). Genetic analysis
of capsular polysaccharide synthesis gene clusters in 79 capsular
types of Klebsiella spp. Sci. Rep..

[3] Miller W. R., Arias C. A. (2024). ESKAPE pathogens: antimicrobial resistance, epidemiology,
clinical impact and therapeutics. Nat. Rev.
Microbiol..

[4] Tacconelli Evelina . Global priority list of antibiotic-resistant bacteria to guide research, discovery, and development; Infection Control Africa Network: South Africa, 2017.

[5] Latka A., Lemire S., Grimon D., Dams D., Maciejewska B., Lu T., Drulis-Kawa Z., Briers Y. (2021). Engineering the Modular Receptor-Binding
Proteins of Klebsiella Phages Switches Their Capsule Serotype Specificity. mBio.

[6] Dunstan R. A., Bamert R. S., Tan K. S., Imbulgoda U., Barlow C. K., Taiaroa G., Pickard D. J., Schittenhelm R. B., Dougan G., Short F. L. (2023). Epitopes
in the capsular
polysaccharide and the porin OmpK36 receptors are required for bacteriophage
infection of *Klebsiella pneumoniae*. Cell Rep..

[7] Siu L. K., Tsai Y.-K., Lin J.-C., Chen T.-L., Fung C.-P., Chang F.-Y. (2016). Development of a
Colloidal Gold-Based Immunochromatographic
Strip for Rapid Detection of *Klebsiella pneumoniae* Serotypes K1 and K2. J. Clin. Microbiol..

[8] Wantuch P. L., Knoot C. J., Robinson L. S., Vinogradov E., Scott N. E., Harding C. M., Rosen D. A. (2023). Capsular polysaccharide
inhibits vaccine-induced O-antigen antibody binding and function across
both classical and hypervirulent K2: O1 strains of *Klebsiella
pneumoniae*. PLoS Pathog..

[9] Lin T.-L., Yang F.-L., Ren C.-T., Pan Y.-J., Liao K.-S., Tu I.-F., Chang Y.-P., Cheng Y.-Y., Wu C.-Y., Wu S.-H. (2022). Development
of *Klebsiella pneumoniae* capsule polysaccharide-conjugated
vaccine candidates using phage
depolymerases. Front. Immunol..

[10] Geyer H., Stirm S., Himmelspach K. (1979). Immunochemical
properties of oligosaccharide-protein
conjugates with Klebsiella-K2 specificity: I. Specificity and crossreactivity
of anti-conjugate versus anti-bacterial antibodies. Med. Microbiol. Immunol..

[11] Ye T.-J., Fung K.-M., Lee I.-M., Ko T.-P., Lin C.-Y., Wong C.-L., Tu I.-F., Huang T.-Y., Yang F.-L., Chang Y.-P. (2024). *Klebsiella pneumoniae* K2 capsular
polysaccharide degradation by a bacteriophage depolymerase does not
require trimer formation. mBio.

[12] Tu I.-F., Lin T.-L., Yang F.-L., Lee I.-M., Tu W.-L., Liao J.-H., Ko T.-P., Wu W.-J., Jan J.-T., Ho M.-R. (2022). Structural and biological insights into *Klebsiella
pneumoniae* surface polysaccharide degradation by a bacteriophage
K1 lyase: implications for clinical use. J.
Biomed. Sci..

[13] Oberli M. A., Tamborrini M., Tsai Y.-H., Werz D. B., Horlacher T., Adibekian A., Gauss D., Möller H. M., Pluschke G., Seeberger P. H. (2010). Molecular analysis of carbohydrate–
antibody interactions: case study using a *Βacillus anthra* tetrasaccharide. J. Am. Chem. Soc..

[14] Tan Z., Yang W., O’Brien N. A., Pan X., Ramadan S., Marsh T., Hammer N., Cywes-Bentley C., Vinacur M., Pier G. B. (2024). A comprehensive synthetic
library of poly-N-acetyl glucosamines enabled vaccine against lethal
challenges of *Staphylococcus aureus*. Nat. Commun..

[15] Misra A. K., Roy N. (1995). Synthesis of the tetrasaccharide
repeating unit of the antigen from
Klebsiella type 2. Carbohydr. Res..

[16] Ravinder M., Liao K.-S., Cheng Y.-Y., Pawar S., Lin T.-L., Wang J.-T., Wu C.-Y. (2020). A synthetic
carbohydrate–protein
conjugate vaccine candidate against *Klebsiella pneumoniae* serotype K2. J. Org. Chem..

[17] Crich D., Sun S. (1998). Direct formation of β-mannopyranosides
and other hindered glycosides
from thioglycosides. J. Am. Chem. Soc..

[18] Kochetkov N., Dmitriev B., Malysheva N., Chernyak A. Y., Klimov E., Bayramova N., Torgov V. (1975). Synthesis of O-β-D-mannopyranosyl-(1→4)-O-α-L-rhamnopyranosyl-(1→3)-D-galactopyranose,
the trisaccharide repeating-unit of the O-specific polysaccharide
from Salmonella anatum. Carbohydr. Res..

[19] Alais J., David S. (1990). Preparation of disaccharides
having a β-D-mannopyranosyl group
from N-phthaloyllactosamine derivatives by double or triple SN2 substitution. Carbohydr. Res..

[20] Chatterjee S., Moon S., Hentschel F., Gilmore K., Seeberger P. H. (2018). An empirical
understanding of the glycosylation reaction. J. Am. Chem. Soc..

[21] Paulsen H., Adermann K. (1988). Synthese von 8-methoxycarbonyloctyl-2-acetamido-2-desoxy-3-*O*-α-D-galactopyranosyl-α-D-galactopyranoside. Carbohydr. Res..

[22] Hsu Y., Lu X.-A., Zulueta M. M. L., Tsai C.-M., Lin K.-I., Hung S.-C., Wong C.-H. (2012). Acyl and
silyl group effects in reactivity-based
one-pot glycosylation: synthesis of embryonic stem cell surface carbohydrates
Lc4 and IV2Fuc-Lc4. J. Am. Chem. Soc..

[23] Lu S.-R., Lai Y.-H., Chen J.-H., Liu C.-Y., Mong K.-K. T. (2011). Dimethylformamide:
an unusual glycosylation modulator. Angew. Chem.,
Int. Ed..

[24] Kafle A., Liu J., Cui L. (2016). Controlling
the stereoselectivity of glycosylation
via solvent effects. Can. J. Chem..

[25] Fontana C., Widmalm G. (2023). Primary structure of glycans by NMR spectroscopy. Chem. Rev..

[26] Codée J. D., Christina A. E., Walvoort M. T., Overkleeft H. S., Van der Marel G. A. (2010). Uronic
acids in oligosaccharide and glycoconjugate
synthesis. React. Tuning Oligosaccharide Assem..

[27] Mukherjee M. M., Basu N., Nandi S., Ghosh R. (2019). A metal free
mild and
green approach for tandem opening of 4,6-O-benzylidene acetals to
their corresponding 6-O-acetyl derivatives: Application in the synthesis
of a trisaccharide using one-pot glycosylation reactions. Carbohydr. Res..

[28] Yin S., Li L., Su L., Li H., Zhao Y., Wu Y., Liu R., Zou F., Ni G. (2022). Synthesis and in vitro synergistic
antifungal activity of analogues of *Panax stipulcanatus* saponin against fluconazole-resistant Candida albicans. Carbohydr. Res..

[29] Lim, Y. J. Studies Undertaken Towards the Total Synthesis of Antifreeze Compounds Based on the Xylomannan Antifreeze from the Alaskan Beetle Upis ceramboides. Ph.D. Thesis, University of Alberta, Canada, 2019.

