# Supplementary material for: Synthesis of the Tetrasaccharide Repeating Unit from Klebsiella pneumoniae Serotype K2 Capsular Polysaccharide
Source: ACS Omega. 2025 Jun 23;10(26):28382–94. doi: 10.1021/acsomega.5c03675 (PMC12242650; doi:10.1021/acsomega.5c03675)
Supplement: Supplementary file 1 [file ao5c03675_si_001.pdf]

Synthesis of the Tetrasaccharide Repeating Unit from *Klebsiella pneumoniae* Serotype K2 Capsular Polysaccharide

Mohammad Tarique Anwar,[1] Todd L. Lowary*[,1,2]

1. Institute of Biological Chemistry, Academia Sinica, Academia Road Section 2, #128, Nangang, Taipei, 115 Taiwan

2. Institute of Biochemical Sciences, National Taiwan University, Taipei 106 Taiwan

Email: tlowary@as.edu.tw

Supporting Information

Table of Contents

Figure S1. 1H NMR spectra of compound **1** **S1**

Figure S2. 13C NMR spectra of compound **1** **S2**

Figure S3. 2D COSY NMR spectra of compound **1** **S3**

Figure S4. 2D HSQC NMR spectra of compound **1** **S4**

Figure S5. 2D Coupled HSQC NMR spectra of compound **1** **S5**

Figure S6 1H NMR spectra of compound **2** **S6**

Figure S7. 13C NMR spectra of compound **2** **S7**

Figure S8. 2D COSY NMR spectra of compound **2** **S8**

Figure S9. 2D HSQC NMR spectra of compound **2** **S9**

Figure S10. Coupled HSQC NMR spectra of compound **2** **S10**

Figure S11. 1H NMR spectra of compound **3** **S11**

Figure S12. 13C NMR spectra of compound **3** **S12**

Figure S13. 2D COSY NMR spectra of compound **3** **S13**

Figure S14. 2D HSQC NMR spectra of compound **3** **S14**

Figure S15. 1H NMR spectra of compound **4** **S15**

Figure S16. 13C NMR spectra of compound **4** **S16**

Figure S17. 2D COSY NMR spectra of compound **4** **S17**

Figure S18. 2D HSQC NMR spectra of compound **4** **S18**

Figure S19. 1H NMR spectra of compound **5** **S19**

Figure S20. 13C NMR spectra of compound **5** **S20**

Figure S21. 2D COSY NMR spectra of compound **5** **S21**

Figure S22. 2D HSQC NMR spectra of compound **5** **S22**

Figure S23. 1H NMR spectra of compound **9** **S23**

Figure S24. 13C NMR spectra of compound **9** **S24**

Figure S25. 2D COSY NMR spectra of compound **9** **S25**

Figure S26. 2D HSQC NMR spectra of compound **9** **S26**

Figure S27. 1H NMR spectra of compound **10** **S27**

Figure S28. 13C NMR spectra of compound **10** **S28**

Figure S29. 1H NMR spectra of compound **11** **S29**

Figure S30. 13C NMR spectra of compound **11** **S30**

Figure S31. 1H NMR spectra of compound **12** **S31**

Figure S32. 13C NMR spectra of compound **12** **S32**

Figure S33. 1H NMR spectra of compound **14** **S33**

Figure S34. 13C NMR spectra of compound **14** **S34**

Figure S35. 1H NMR spectra of compound **15** **S35**

Figure S36. 13C NMR spectra of compound **15** **S36**

Figure S37. 1H NMR spectra of compound **16** **S37**

Figure S38. 13C NMR spectra of compound **16** **S38**

Figure S39. 2D COSY NMR spectra of compound **16** **S39**

Figure S40. 2D HSQC NMR spectra of compound **16** **S40**

Figure S41. 1H NMR spectra of compound **18** **S41**

Figure S42. 13C NMR spectra of compound **18** **S42**

Figure S43. 2D COSY NMR spectra of compound **18** **S43**

Figure S44. 2D HSQC NMR spectra of compound **18** **S44**

Figure S45. 2D Coupled HSQC NMR spectra of compound **18** **S45**

Figure S46. 1H NMR spectra of compound **19** **S46**

Figure S47. 13C NMR spectra of compound **19** **S47**

Figure S48. 2D COSY NMR spectra of compound **19** **S48**

Figure S49. 2D HSQC NMR spectra of compound **19** **S49**

Figure S50. 1H NMR spectra of compound **20** **S50**

Figure S51. 13C NMR spectra of compound **20** **S51**

Figure S52. 2D COSY NMR spectra of compound **20** **S52**

Figure S53. 2D HSQC NMR spectra of compound **20** **S53**

Figure S54. 2D Coupled HSQC NMR spectra of compound **20** **S54**

Figure S55. 1H NMR spectra of compound **21** **S55**

Figure S56. 13C NMR spectra of compound **21** **S56**

Figure S57. 2D COSY NMR spectra of compound **21** **S57**

Figure S58. 2D HSQC NMR spectra of compound **21** **S58**

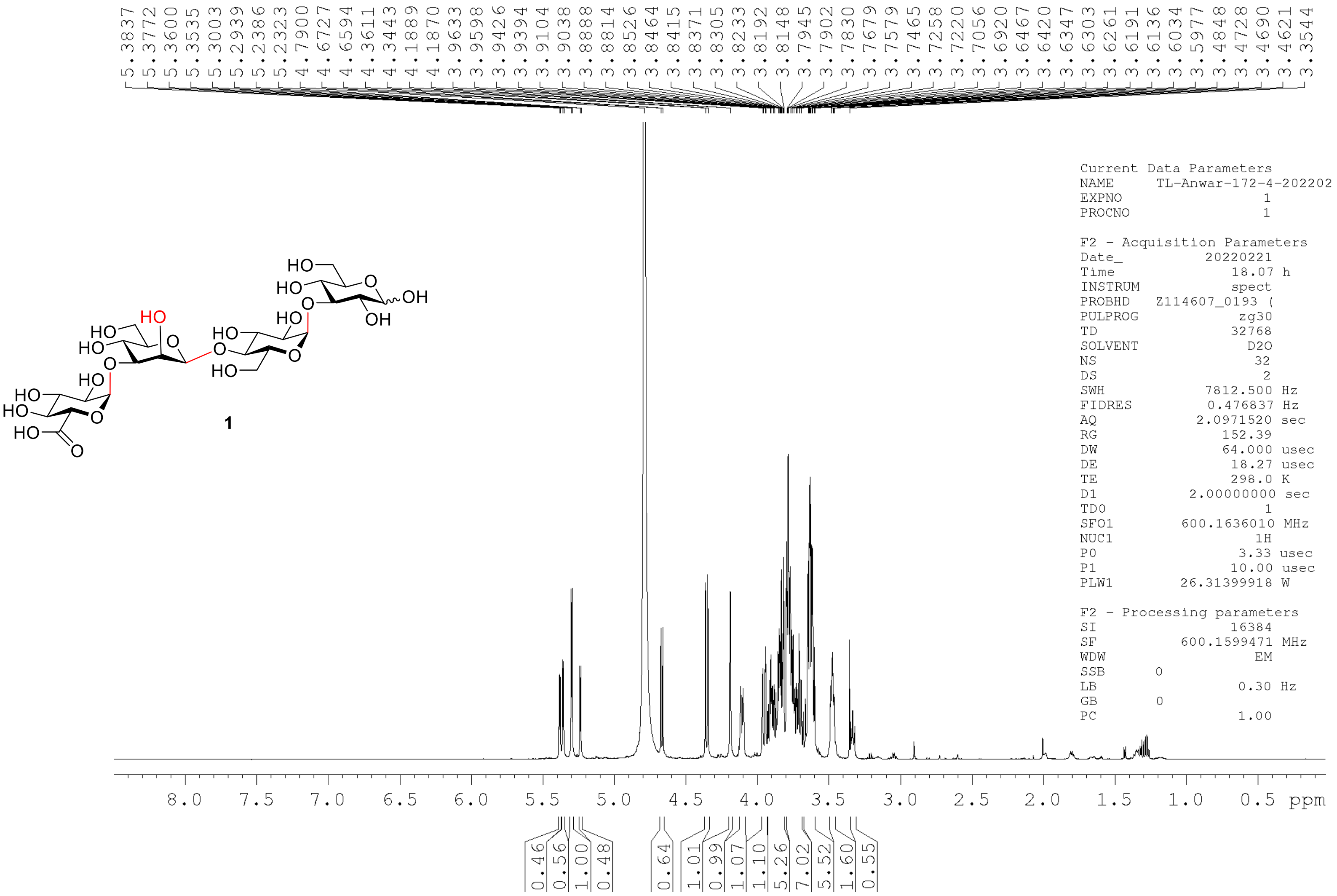

Figure S1. ^{1}H NMR Spectrum of compound **1**

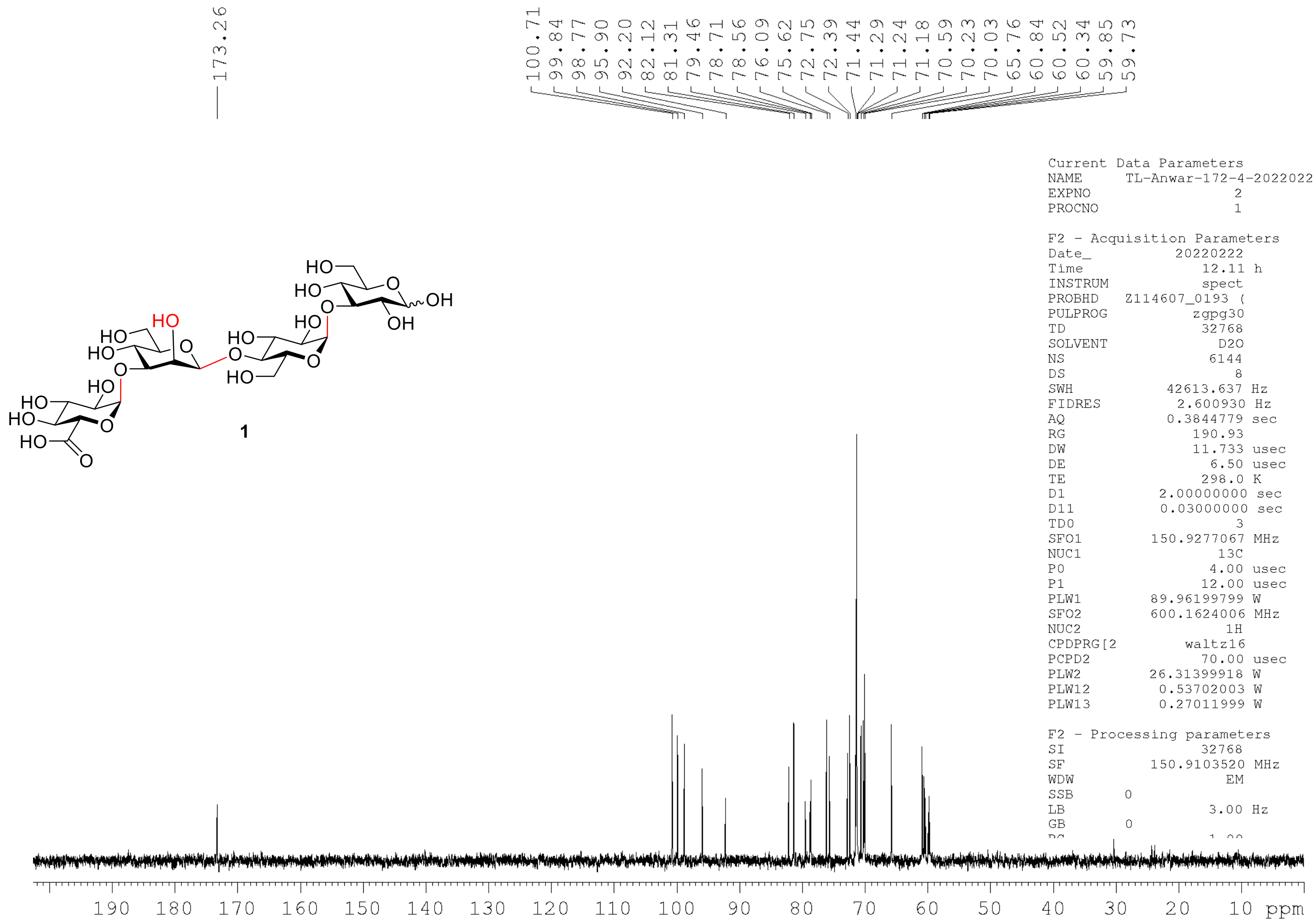

Figure S2. ^{13}C NMR Spectrum of compound **1**

2D-COSY

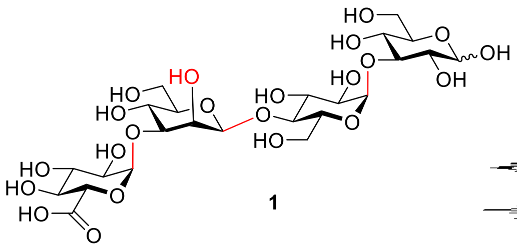

1

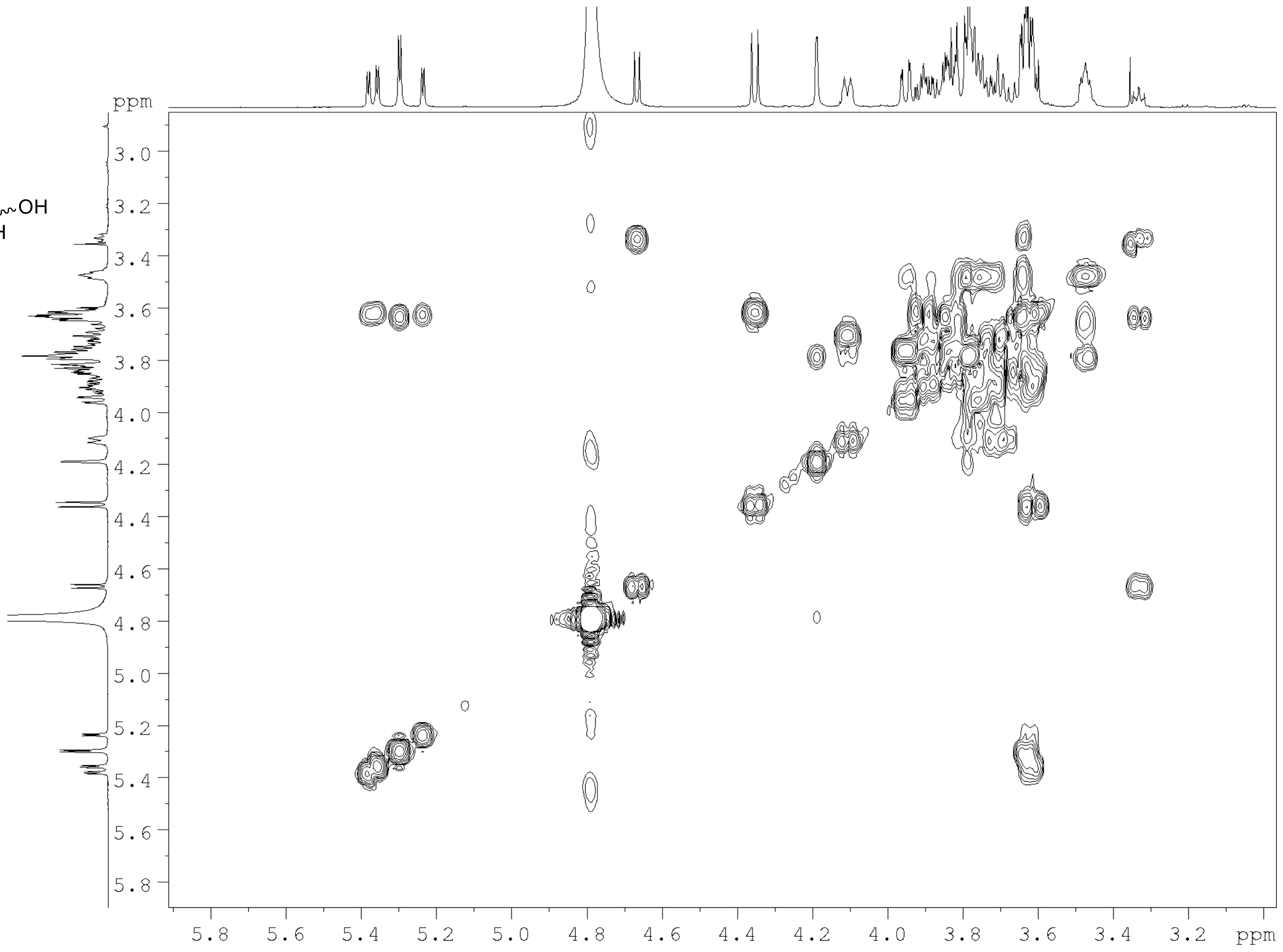

Figure S3. 2D-COSY NMR Spectrum of compound **1**

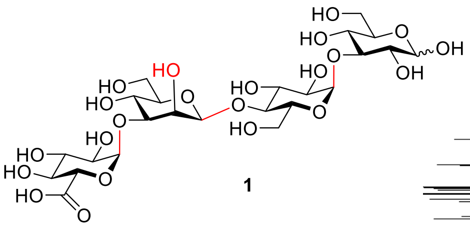

1

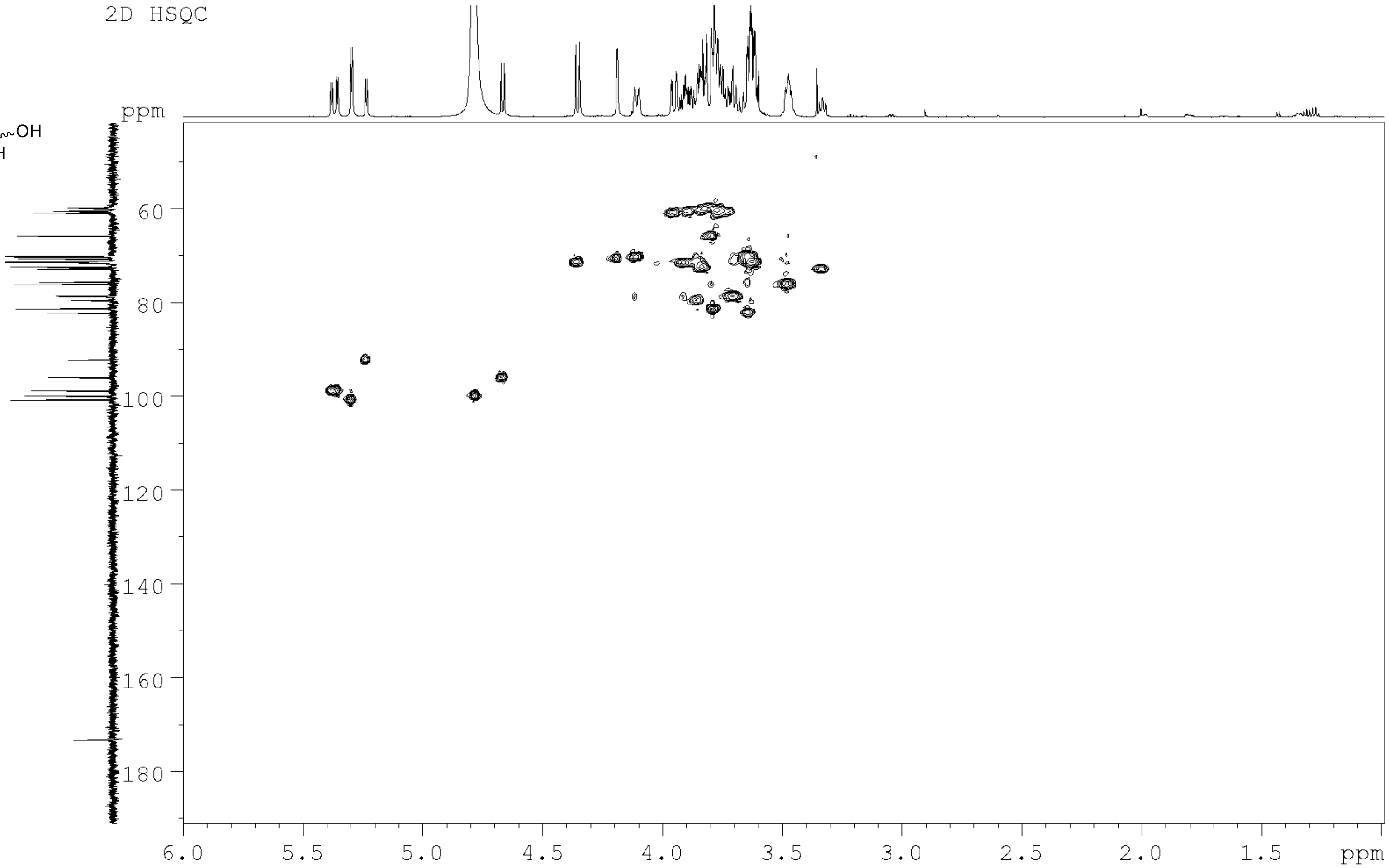

Figure S4. 2D-HSQC NMR Spectrum of compound **1**

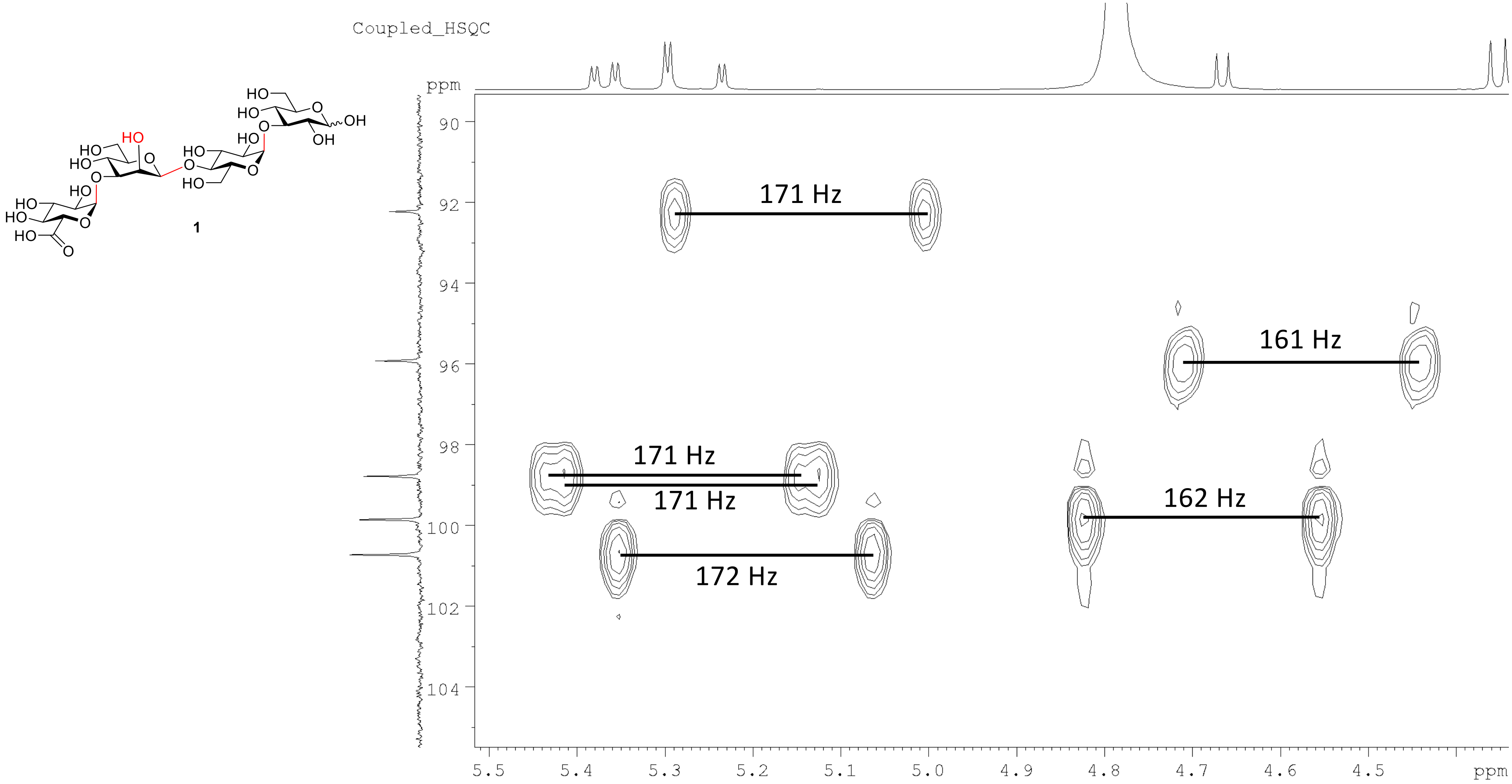

Figure S5. 2D-Copled HSQC NMR Spectrum of compound **1**

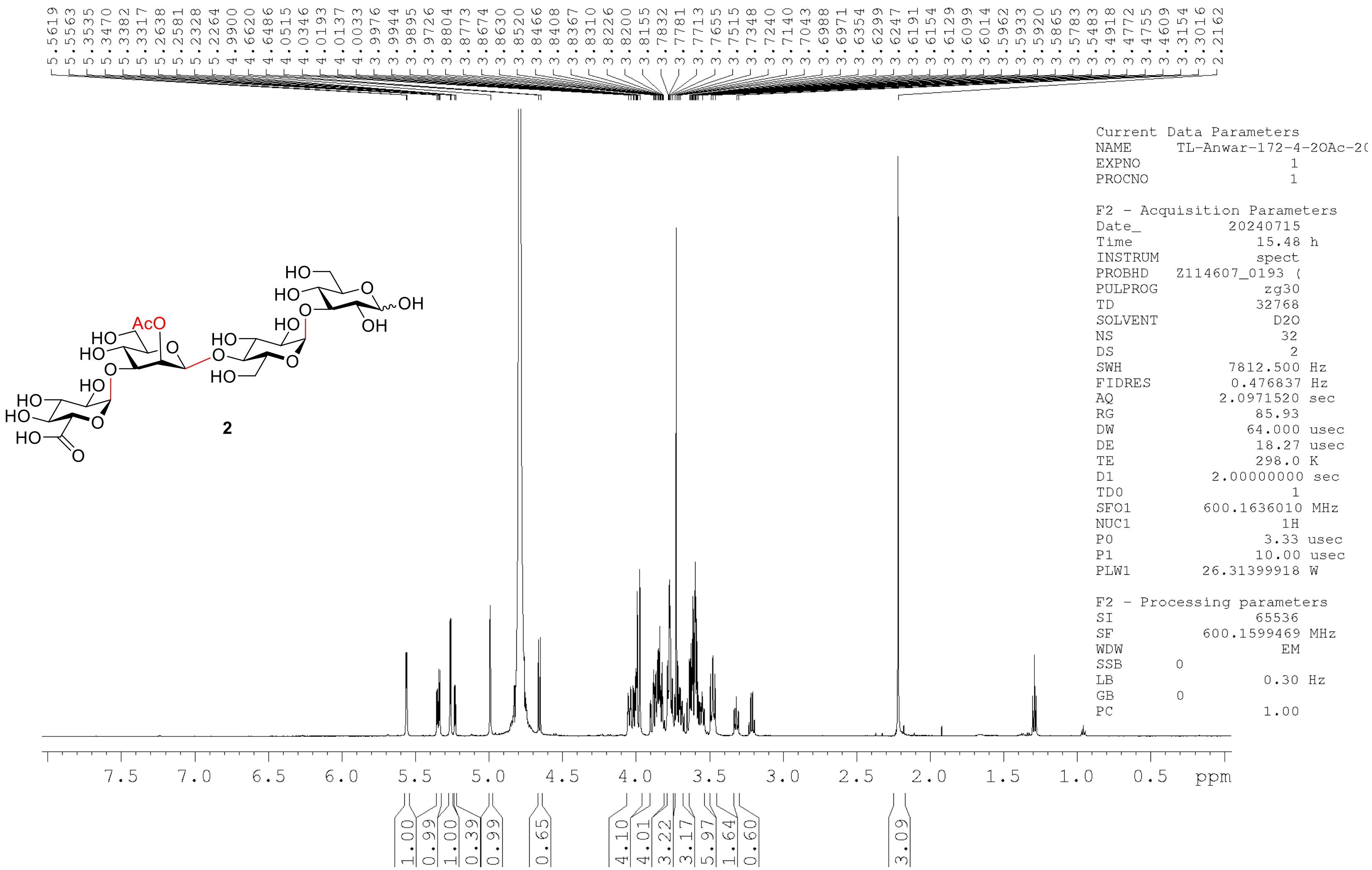

Figure S6. ^{1}H NMR Spectrum of compound **2**

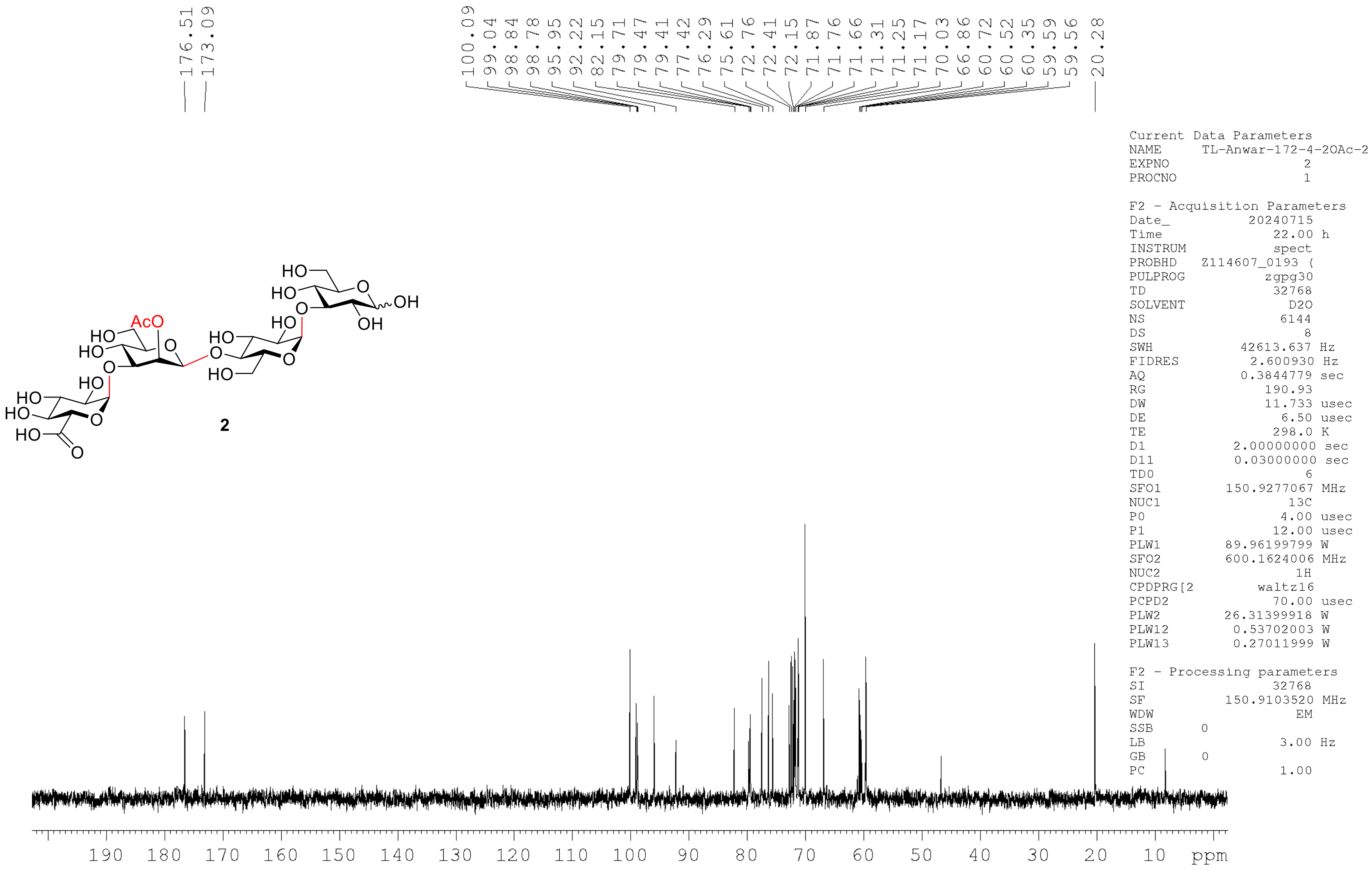

Figure S7. ^{13}C NMR Spectrum of compound **2**

2D-COSY

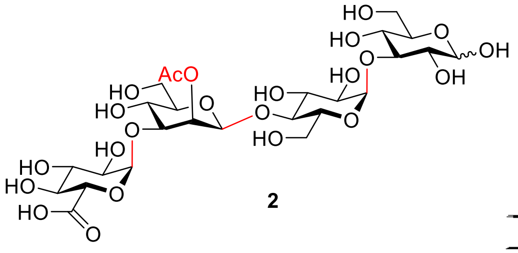

2

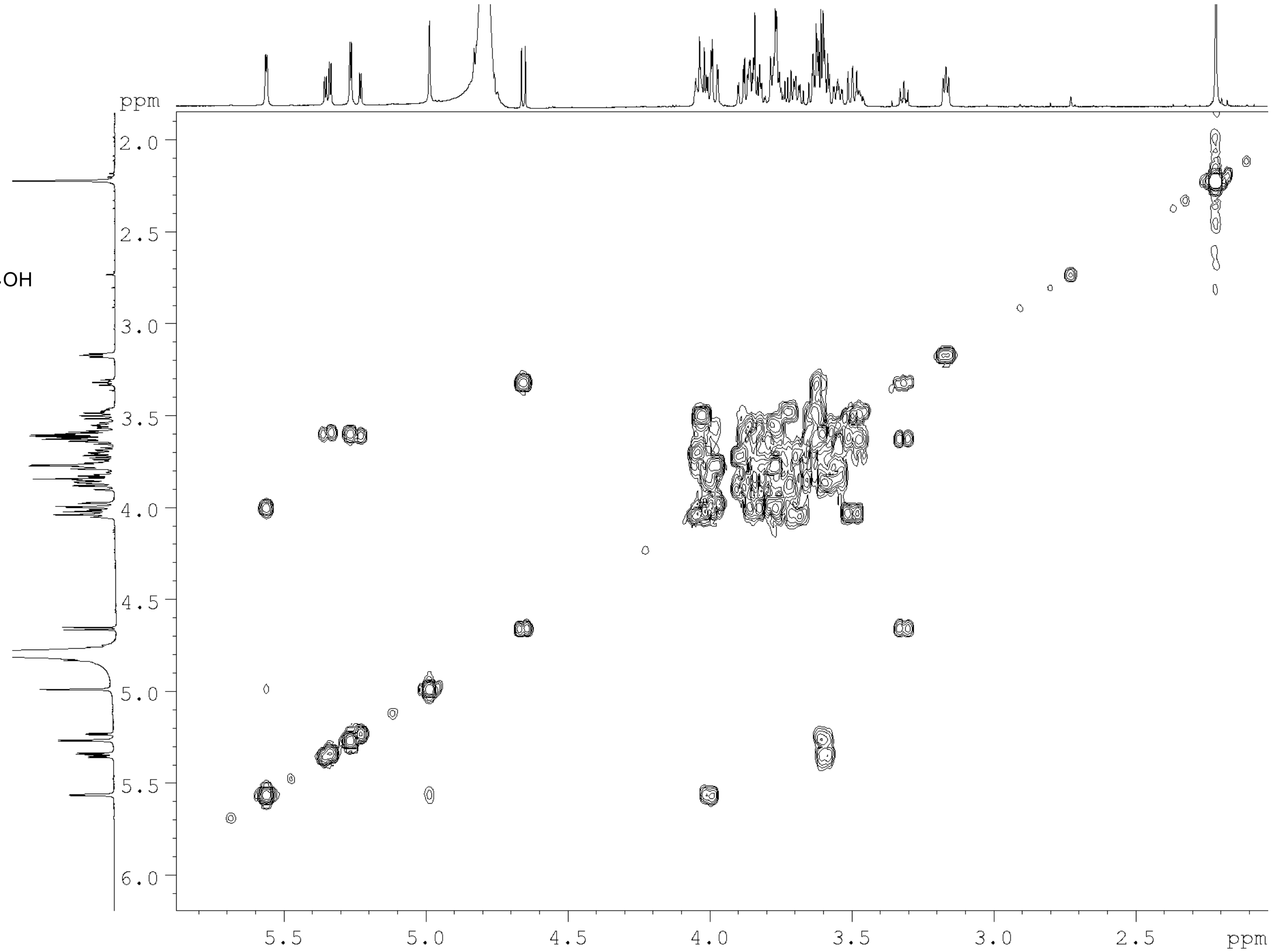

Figure S8. 2D COSY NMR Spectrum of compound **2**

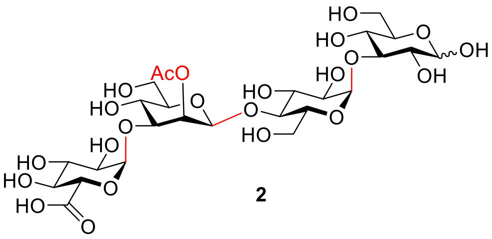

2

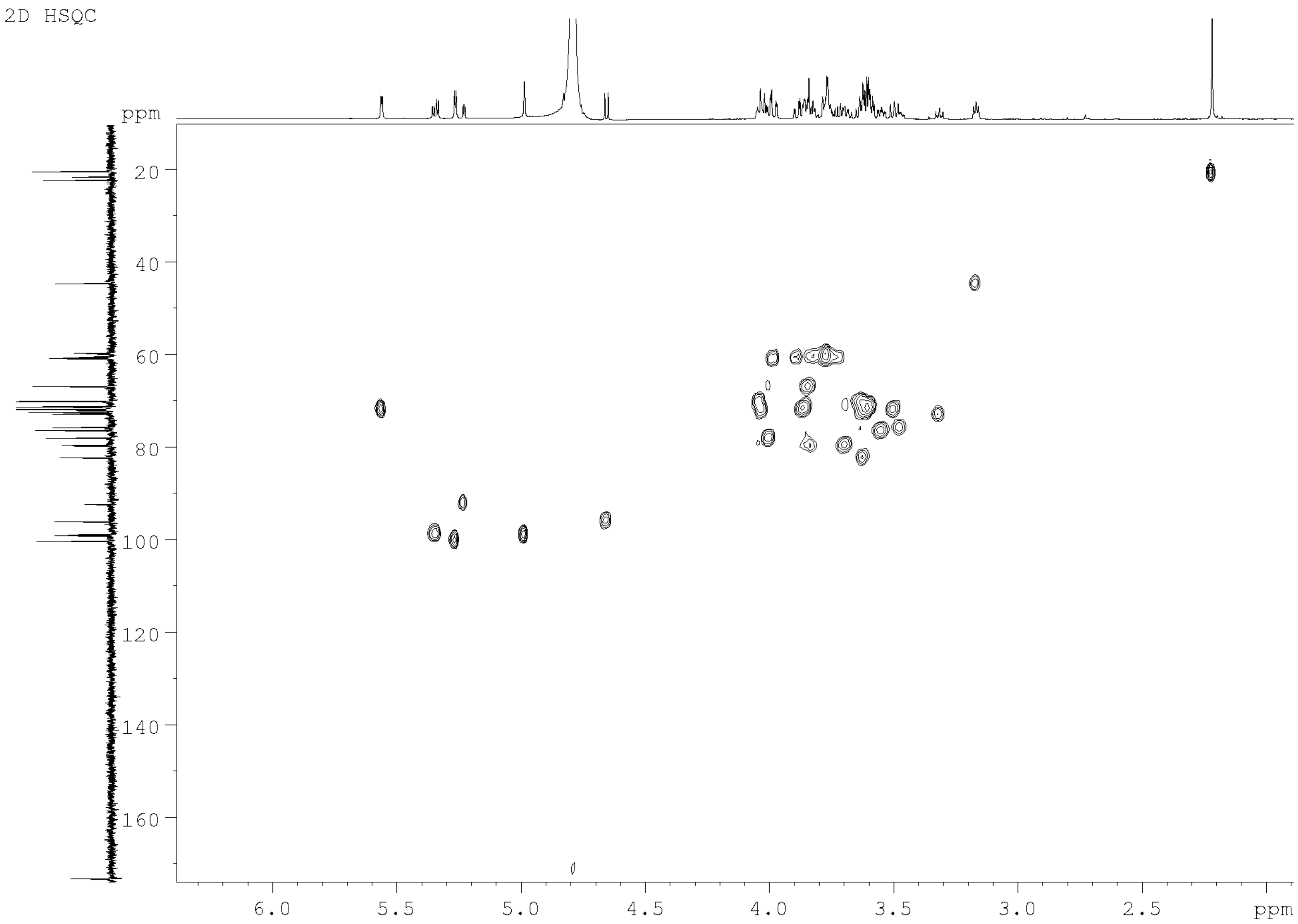

Figure S9. 2D HSQC NMR Spectrum of compound **2**

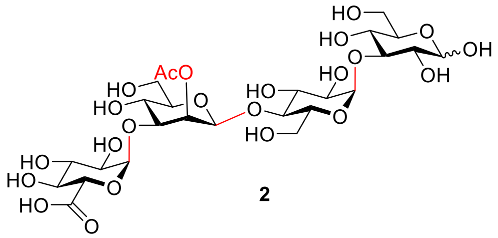

2

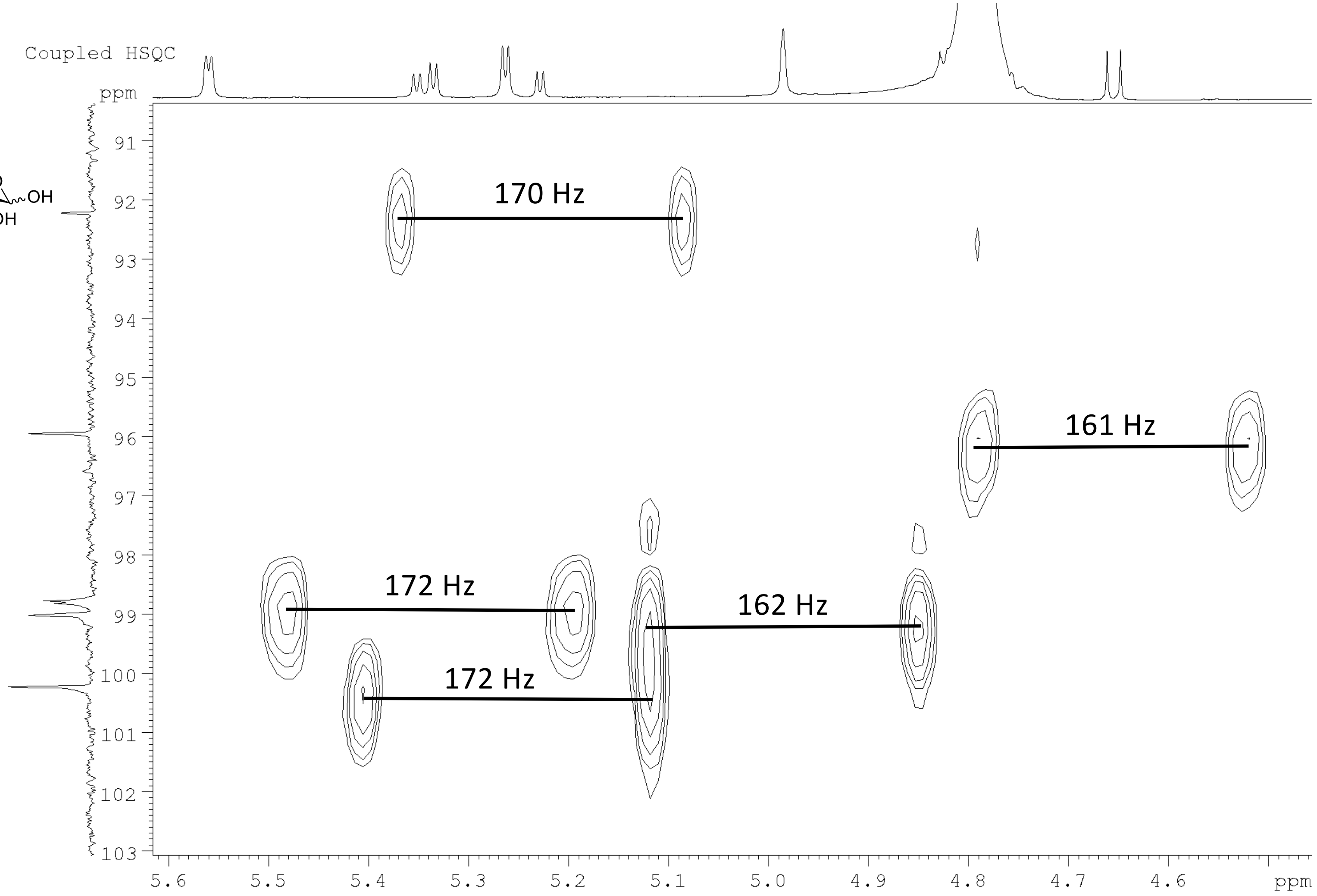

Figure S10. 2D Coupled HSQC NMR Spectrum of compound **2**

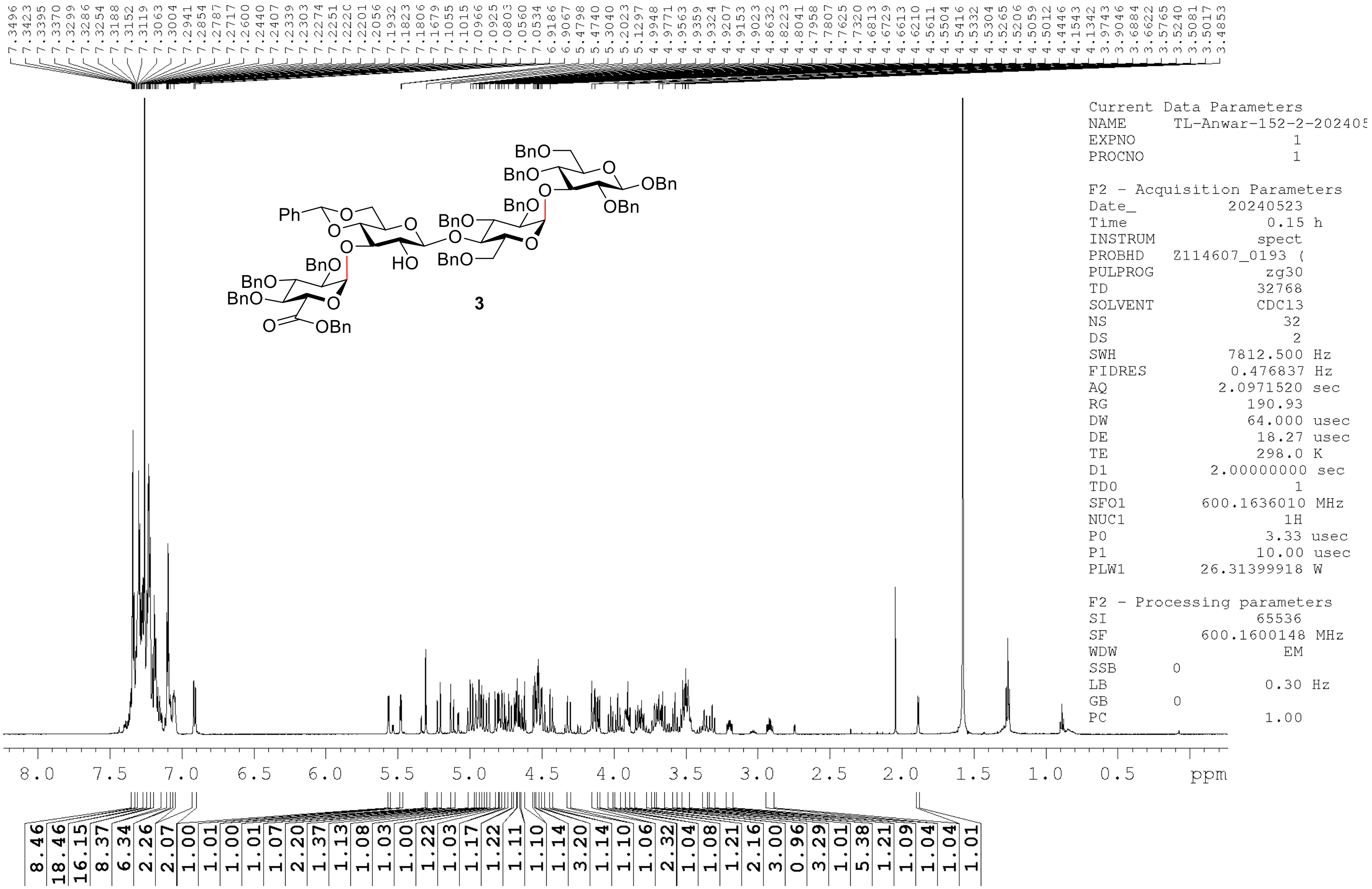

Figure S11. ^{1}H NMR Spectrum of compound **3**

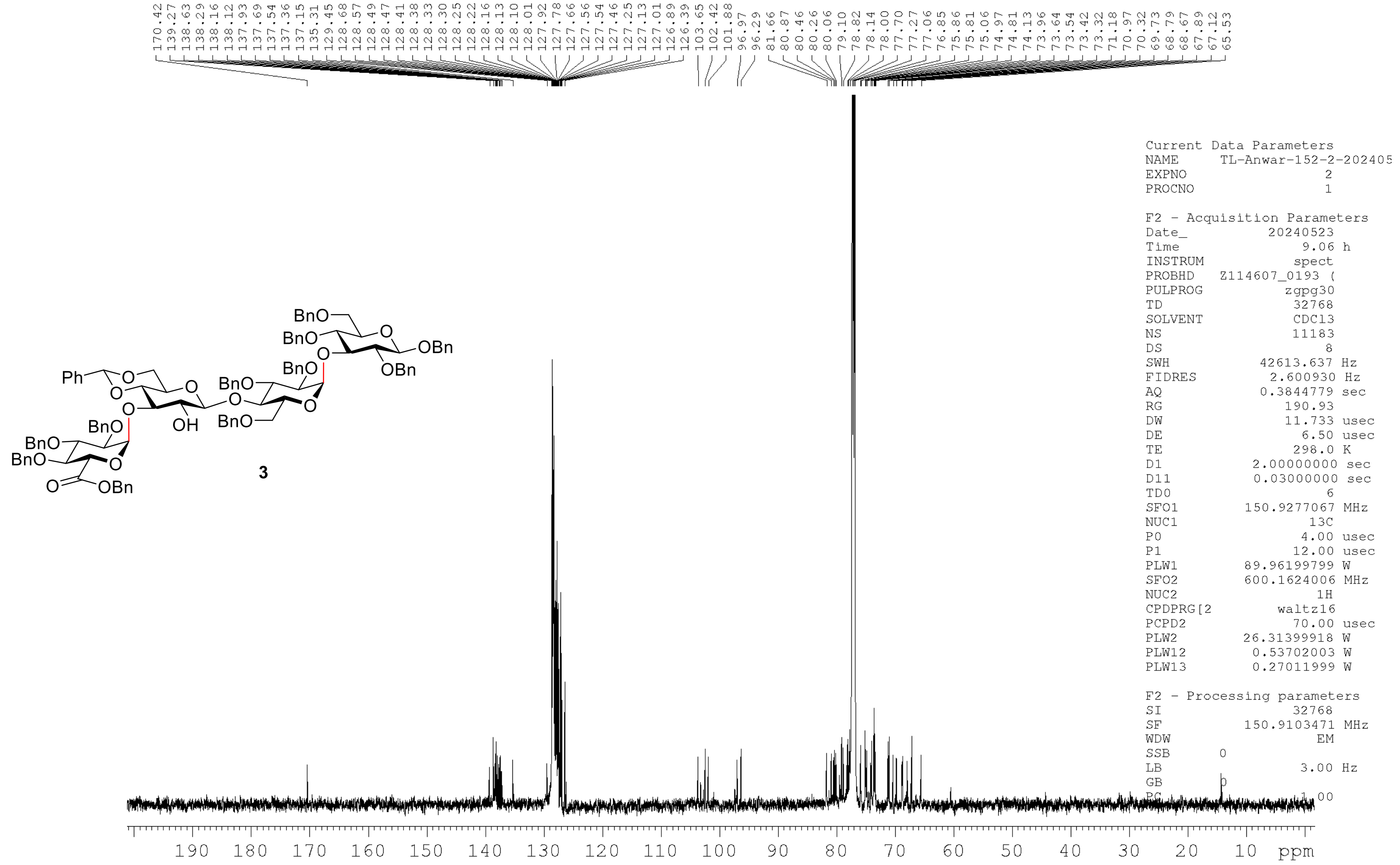

Figure S12. ^{13}C NMR Spectrum of compound **3**

2D-COSY

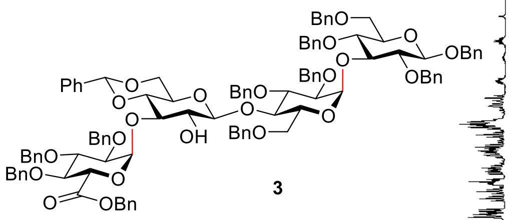

3

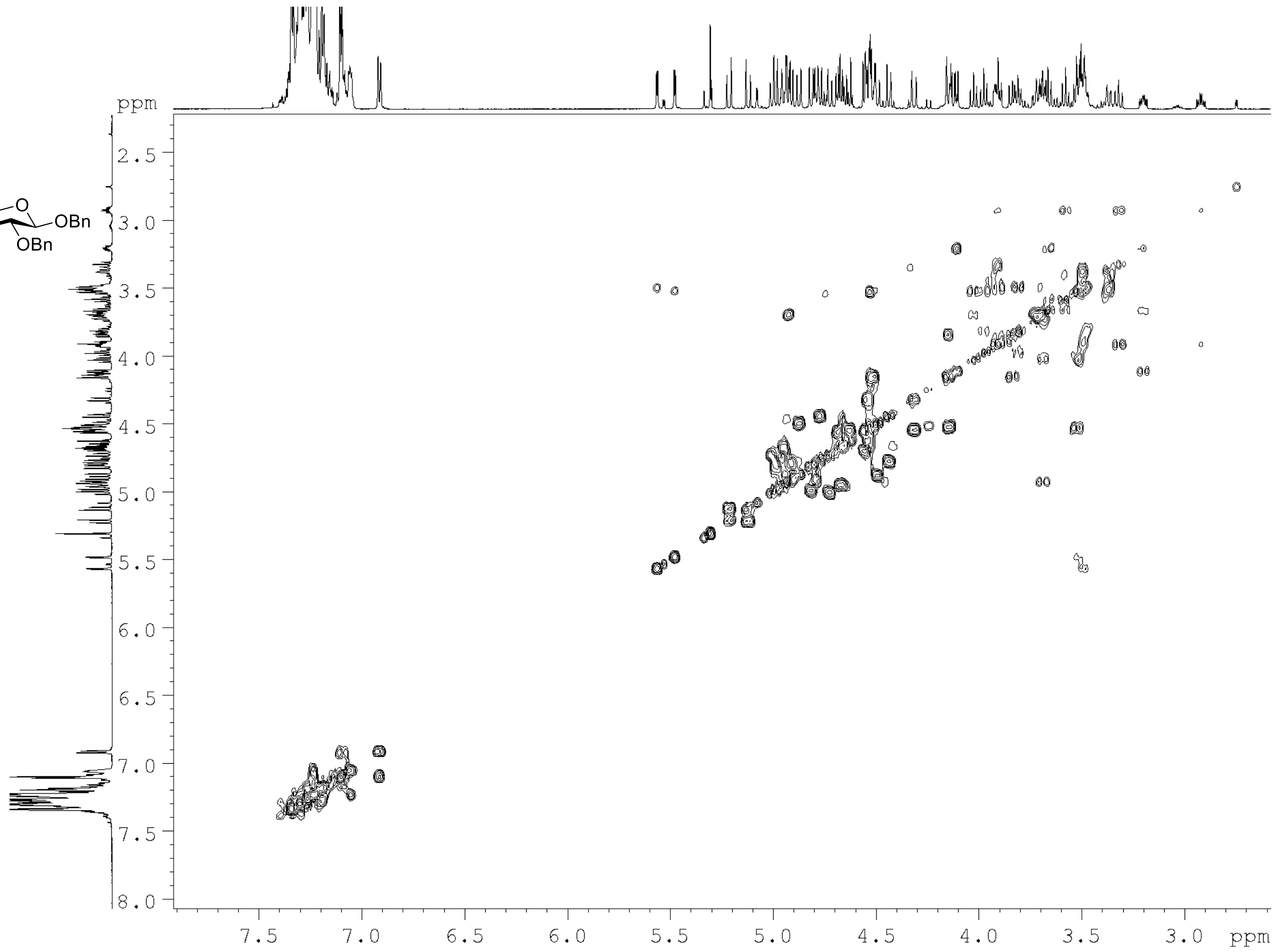

Figure S13. 2D COSY NMR Spectrum of compound **3**

2D-HSQC

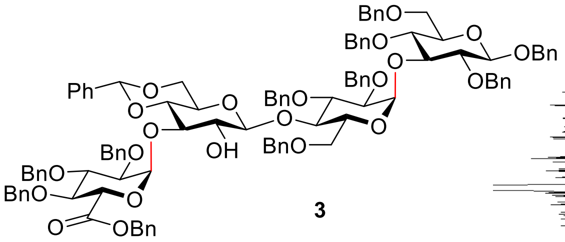

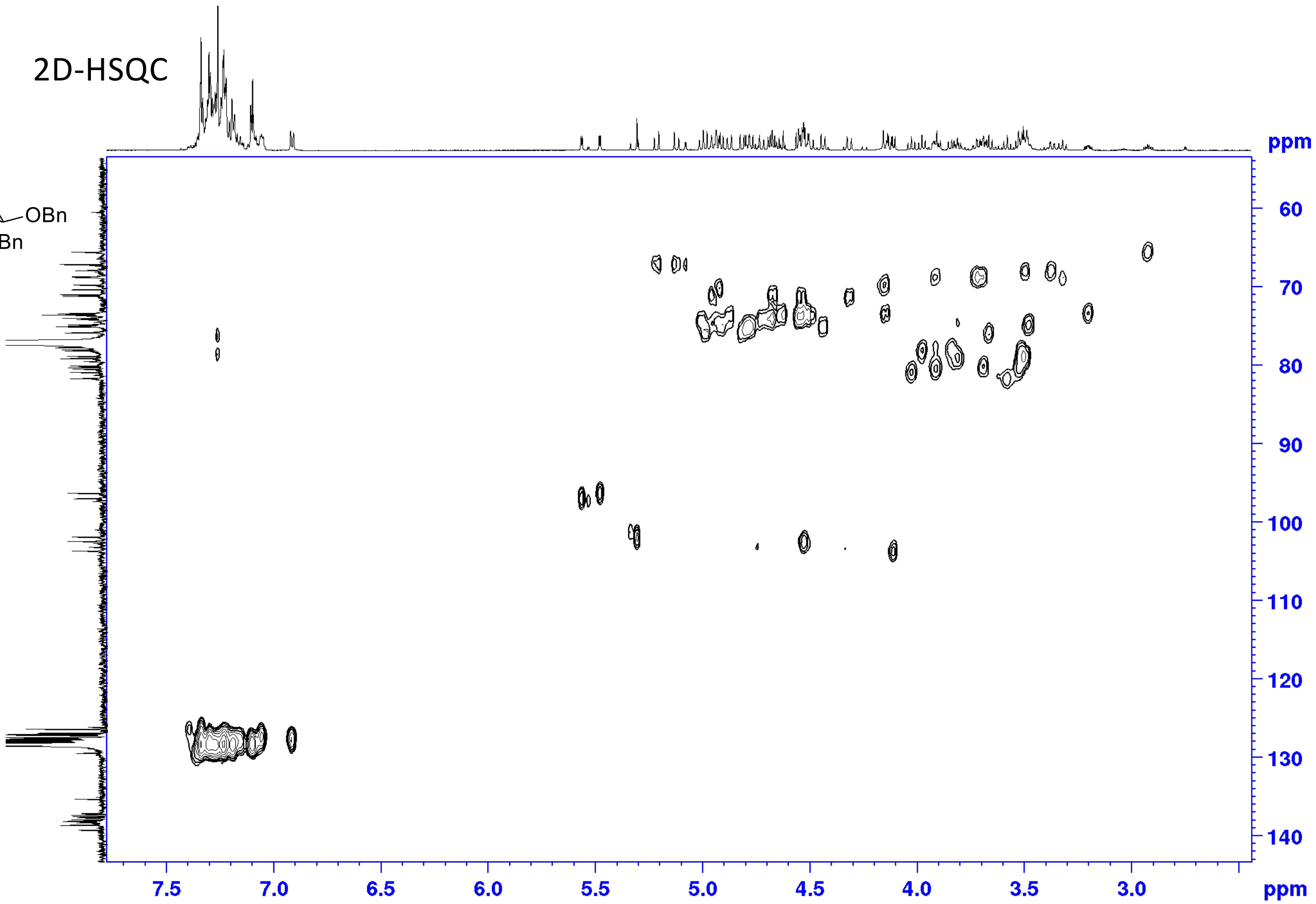

Figure S14. 2D HSQC NMR Spectrum of compound **3**

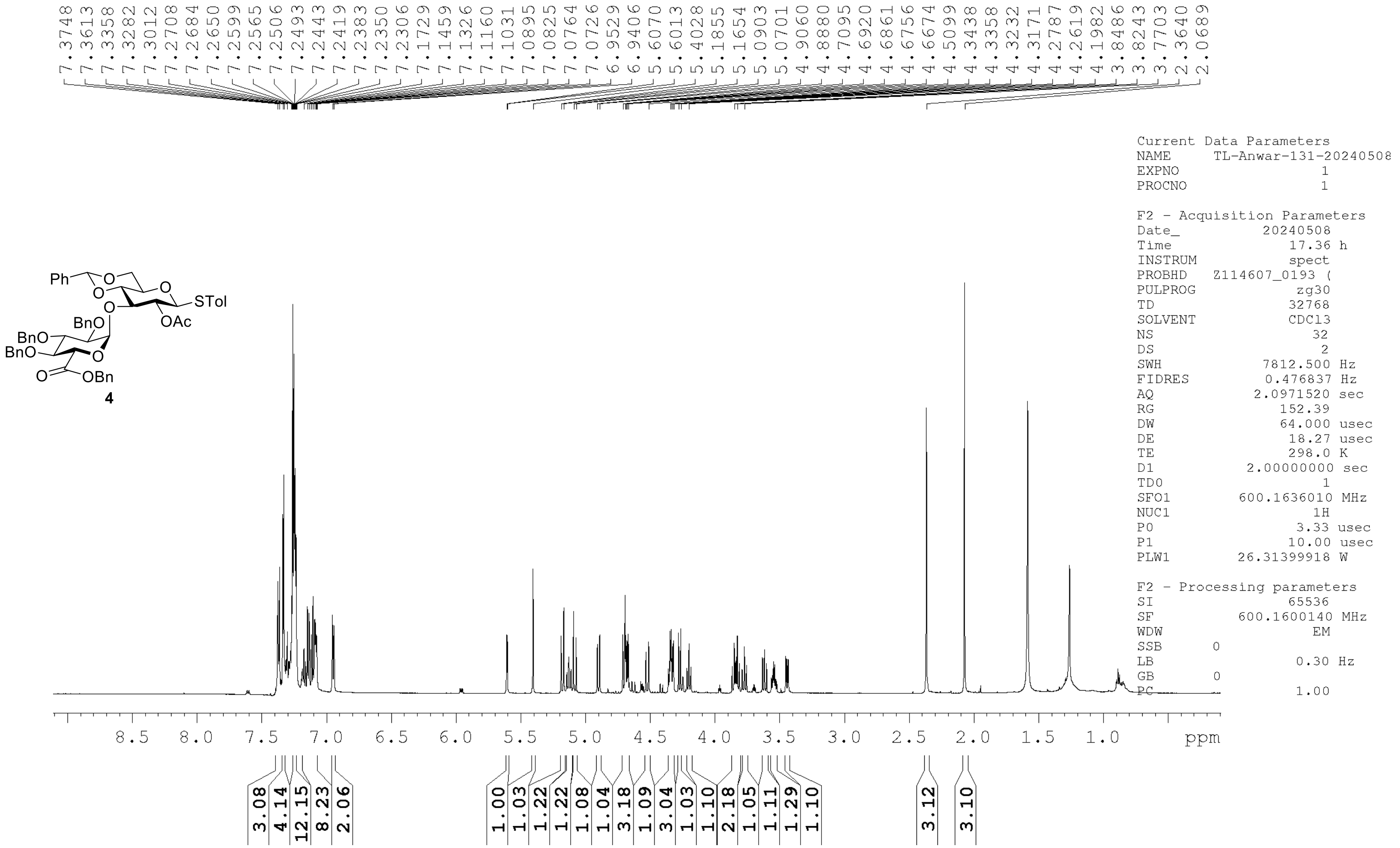

4

Figure S15. ^{1}H NMR Spectrum of compound **4**

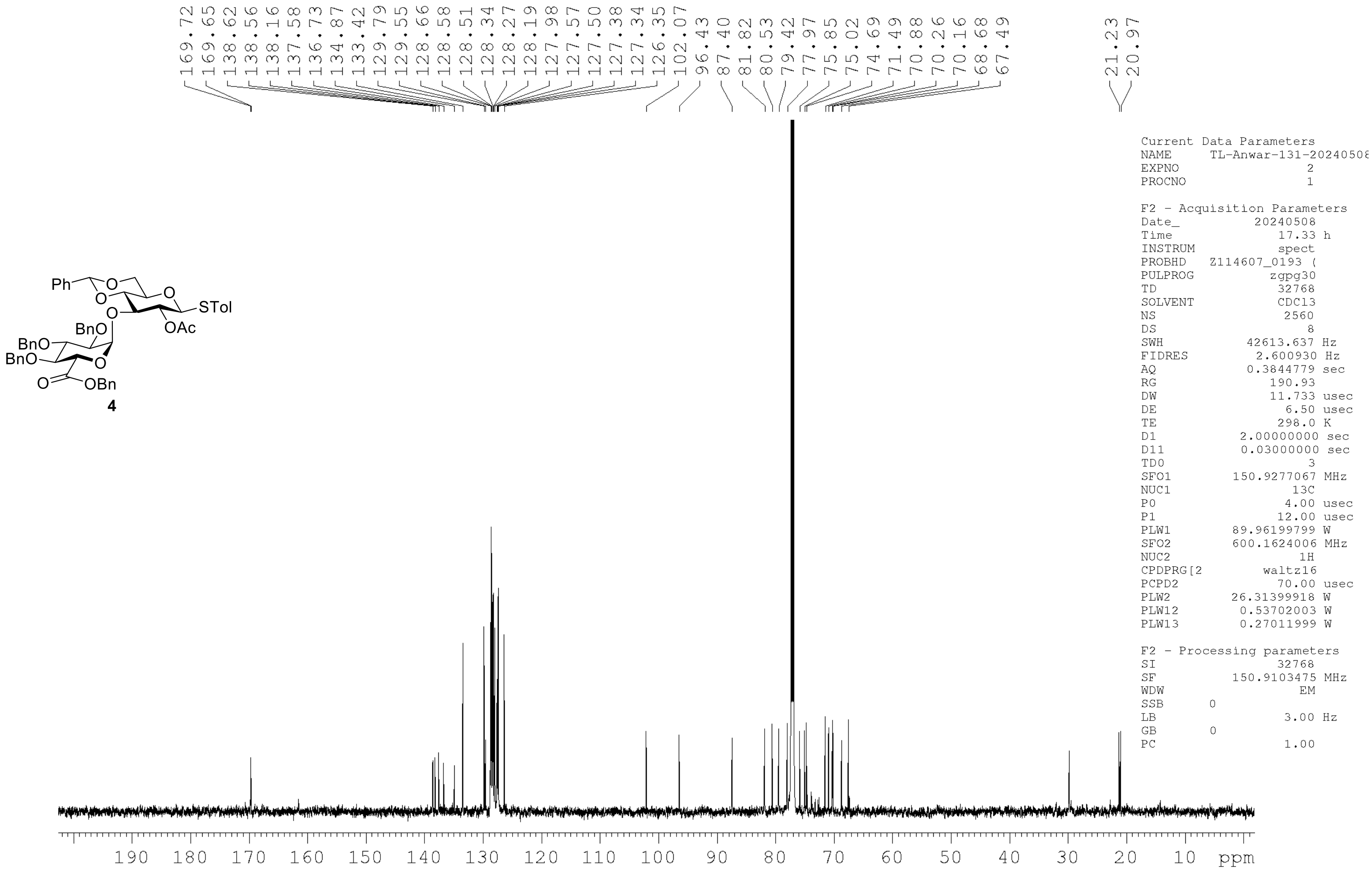

Figure S16. ^{13}C NMR Spectrum of compound **4**

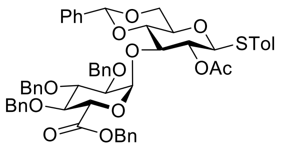

4

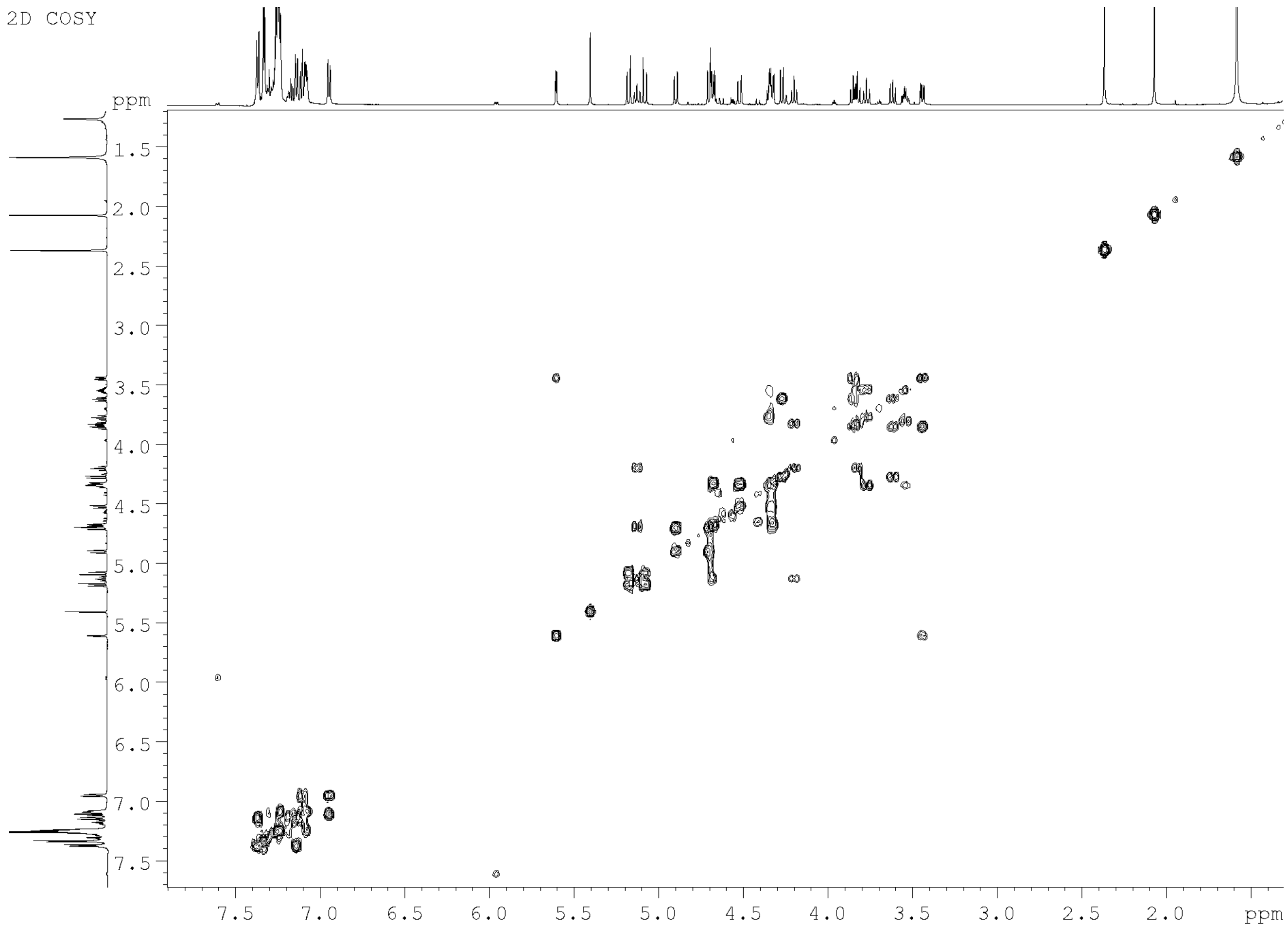

Figure S17. 2D COSY NMR Spectrum of compound **4**

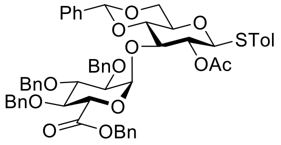

4

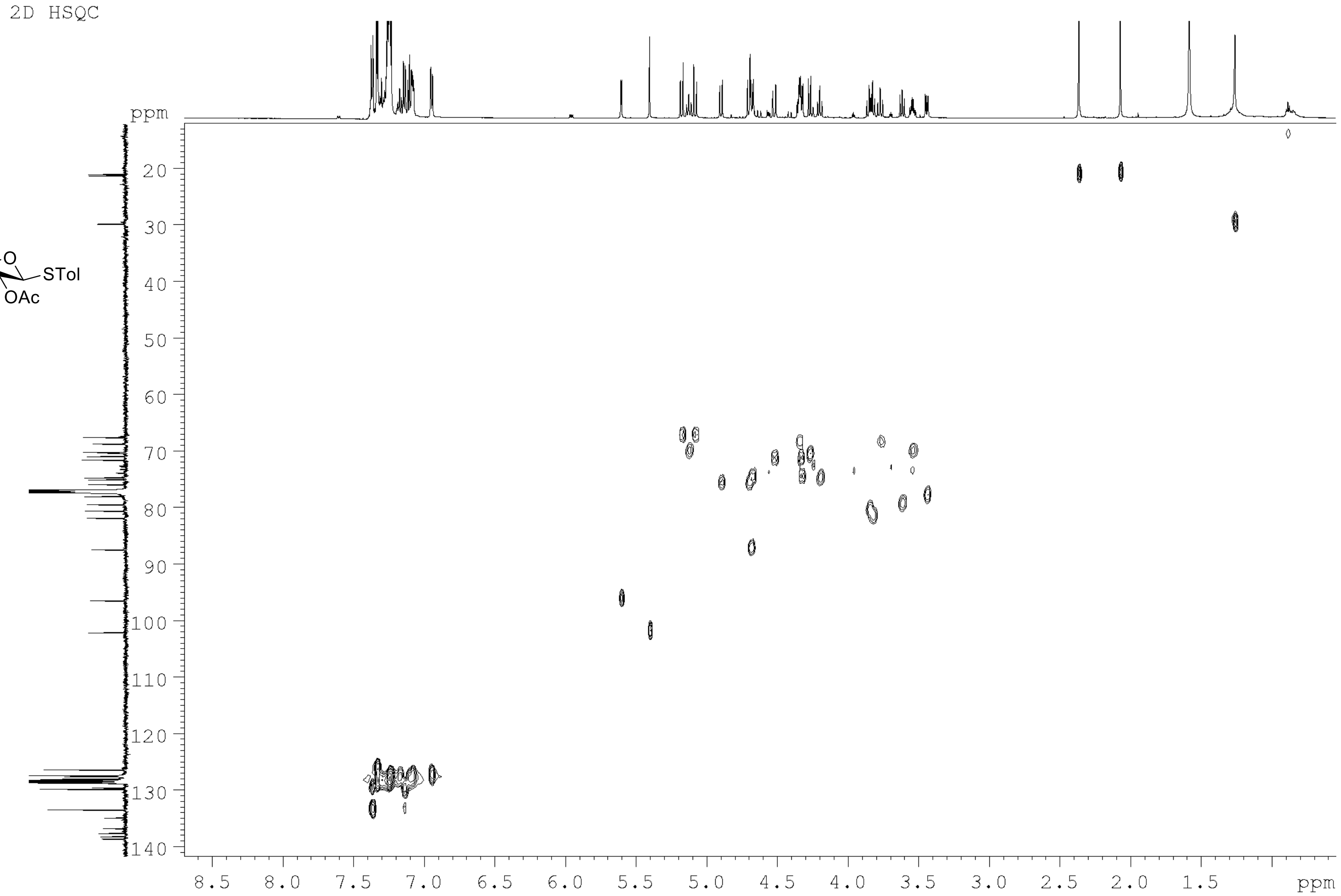

Figure S18. 2D HSQC NMR Spectrum of compound **4**

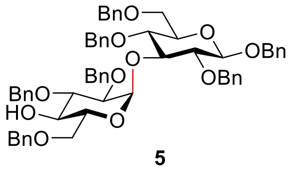

5

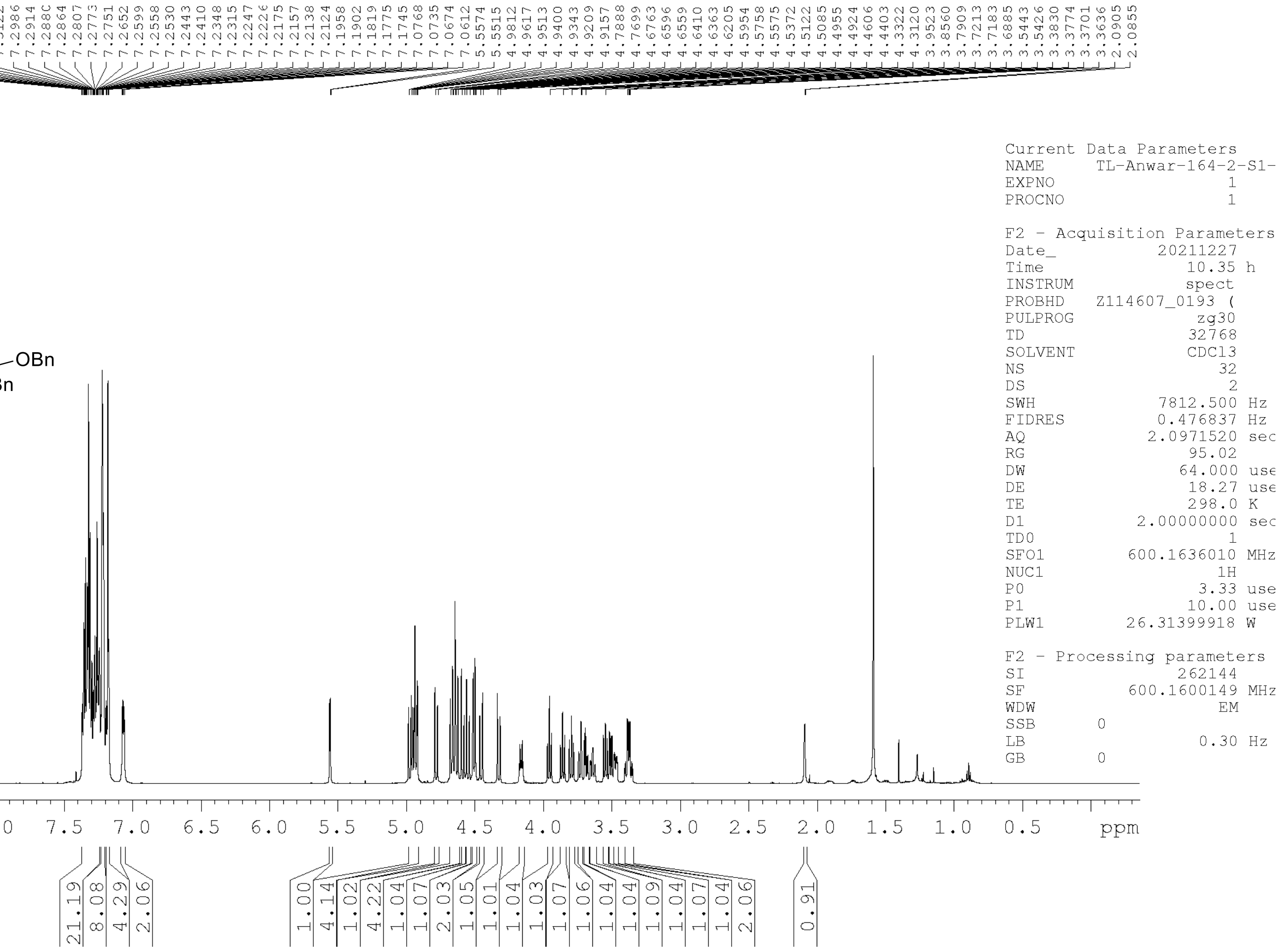

Figure S19. ^{1}H NMR Spectrum of compound **5**

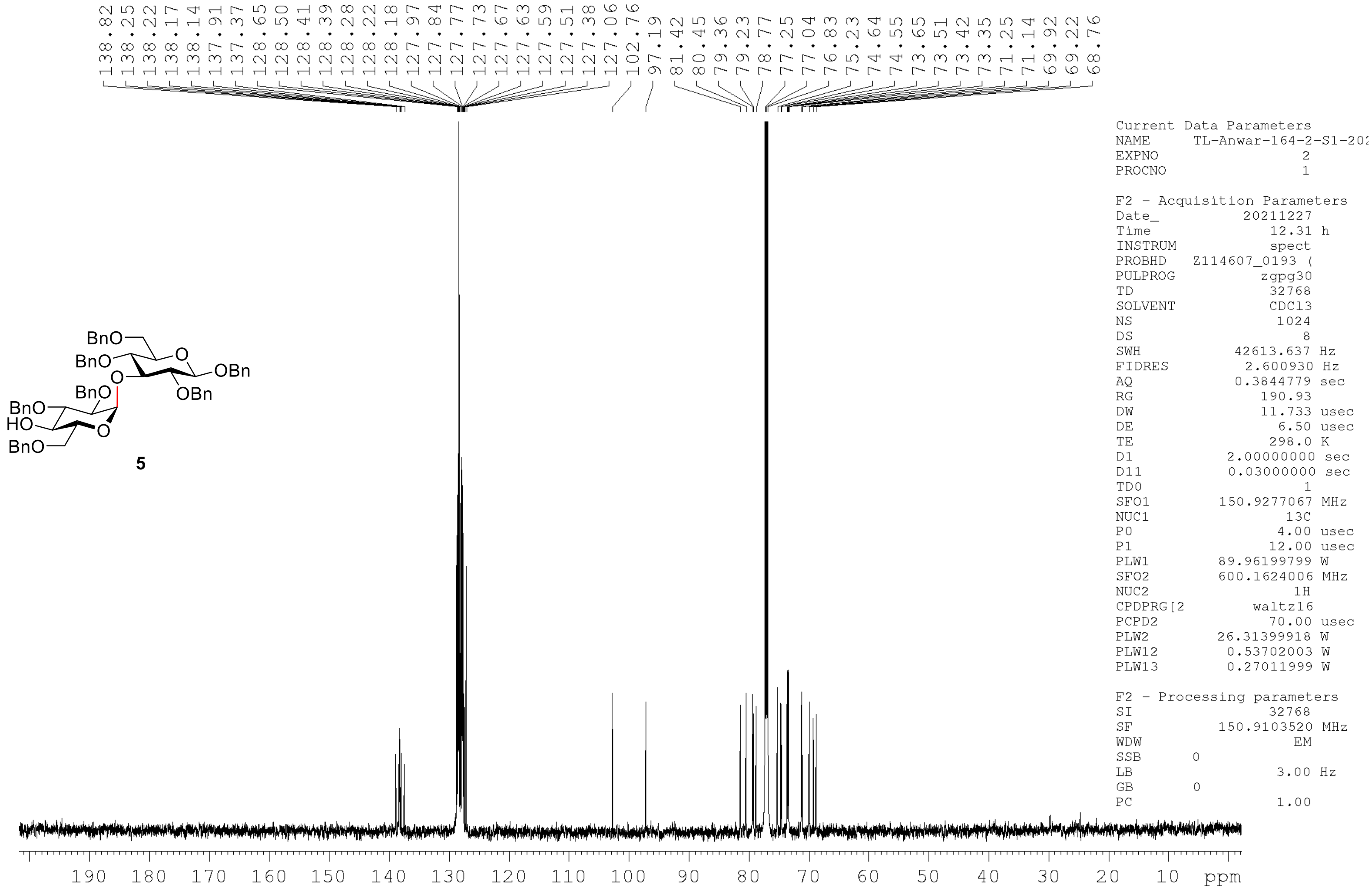

Current Data Parameters
NAME TL-Anwar-164-2-S1-202
EXPNO 2
PROCNO 1

F2 - Acquisition Parameters
Date_ 20211227
Time 12.31 h
INSTRUM spect
PROBHD Z114607_0193 (
PULPROG zgpg30
TD 32768
SOLVENT CDCl3
NS 1024
DS 8
SWH 42613.637 Hz
FIDRES 2.600930 Hz
AQ 0.3844779 sec
RG 190.93
DW 11.733 usec
DE 6.50 usec
TE 298.0 K
D1 2.00000000 sec
D11 0.03000000 sec
TD0 1
SFO1 150.9277067 MHz
NUC1 13C
P0 4.00 usec
P1 12.00 usec
PLW1 89.96199799 W
SFO2 600.1624006 MHz
NUC2 1H
CPDPRG[2 waltz16
PCPD2 70.00 usec
PLW2 26.31399918 W
PLW12 0.53702003 W
PLW13 0.27011999 W

F2 - Processing parameters
SI 32768
SF 150.9103520 MHz
WDW EM
SSB 0
LB 3.00 Hz
GB 0
PC 1.00

Figure S20. ^{13}C NMR Spectrum of compound **5**

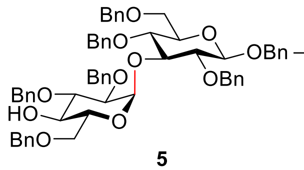

5

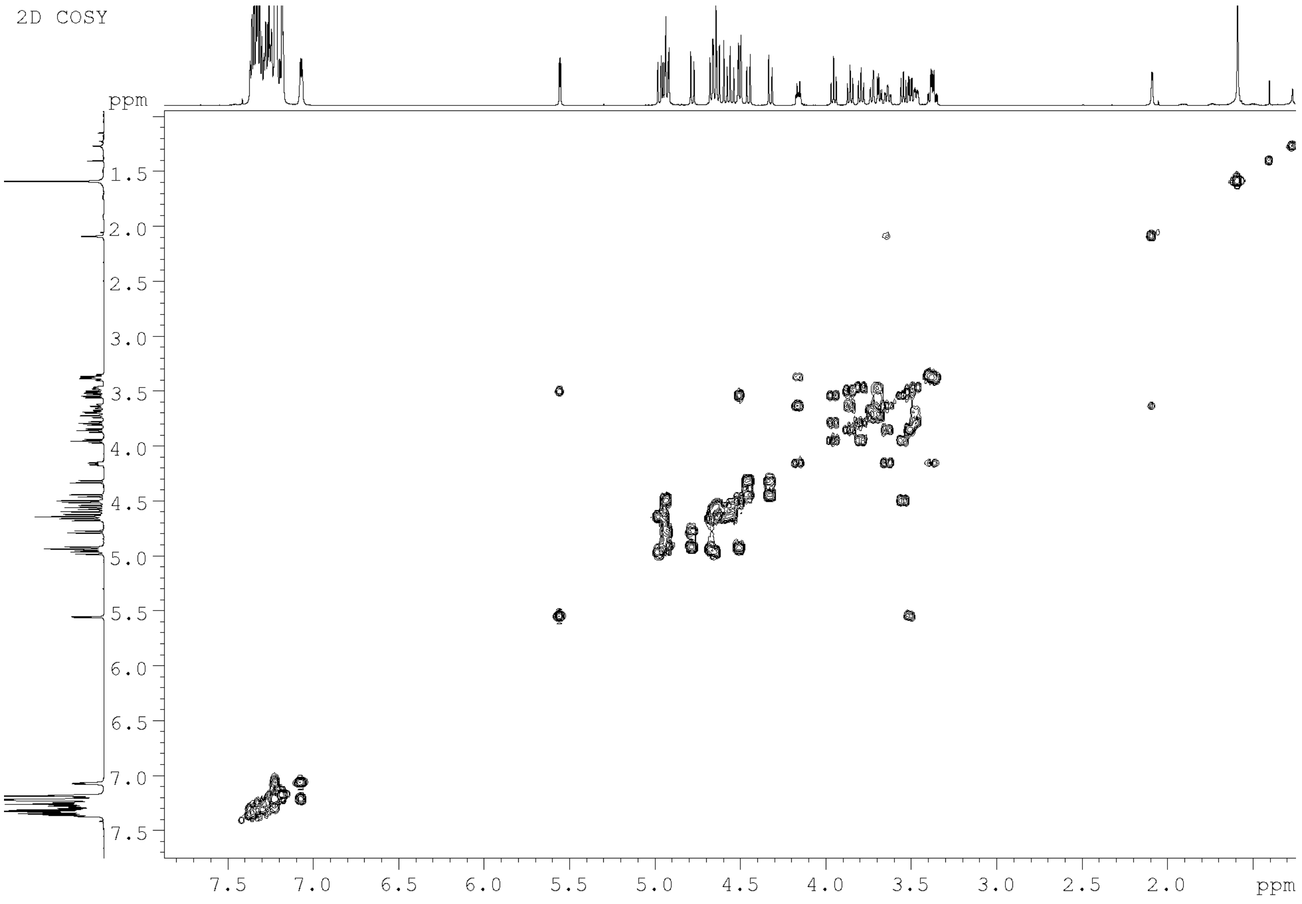

Figure S21. 2D COSY NMR Spectrum of compound **5**

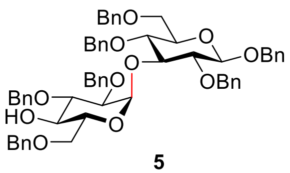

5

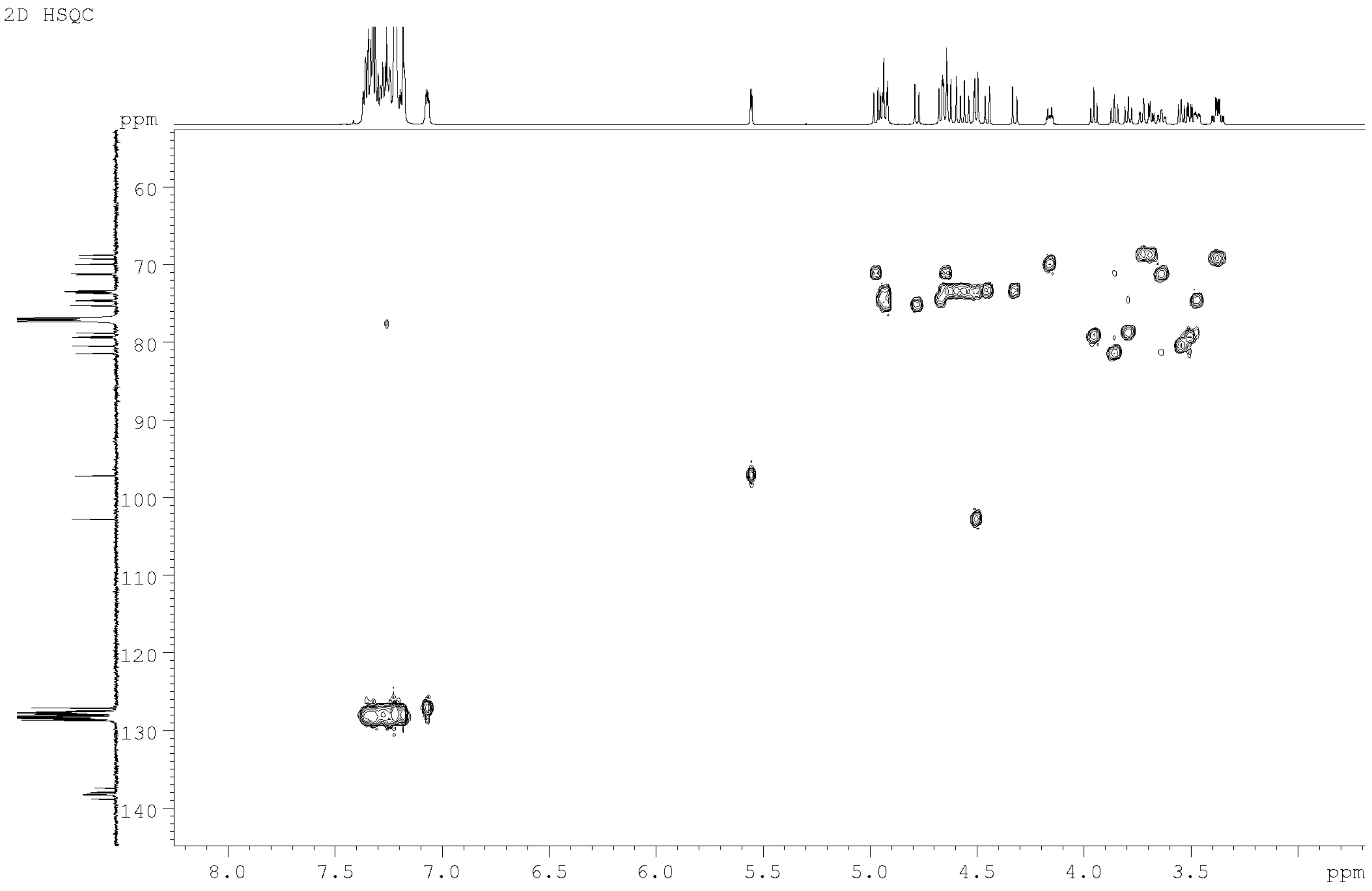

Figure S22. 2D HSQC NMR Spectrum of compound **5**

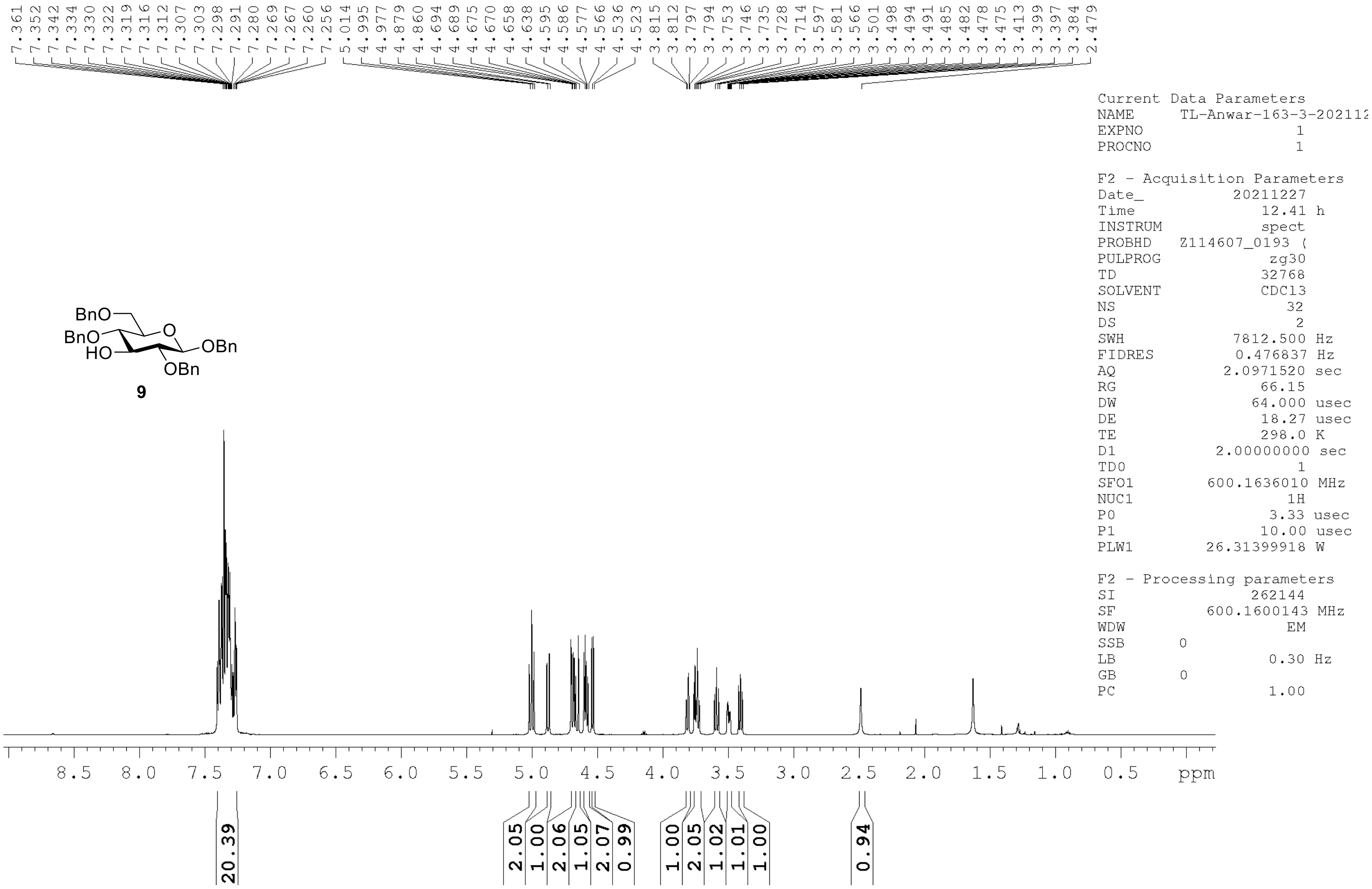

Figure S23. ^{1}H NMR Spectrum of compound **9**

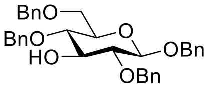

9

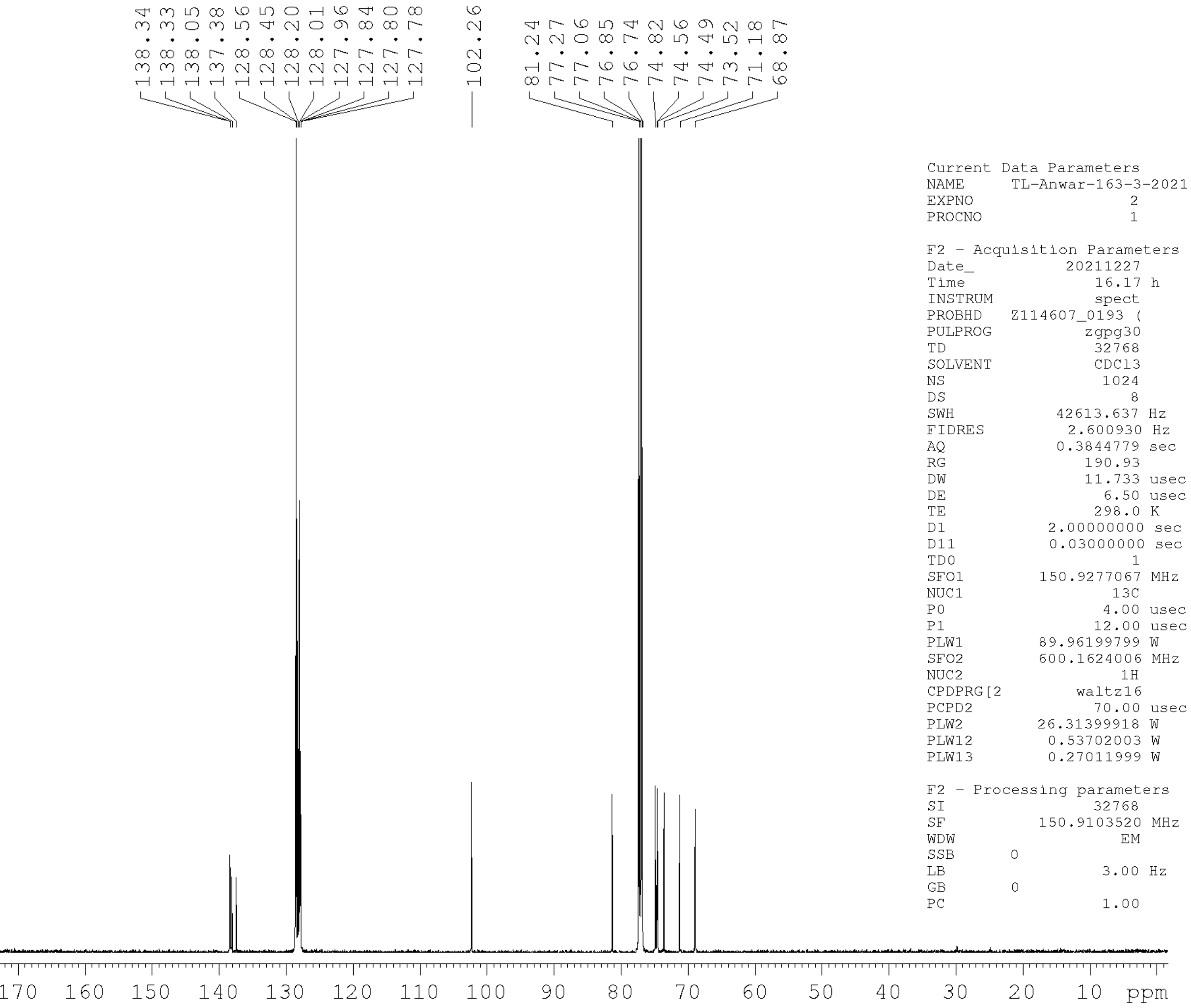


```
Current Data Parameters
NAME      TL-Anwar-163-3-202112
EXPNO                 2
PROCNO                1

F2 - Acquisition Parameters
Date_          20211227
Time              16.17 h
INSTRUM           spect
PROBHD   Z114607_0193 (
PULPROG         zgpg30
TD                32768
SOLVENT           CDCl3
NS                 1024
DS                    8
SWH           42613.637 Hz
FIDRES         2.600930 Hz
AQ            0.3844779 sec
RG               190.93
DW               11.733 usec
DE                 6.50 usec
TE                298.0 K
D1           2.00000000 sec
D11          0.03000000 sec
TD0                   1
SFO1        150.9277067 MHz
NUC1                13C
P0                 4.00 usec
P1                12.00 usec
PLW1        89.96199799 W
SFO2        600.1624006 MHz
NUC2                 1H
CPDPRG[2        waltz16
PCPD2             70.00 usec
PLW2        26.31399918 W
PLW12        0.53702003 W
PLW13        0.27011999 W

F2 - Processing parameters
SI                32768
SF          150.9103520 MHz
WDW                  EM
SSB      0
LB                 3.00 Hz
GB       0
PC                 1.00
```

Figure S24. ^{13}H NMR Spectrum of compound **9**

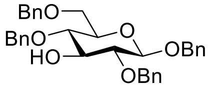

9

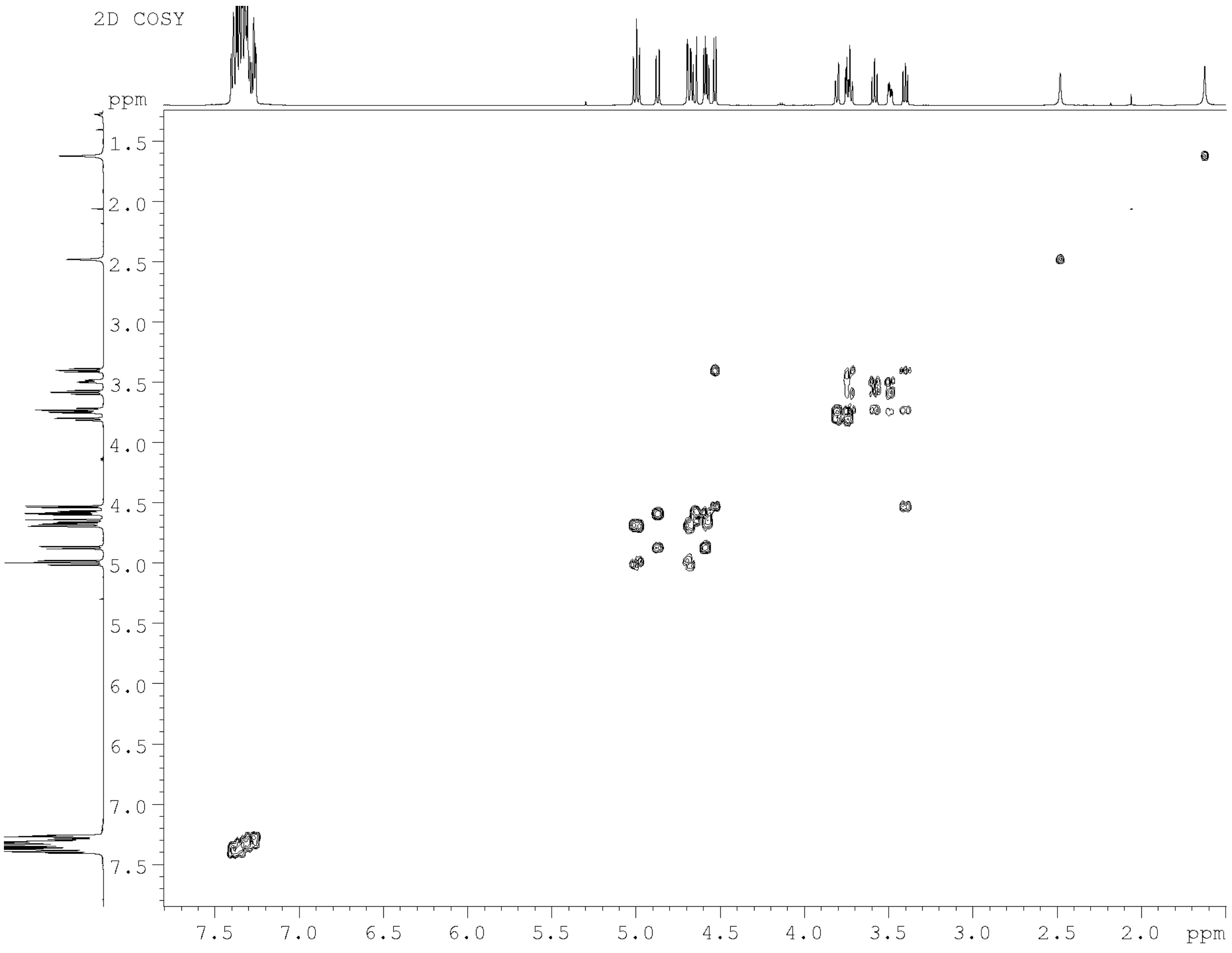

Figure S25. 2D COSY NMR Spectrum of compound **9**

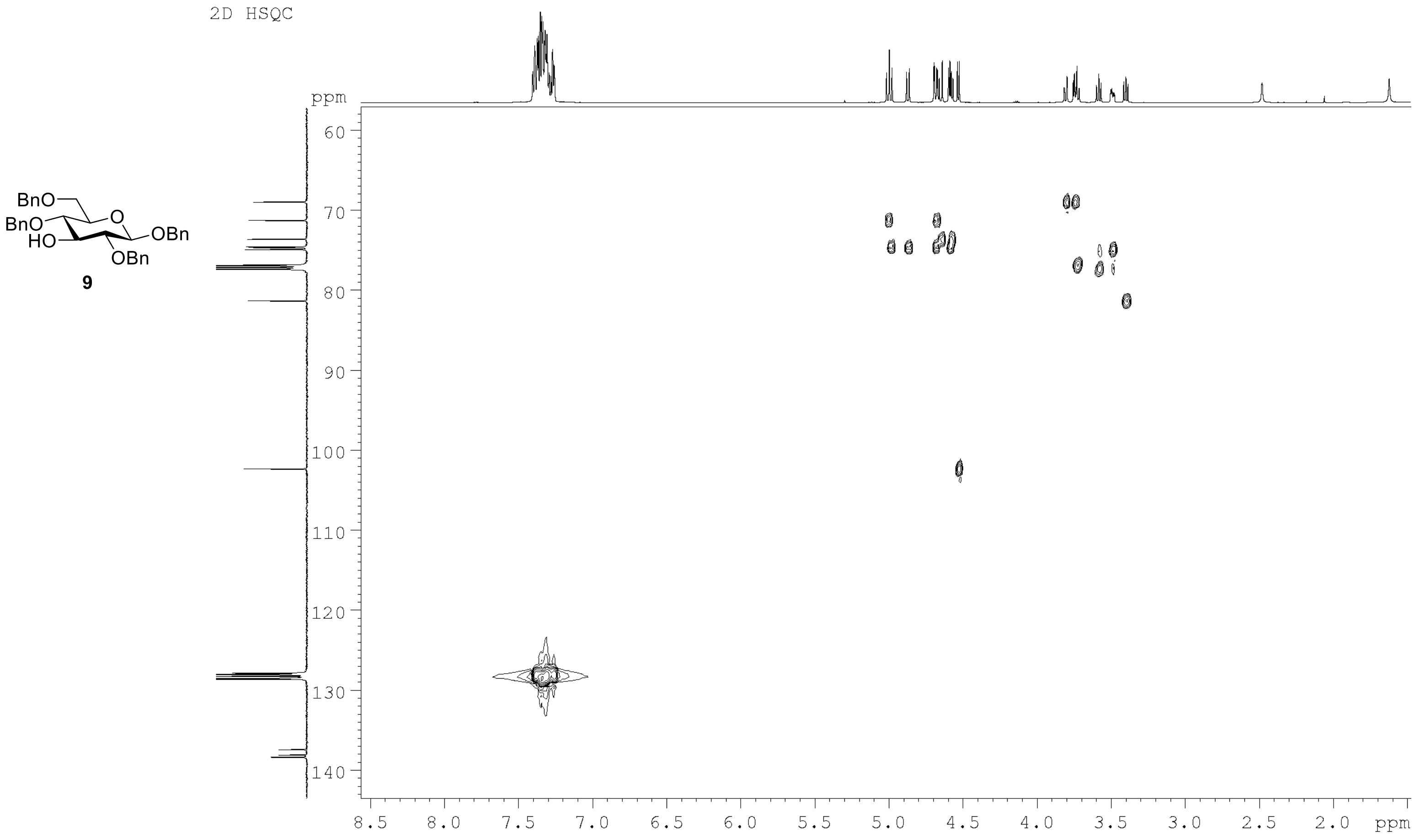

Figure S26. 2D HSQC NMR Spectrum of compound **9**

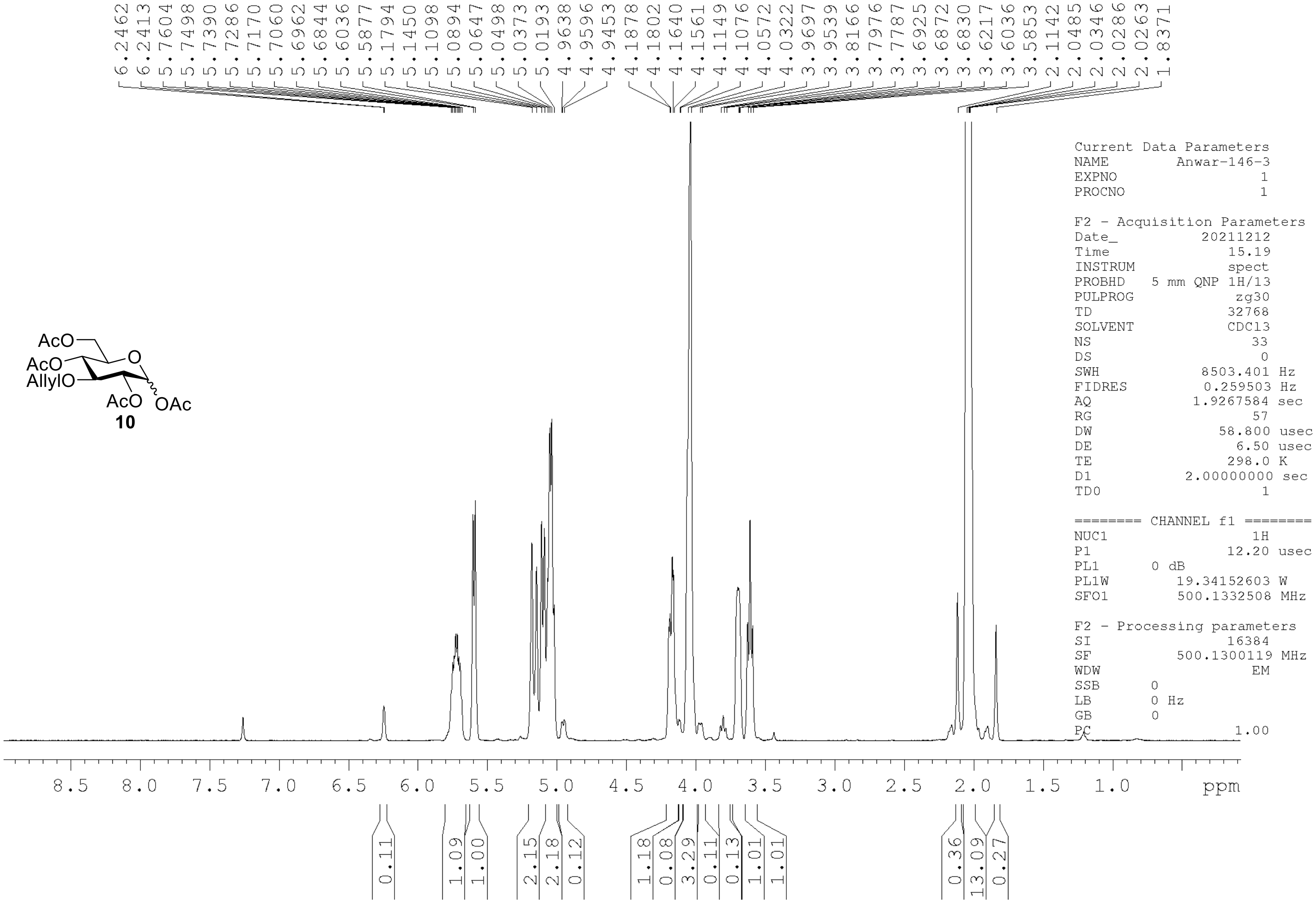

Figure S27. ^{1}H NMR Spectrum of compound **10**

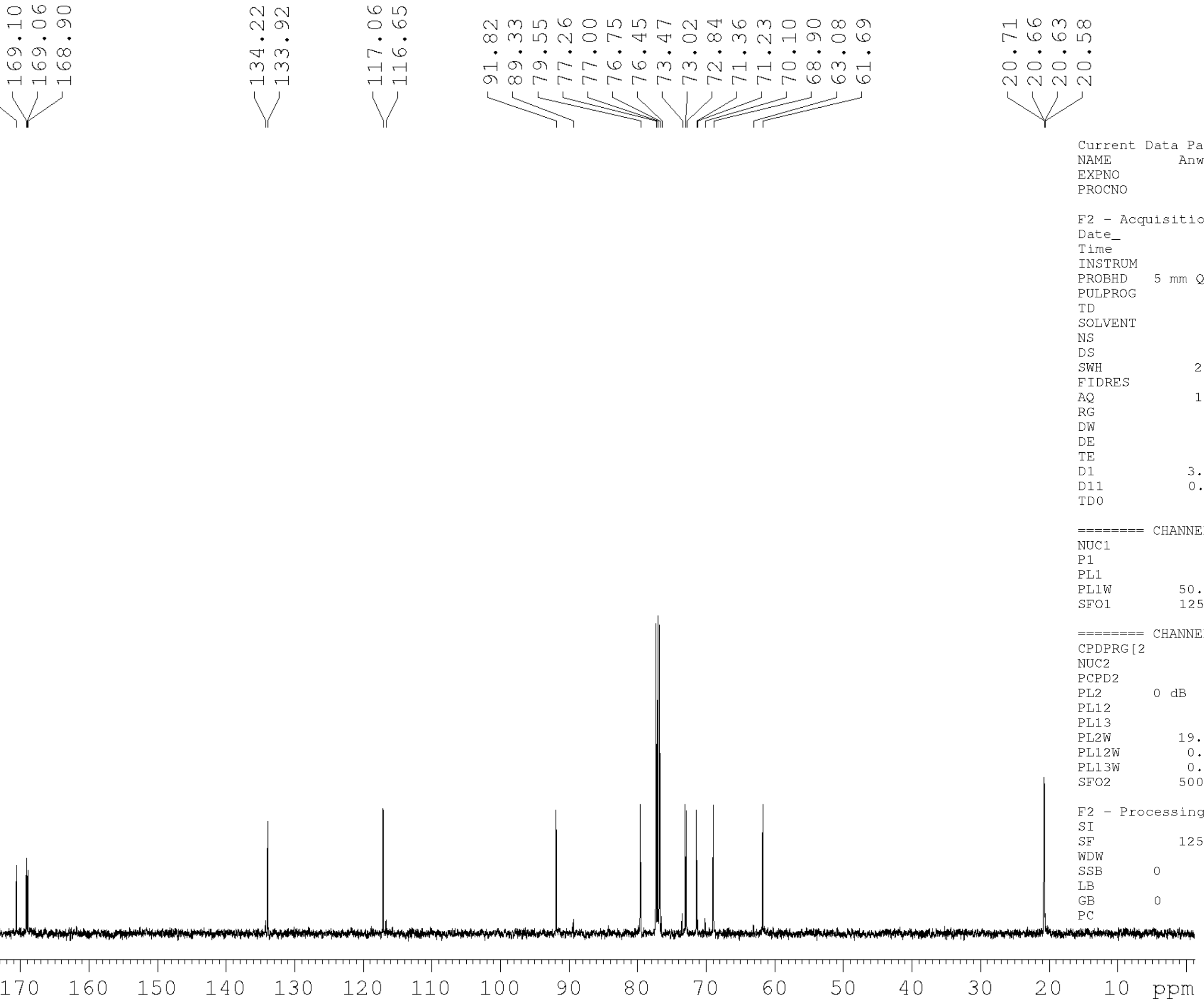

Current Data Parameters
NAME Anwar-146-3
EXPNO 2
PROCNO 1

F2 - Acquisition Parameters
Date_ 20211212
Time 15.23
INSTRUM spect
PROBHD 5 mm QNP 1H/13
PULPROG zgpg30
TD 65536
SOLVENT CDCl3
NS 34
DS 0
SWH 29761.904 Hz
FIDRES 0.454131 Hz
AQ 1.1010048 sec
RG 2050
DW 16.800 usec
DE 6.50 usec
TE 298.0 K
D1 3.00000000 sec
D11 0.03000000 sec
TD0 1

======== CHANNEL f1 ========
NUC1 13C
P1 9.60 usec
PL1 2.00 dB
PL1W 50.08262634 W
SFO1 125.7719363 MHz

======== CHANNEL f2 ========
CPDPRG[2 waltz16
NUC2 1H
PCPD2 80.00 usec
PL2 0 dB
PL12 15.80 dB
PL13 18.80 dB
PL2W 19.34152603 W
PL12W 0.50873393 W
PL13W 0.25497100 W
SFO2 500.1320005 MHz

F2 - Processing parameters
SI 32768
SF 125.7578014 MHz
WDW EM
SSB 0
LB 3.00 Hz
GB 0
PC 1.40

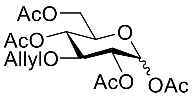

10

Figure S28. ^{13}C NMR Spectrum of compound **10**

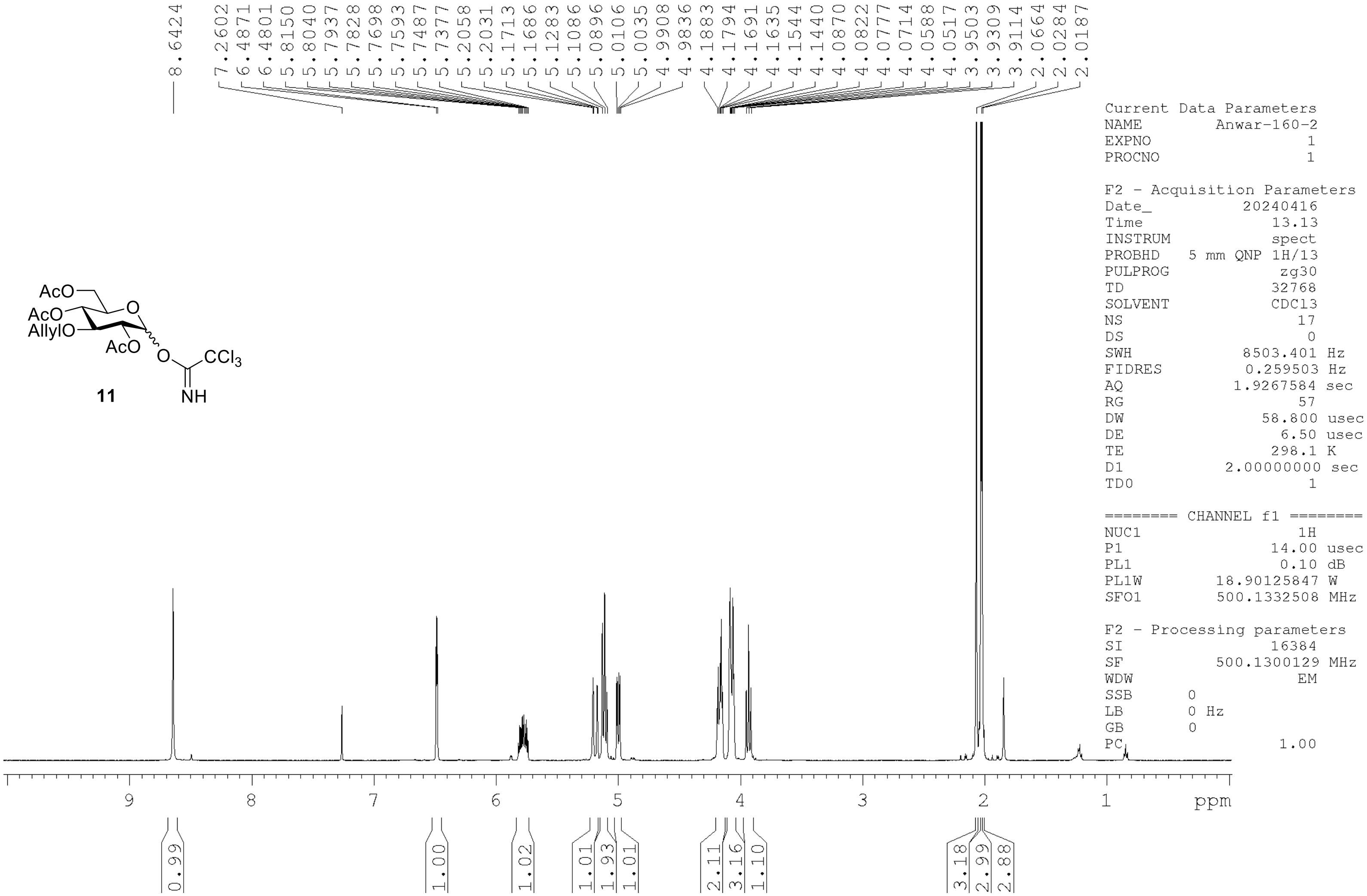

Figure S29. ^{1}H NMR Spectrum of compound **11**

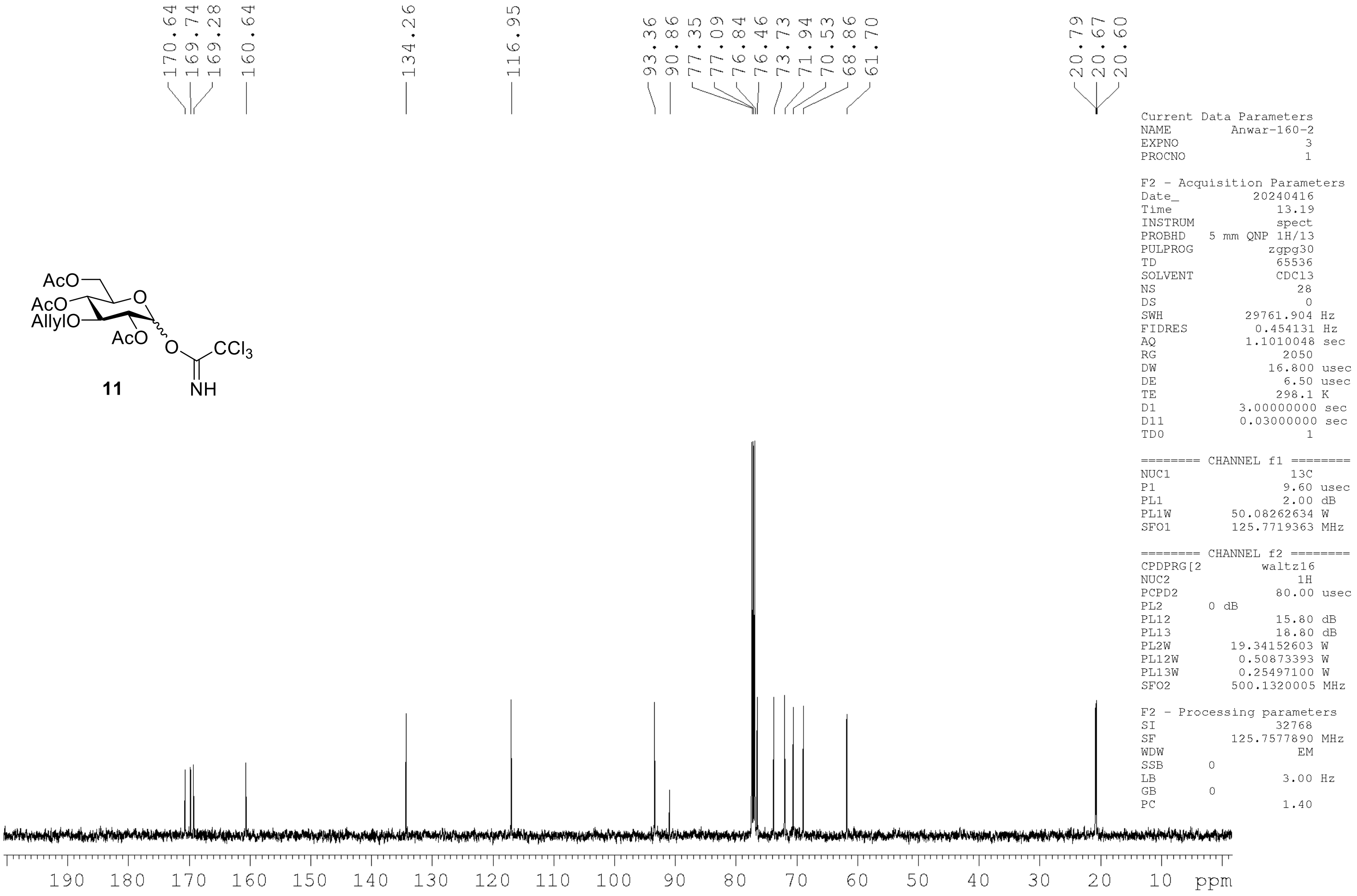

Figure S30. ^{13}C NMR Spectrum of compound **11**

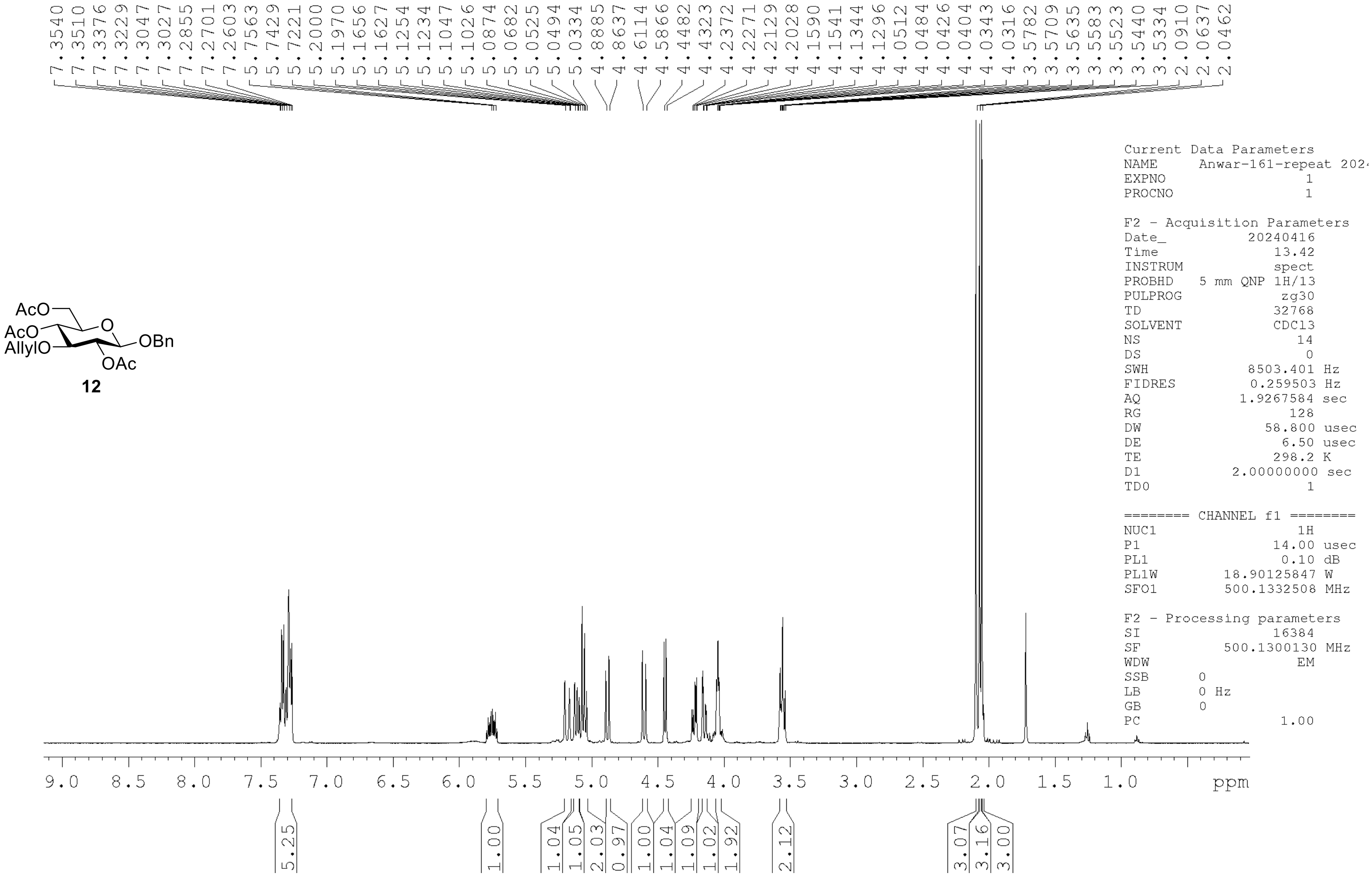

Figure S31. ^{1}H NMR Spectrum of compound **12**

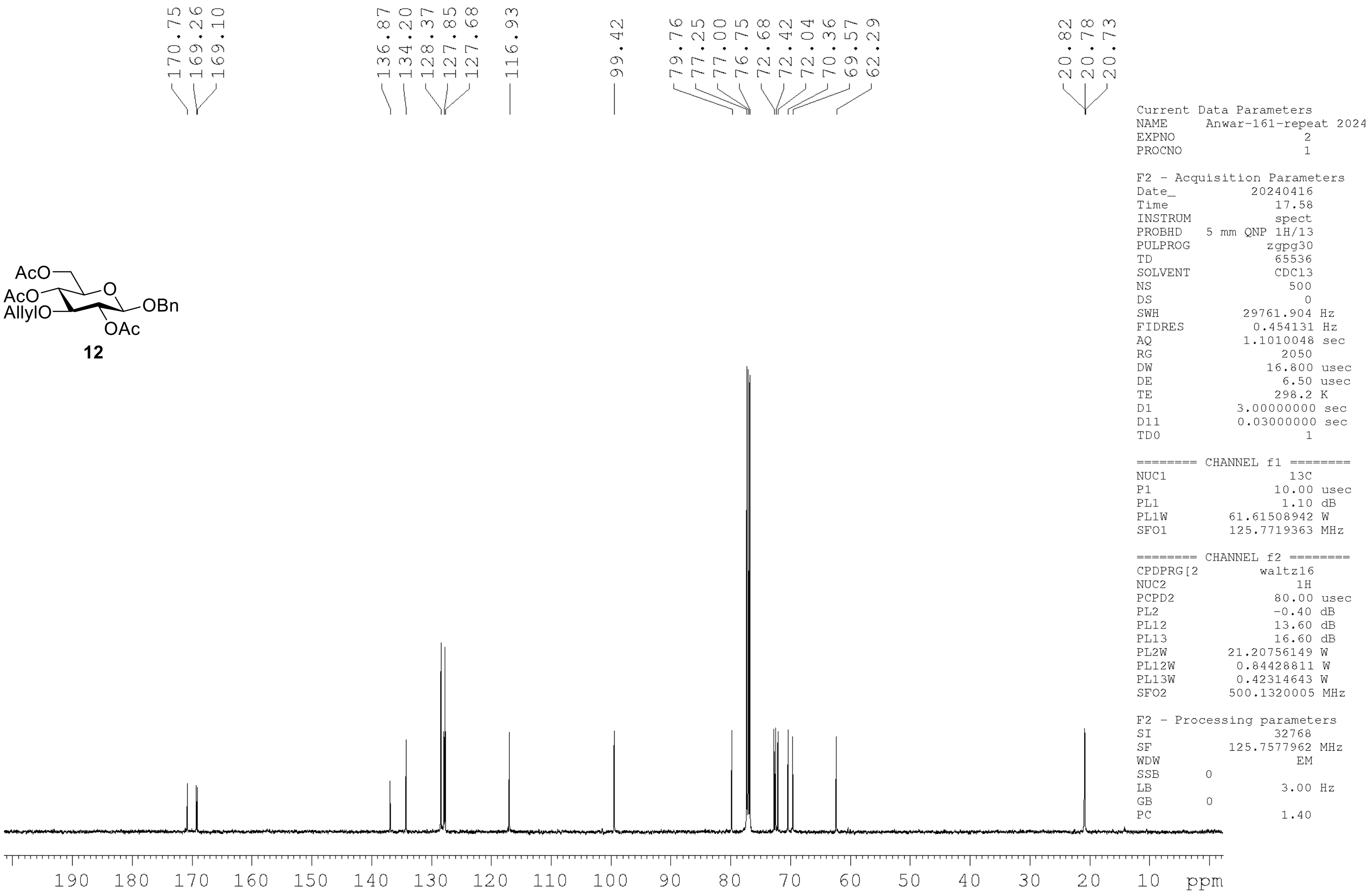

Figure S32. ^{13}C NMR Spectrum of compound **12**

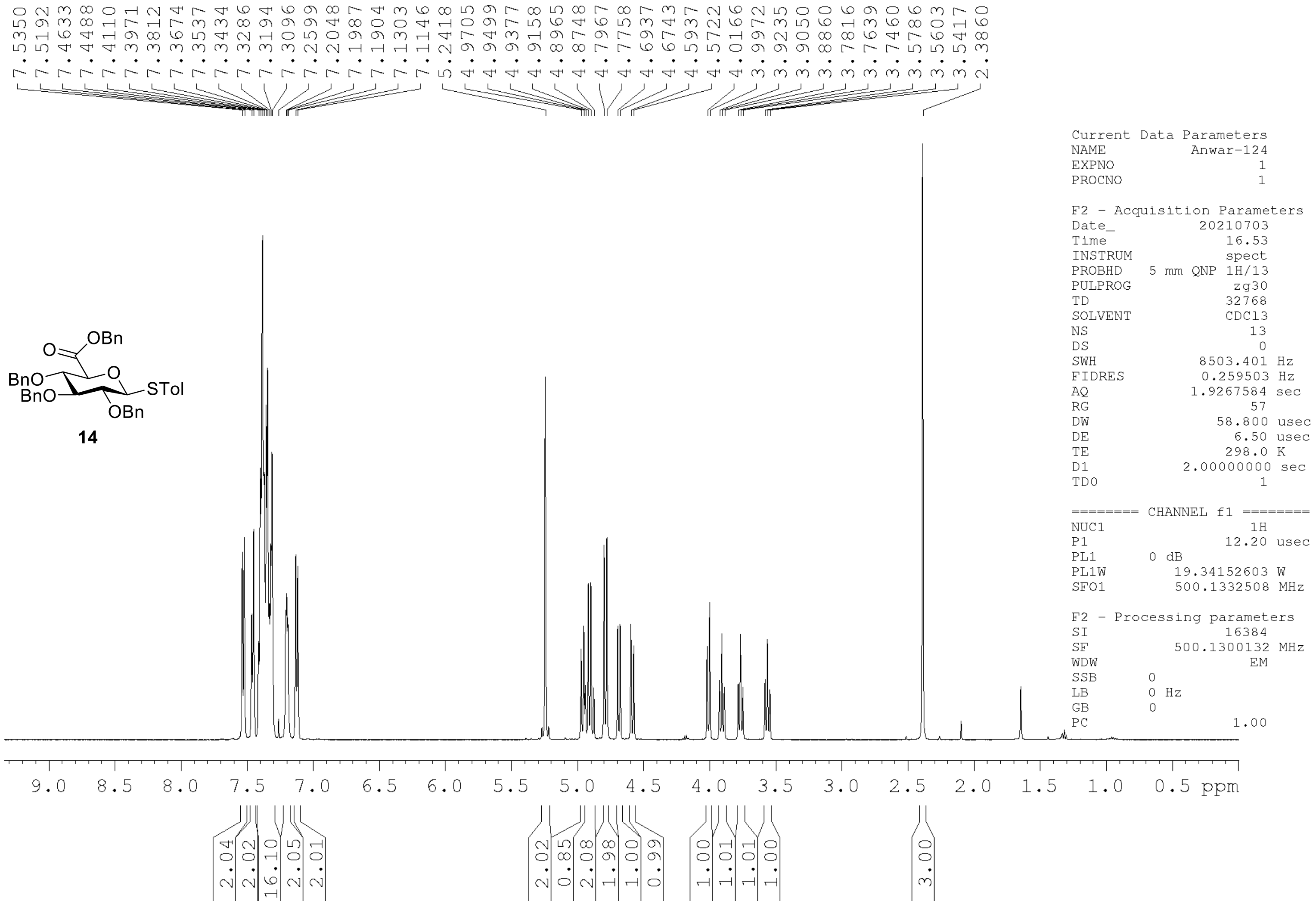

Figure S33. ^{1}H NMR Spectrum of compound **14**

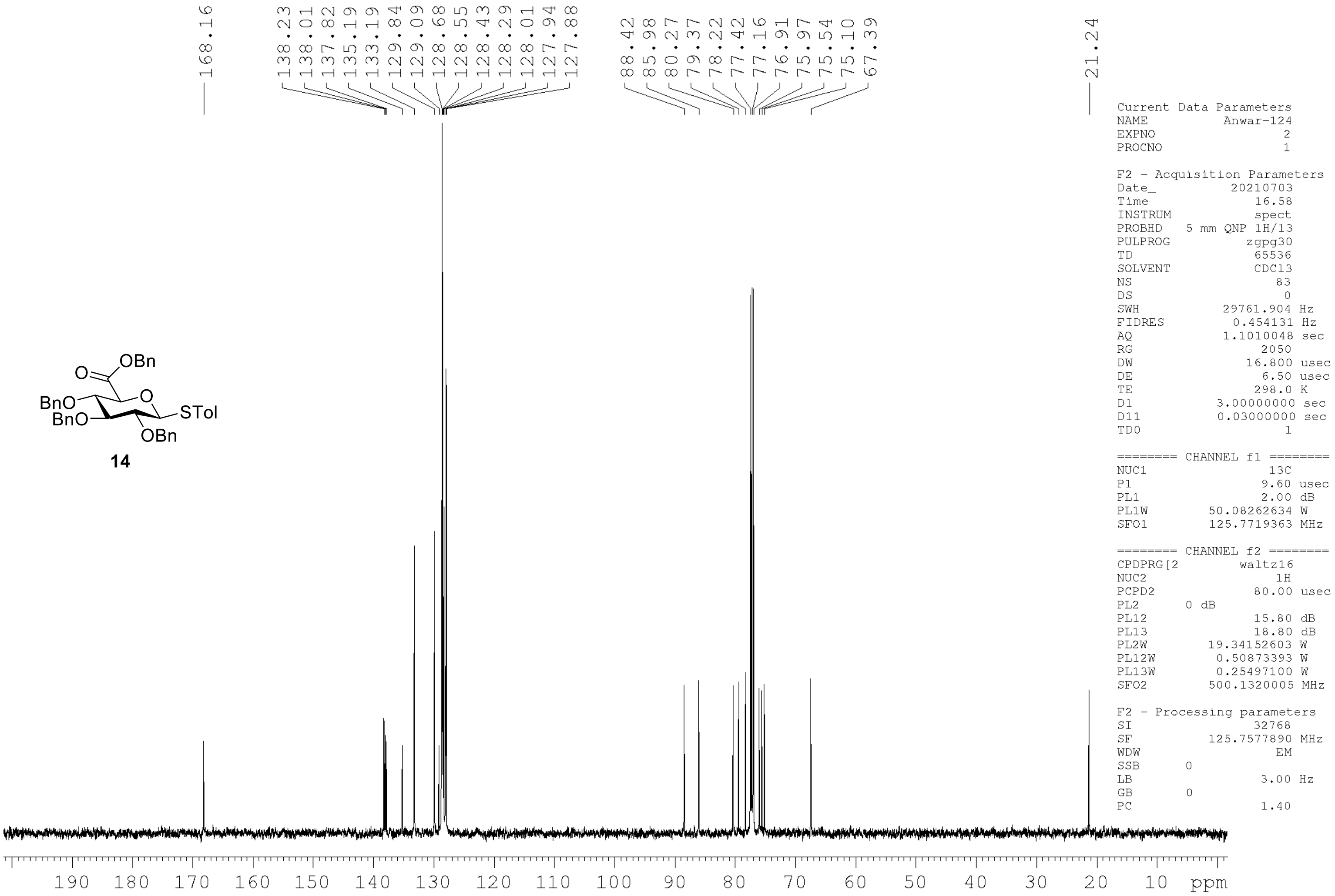

Figure S34. ^{13}C NMR Spectrum of compound **14**

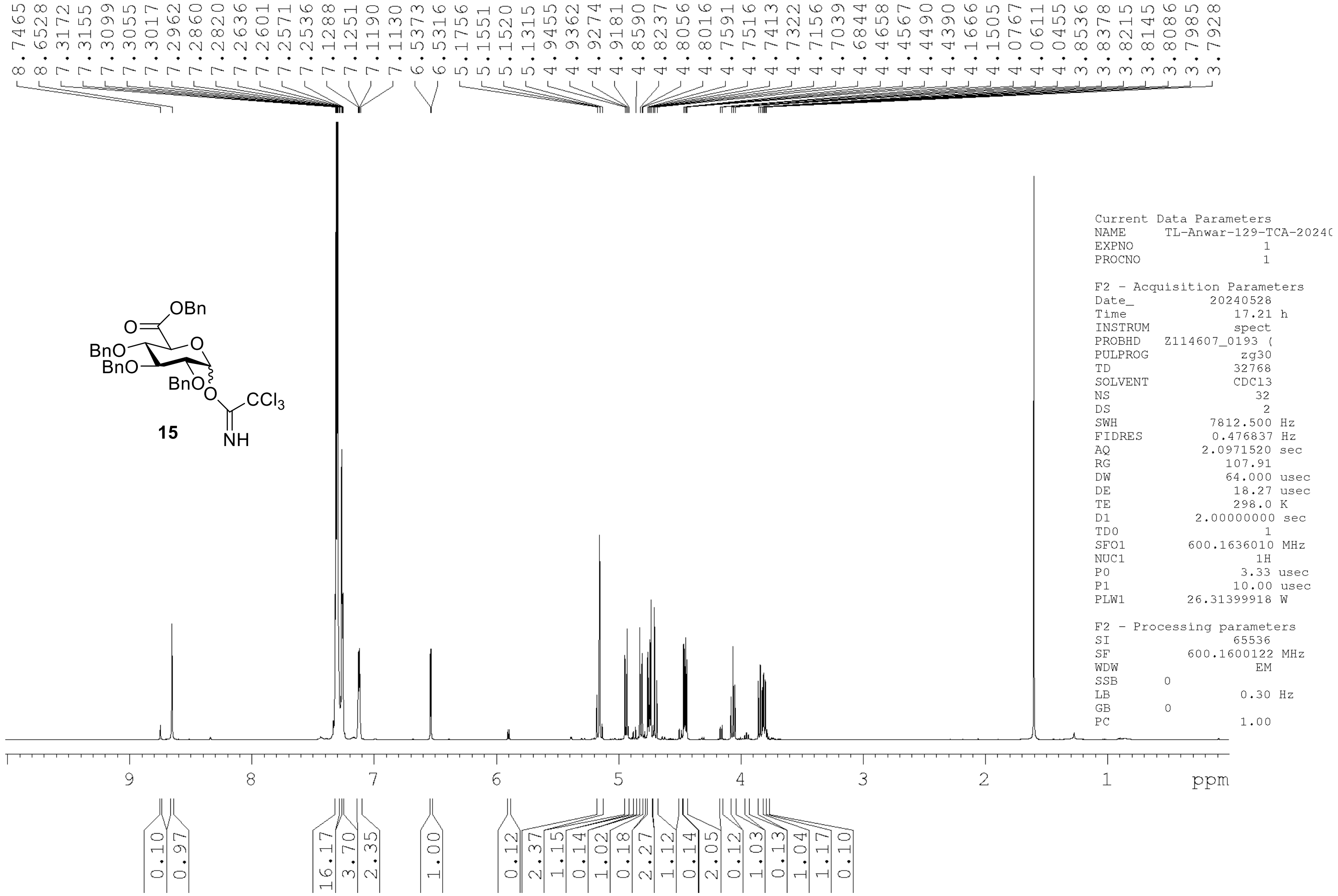

Figure S35. ^{1}H NMR Spectrum of compound **15**

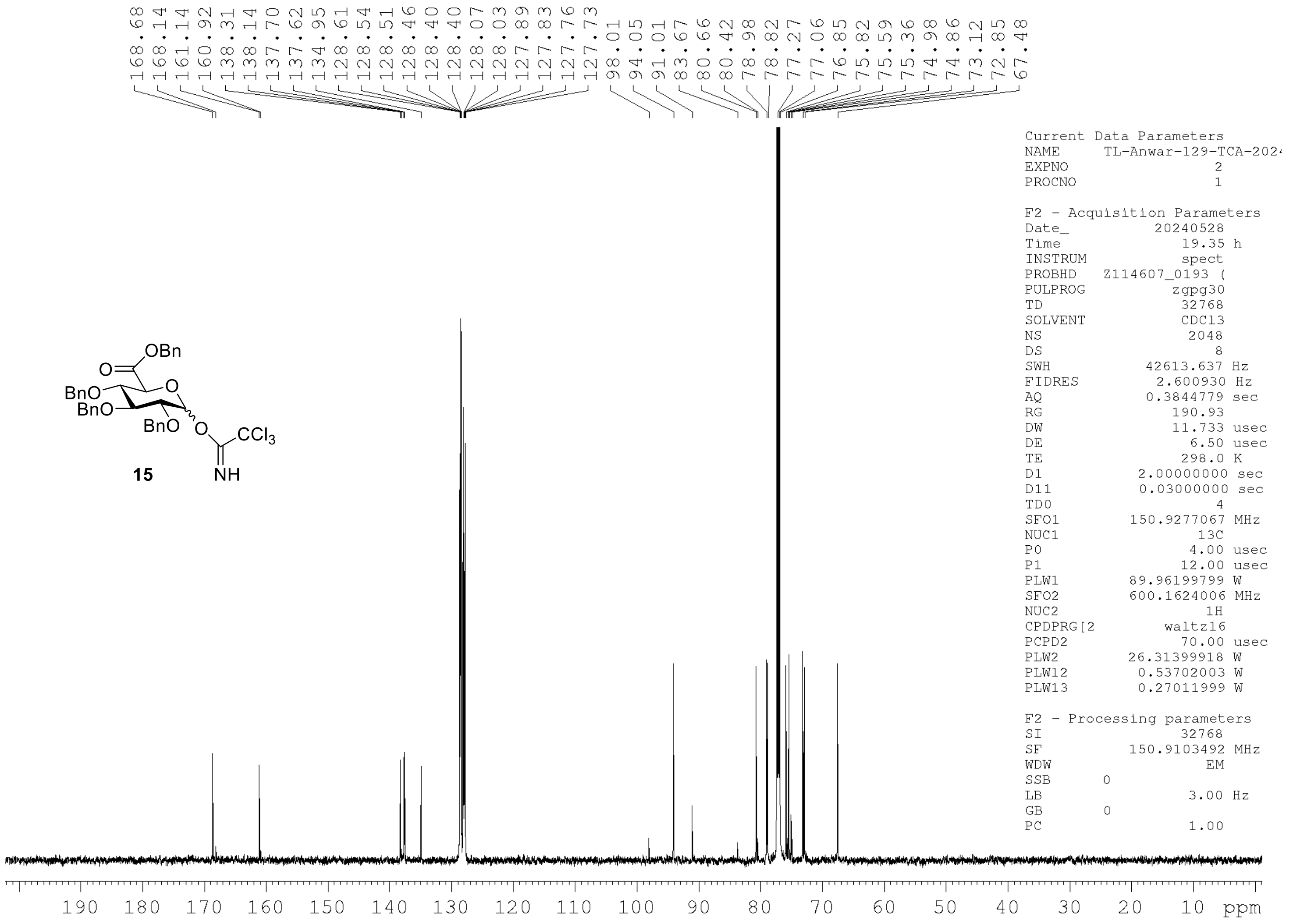

Figure S36. ^{13}C NMR Spectrum of compound **15**

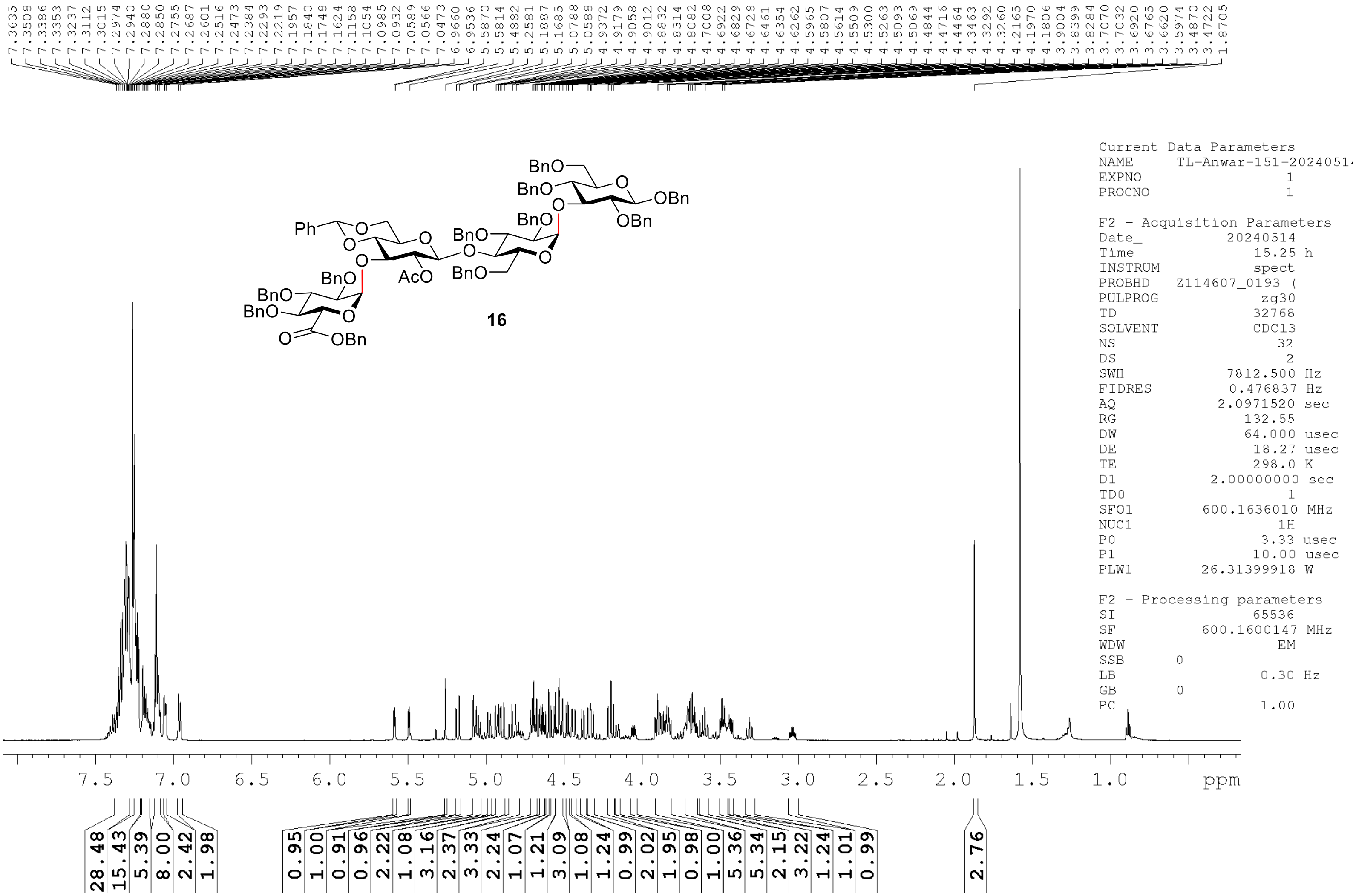

Figure S37. ^{1}H NMR Spectrum of compound **16**

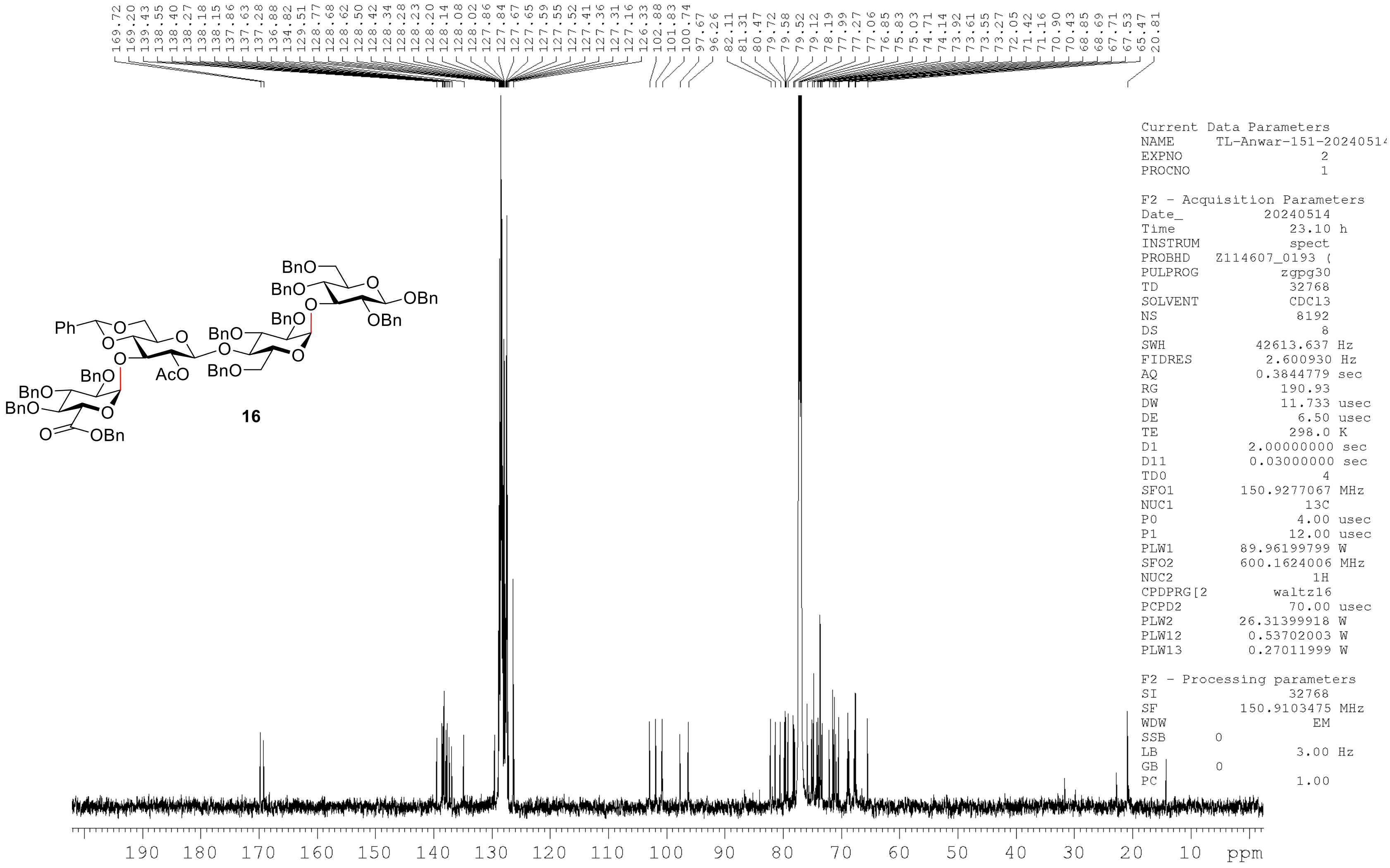

Figure S38. ^{13}C NMR Spectrum of compound **16**

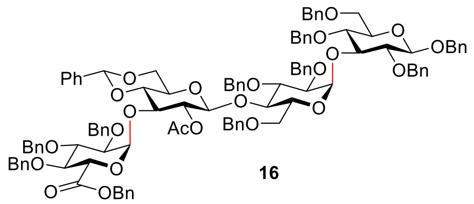

16

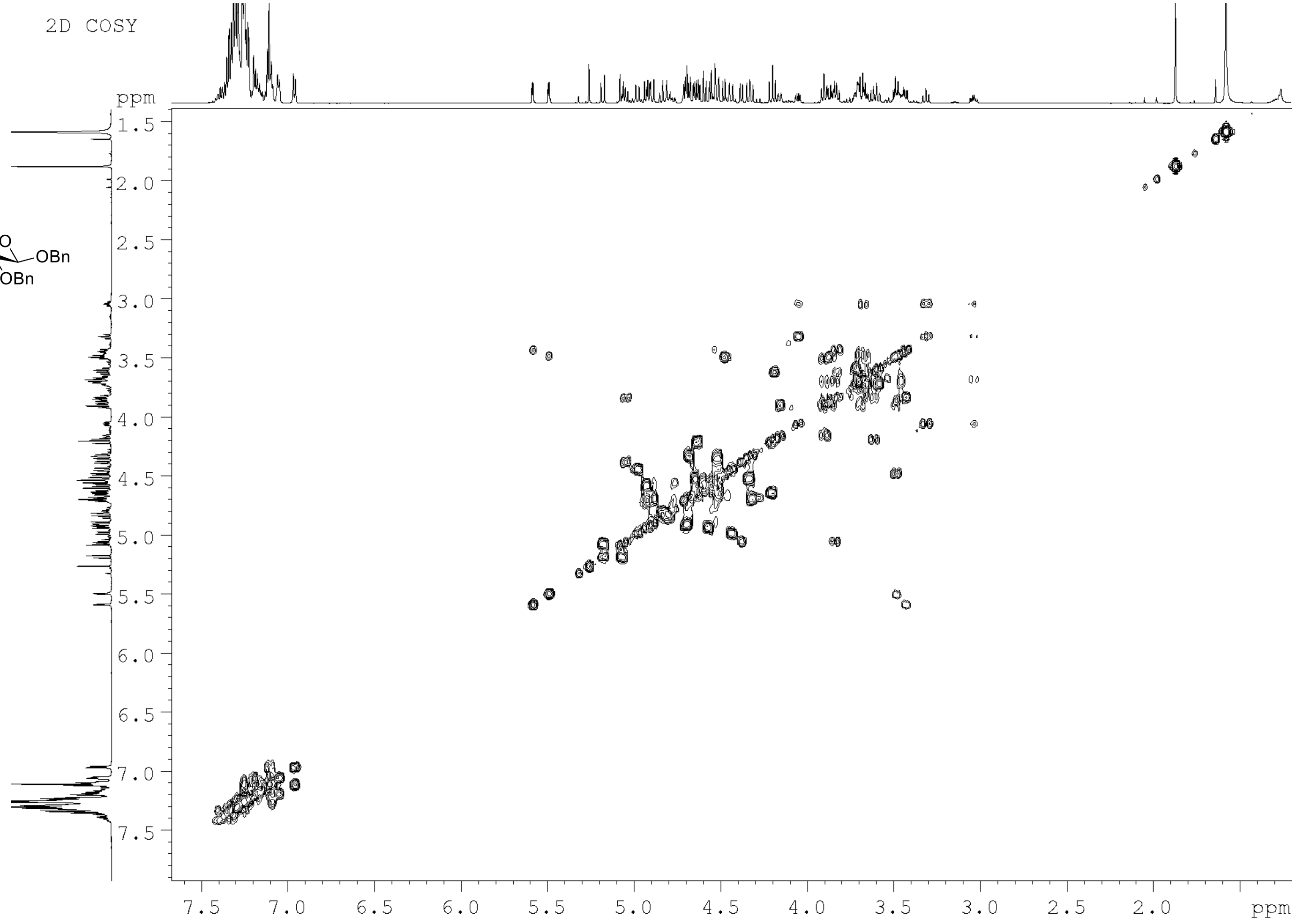

Figure S39. 2D COSY NMR Spectrum of compound **16**

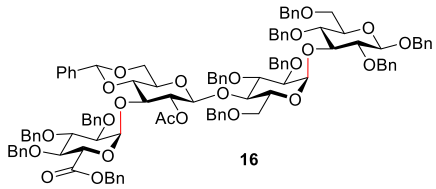

16

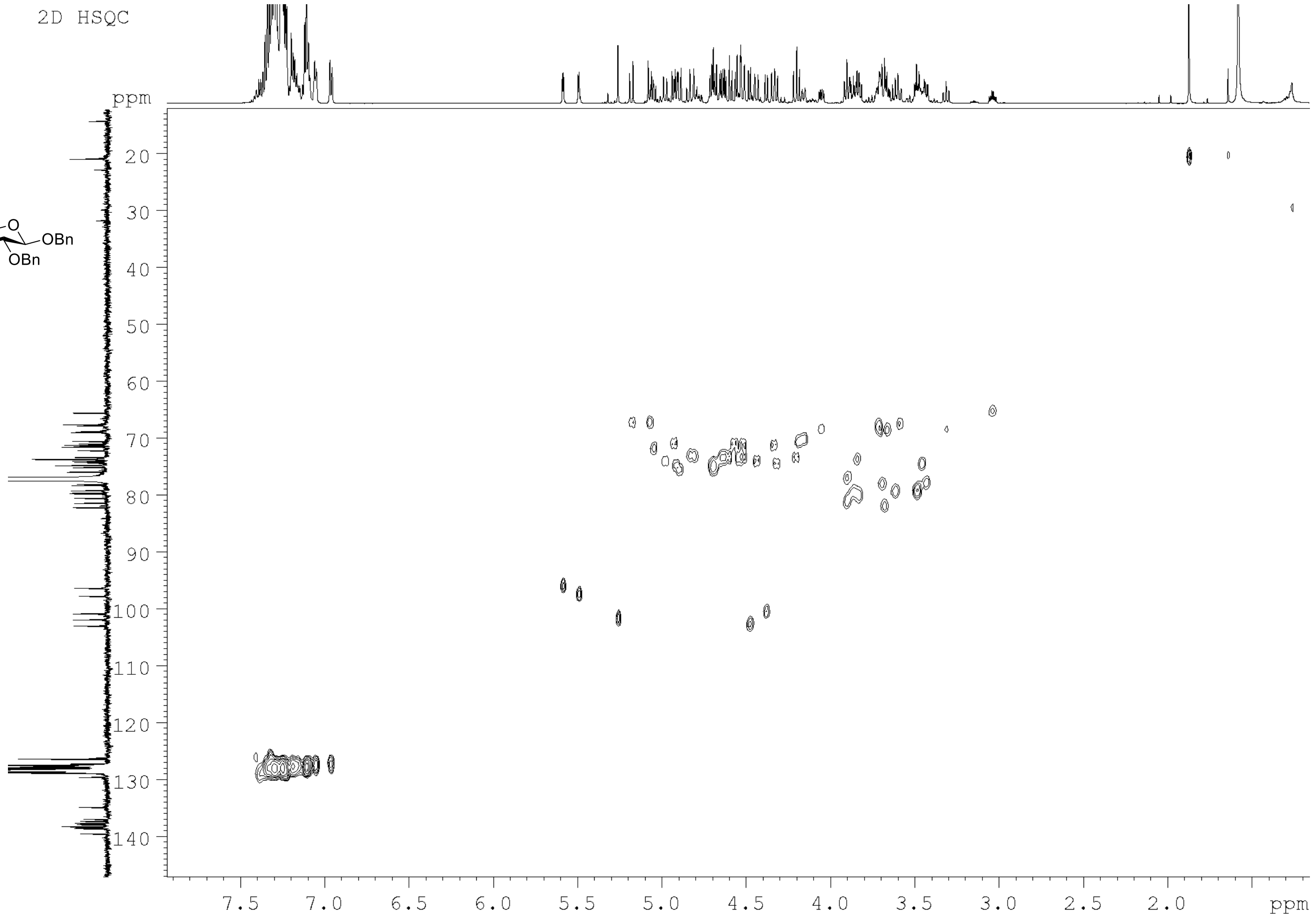

Figure S40. 2D HSQC NMR Spectrum of compound **16**

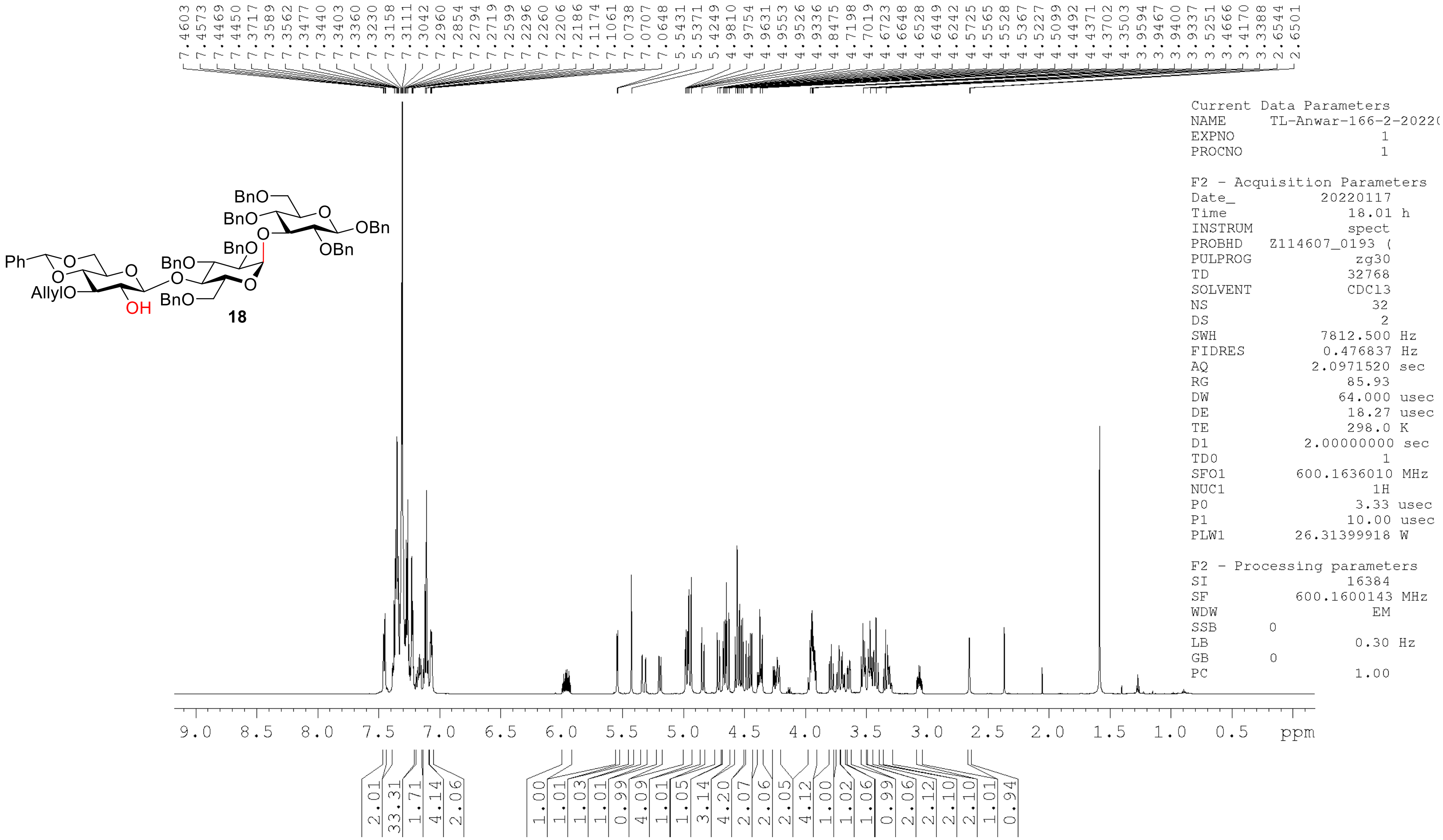

Figure S41. ^{1}H NMR Spectrum of compound **18**

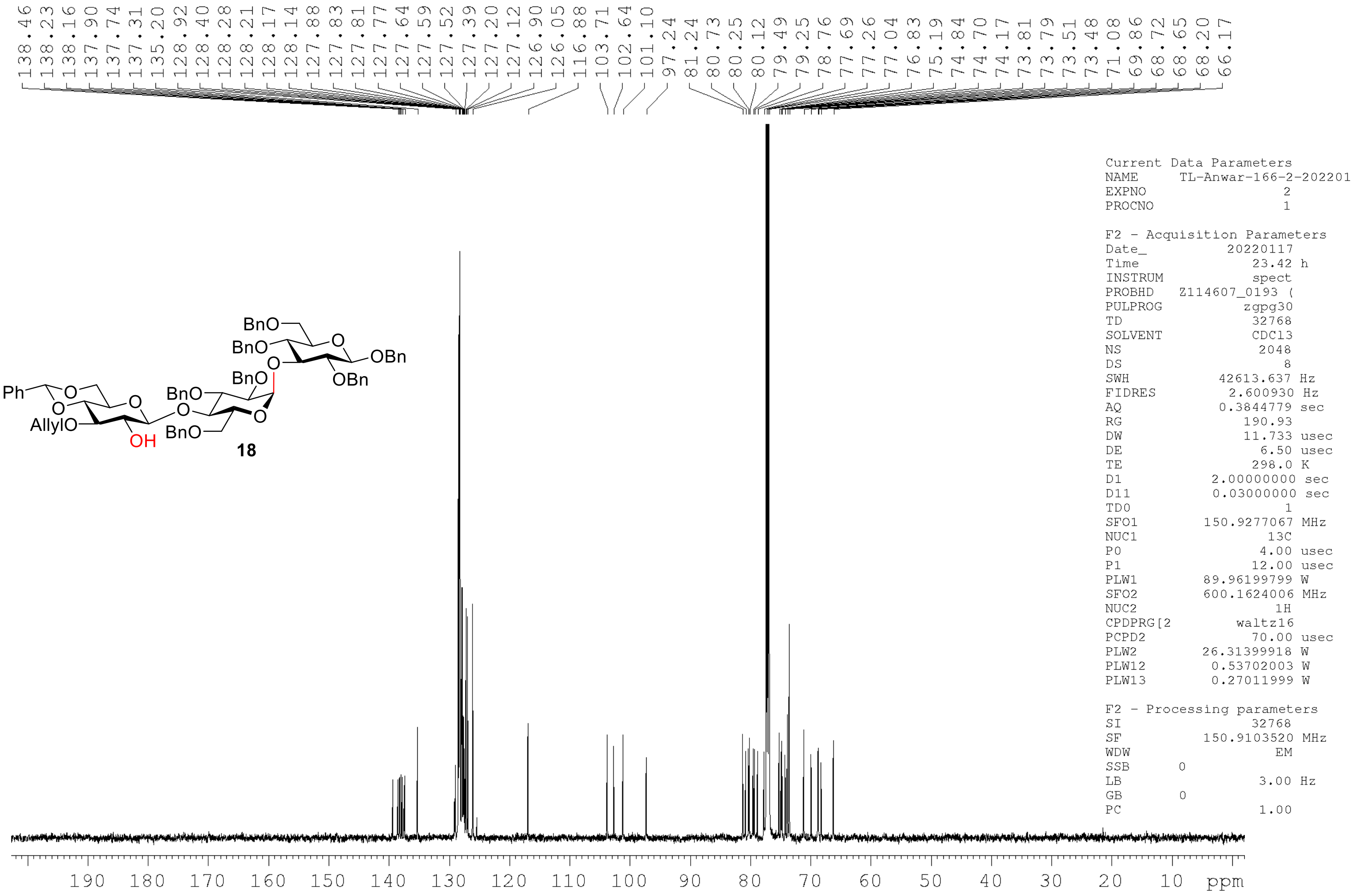

Figure S42. ^{13}C NMR Spectrum of compound **18**

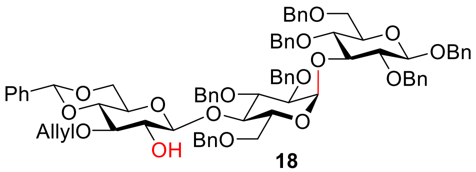

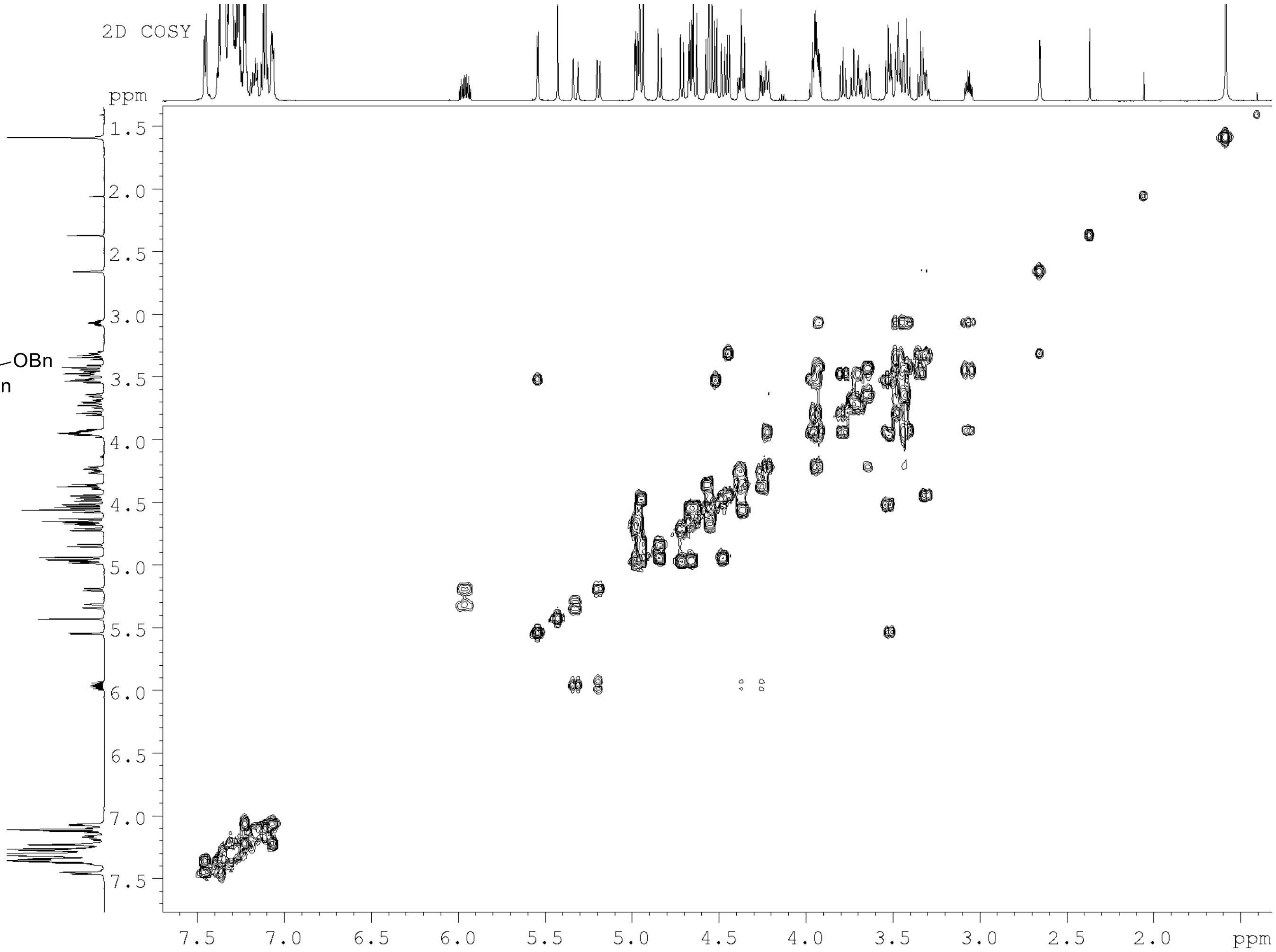

Figure S43. 2D COSY NMR Spectrum of compound **18**

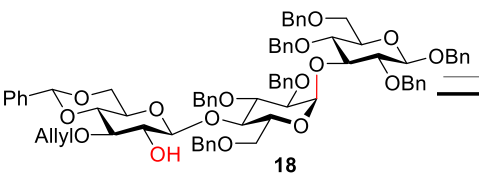

18

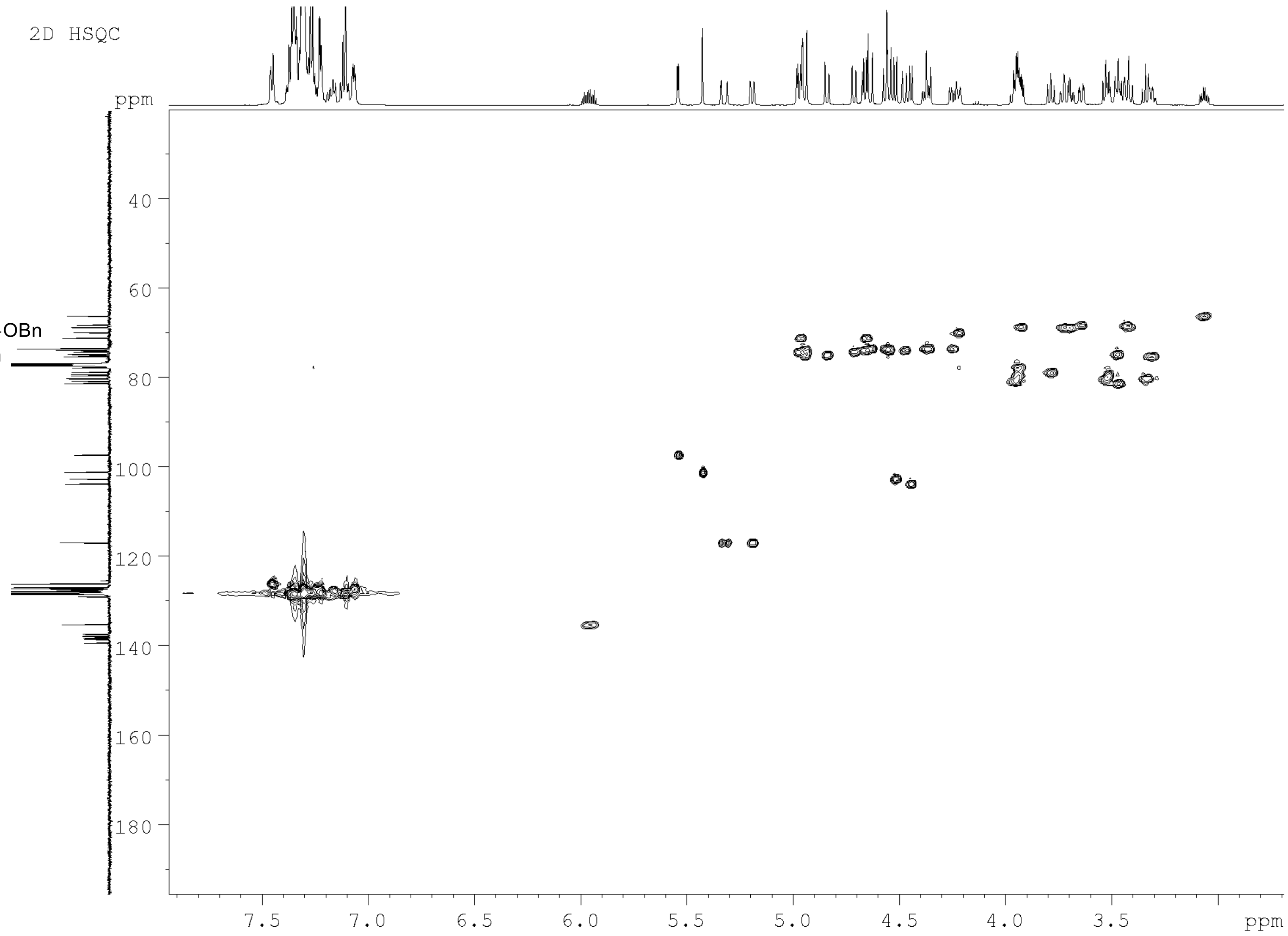

Figure S44. 2D HSQC NMR Spectrum of compound **18**

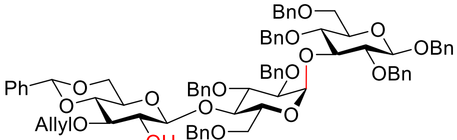

18

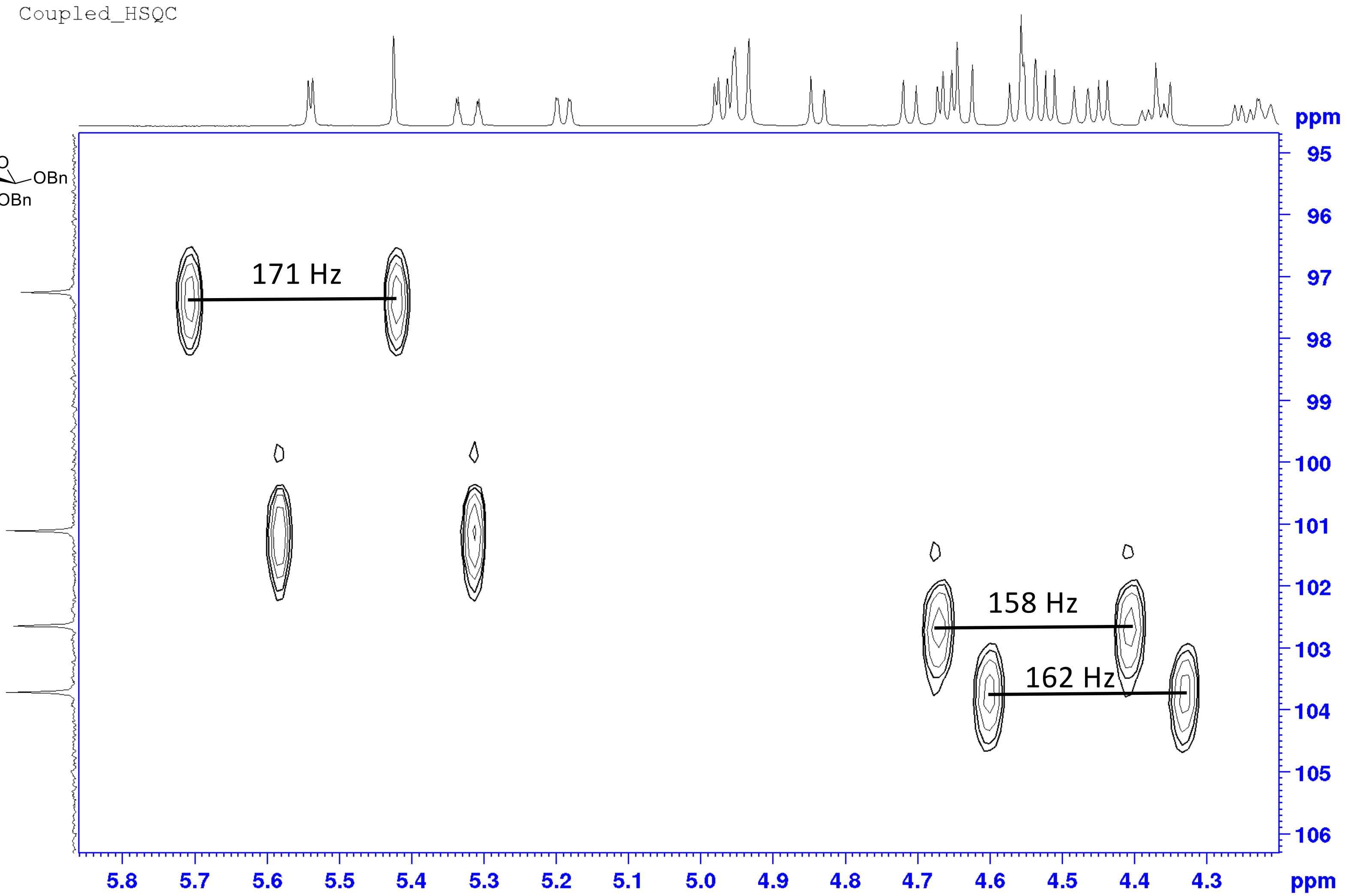

Figure S45. 2D Coupled HSQC NMR Spectrum of compound **18**

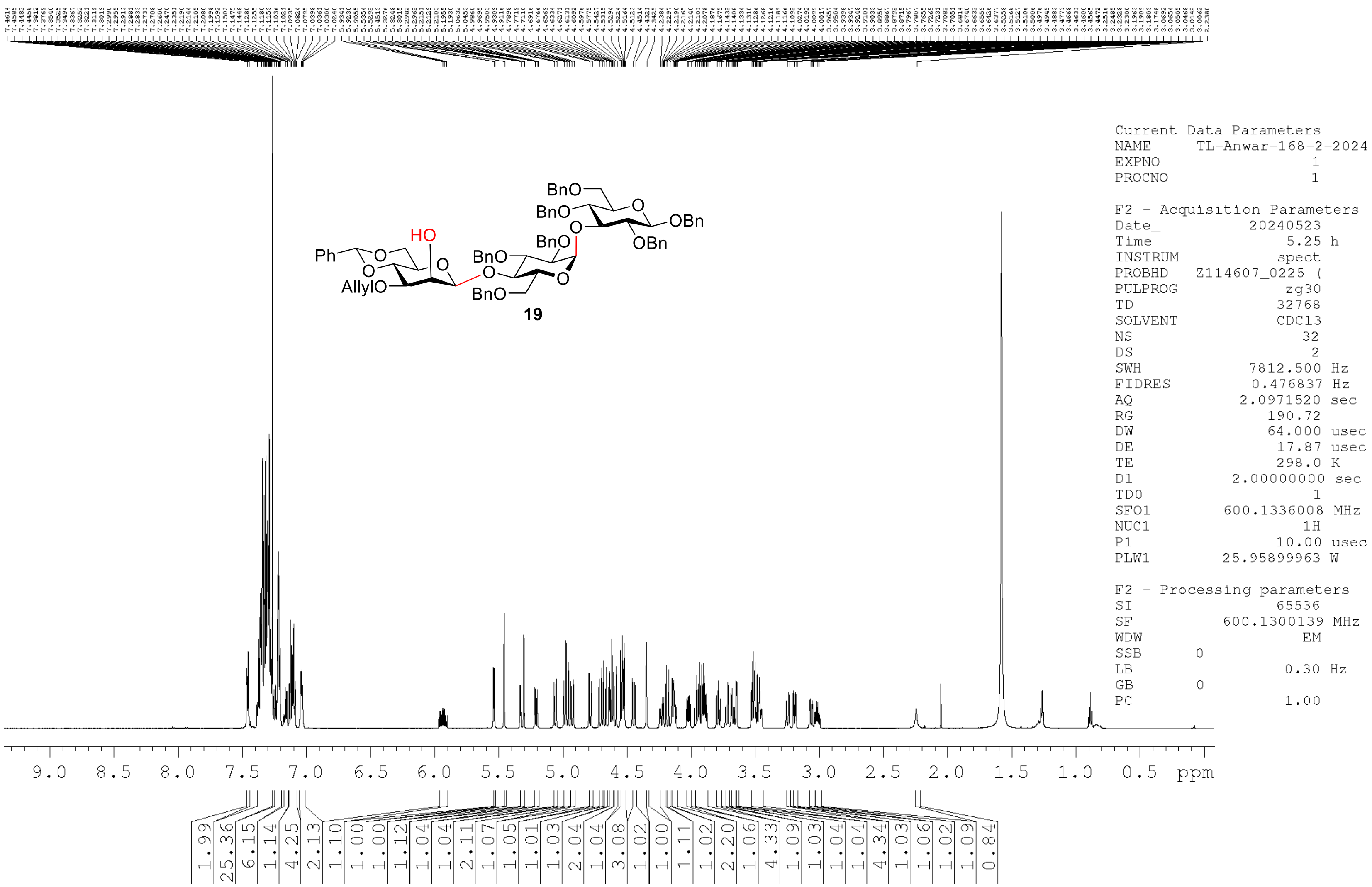

Figure S46. ^{1}H NMR Spectrum of compound **19**

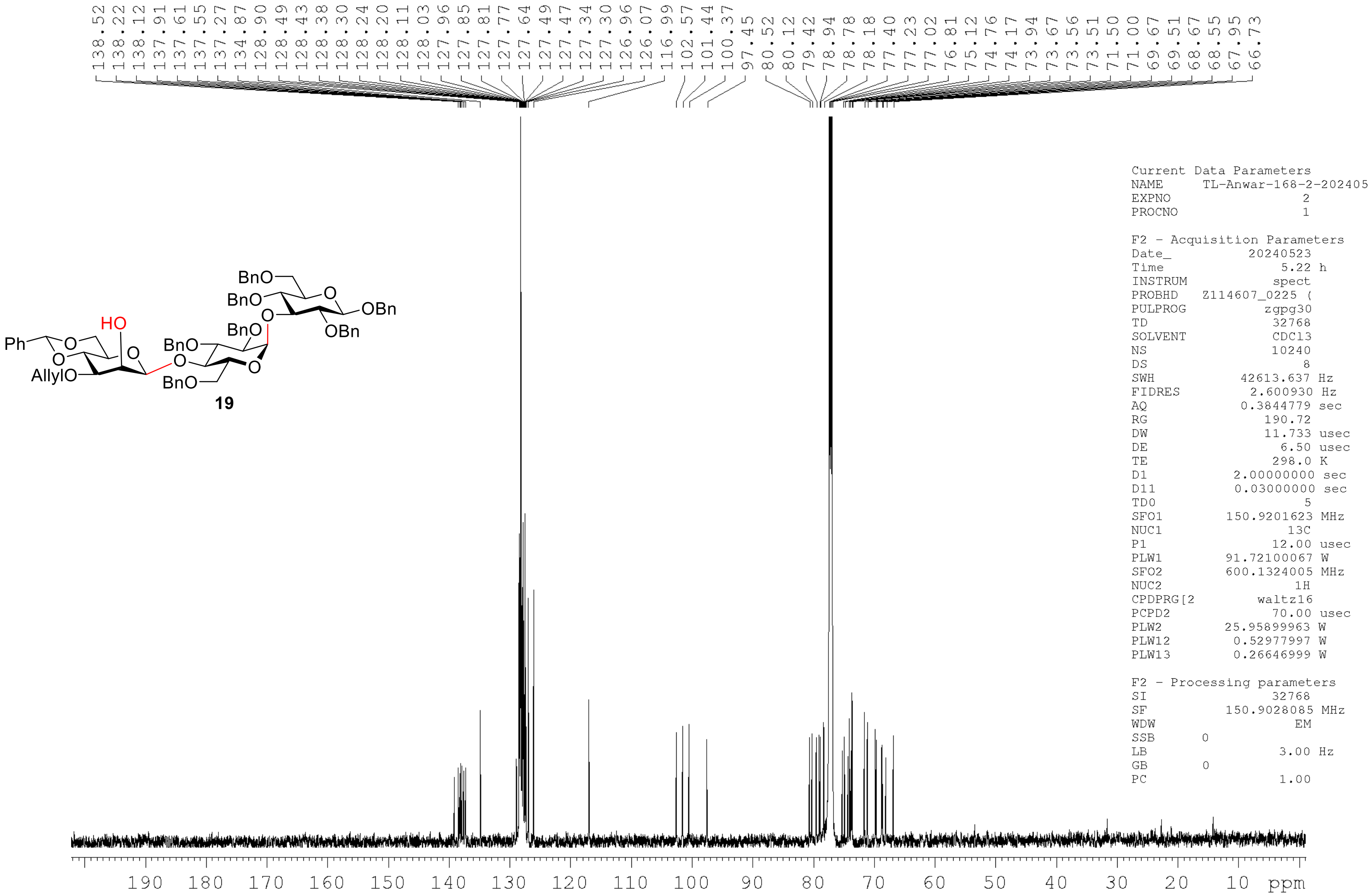

Figure S47. ^{13}C NMR Spectrum of compound **19**

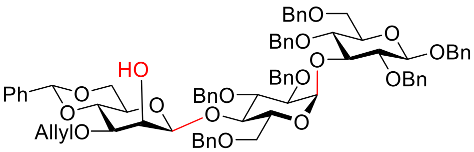

19

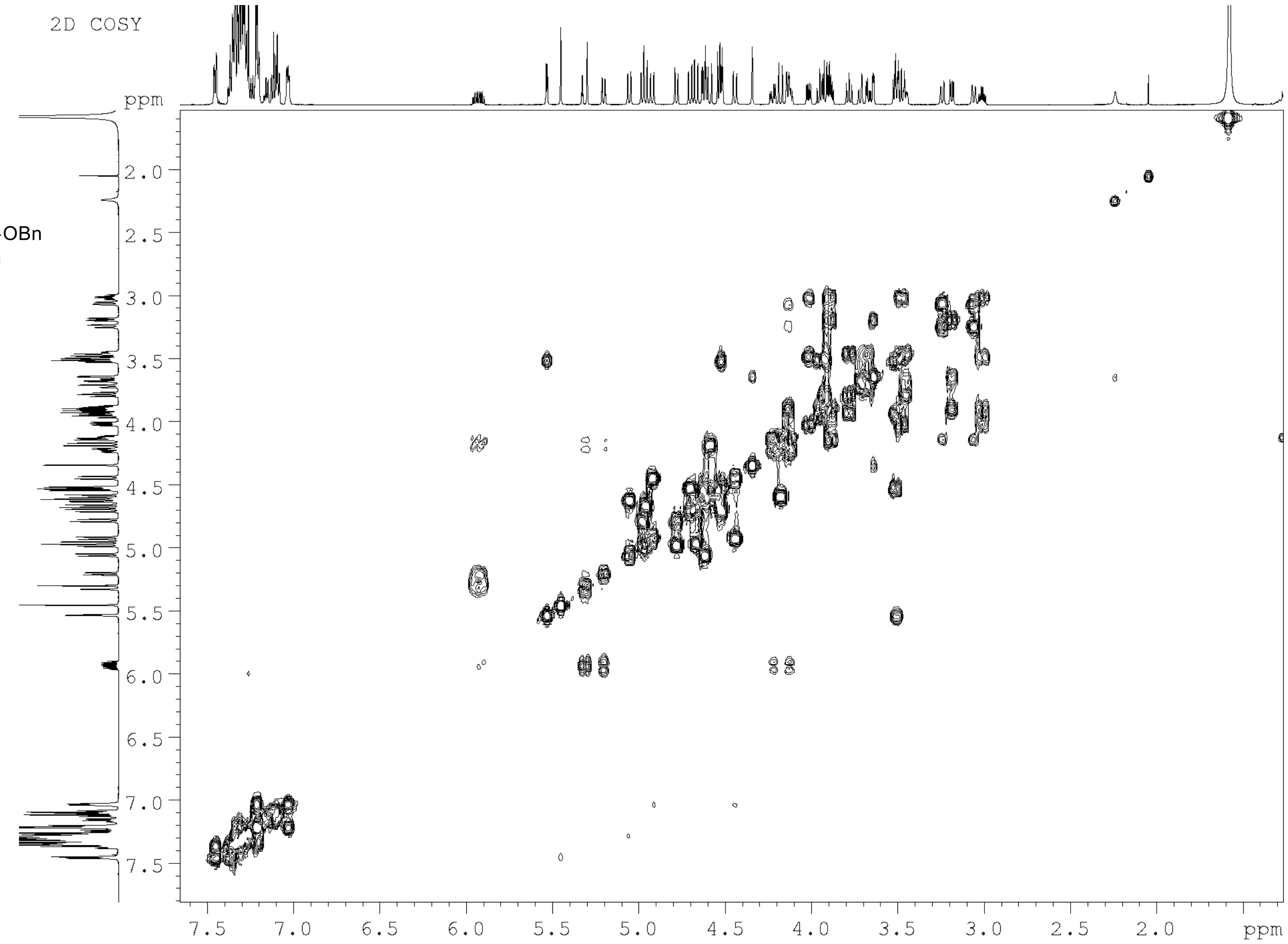

Figure S48. 2D COSY NMR Spectrum of compound **19**

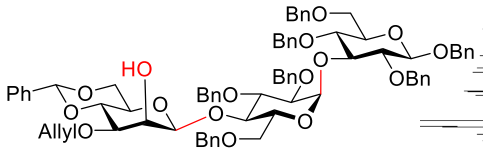

19

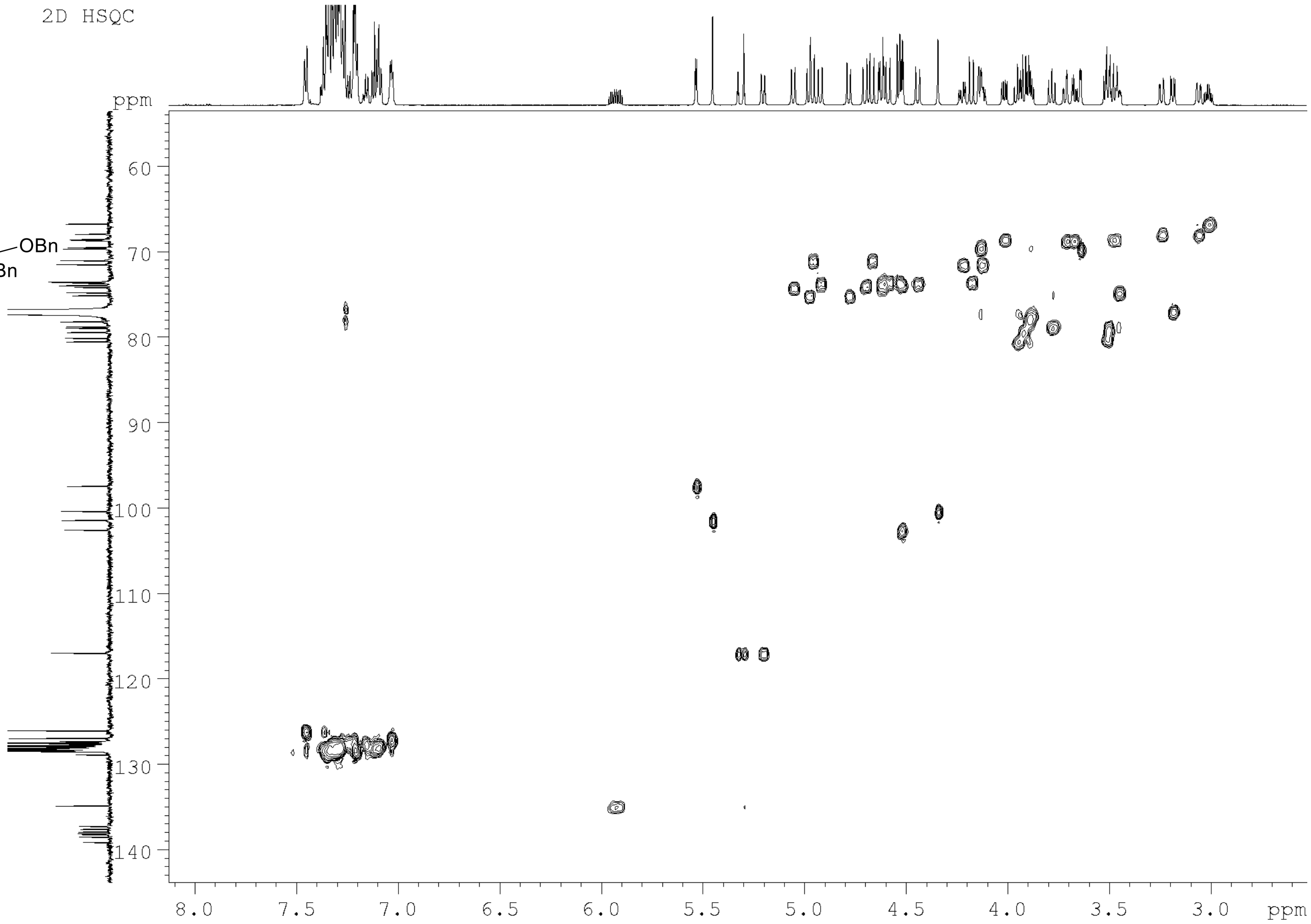

Figure S49. 2D HSQC NMR Spectrum of compound **19**

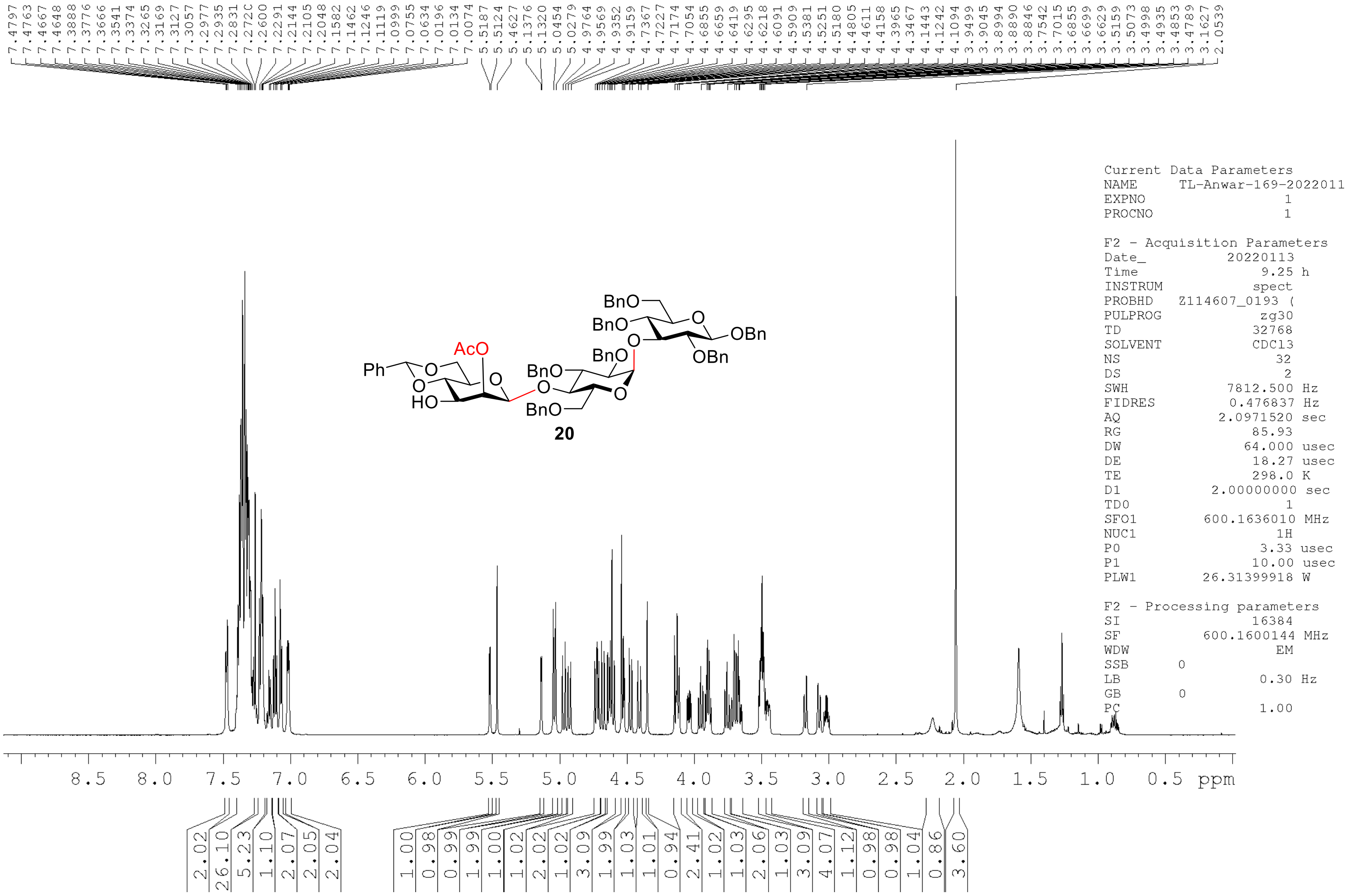

Figure S50. ¹H NMR Spectrum of compound **20**

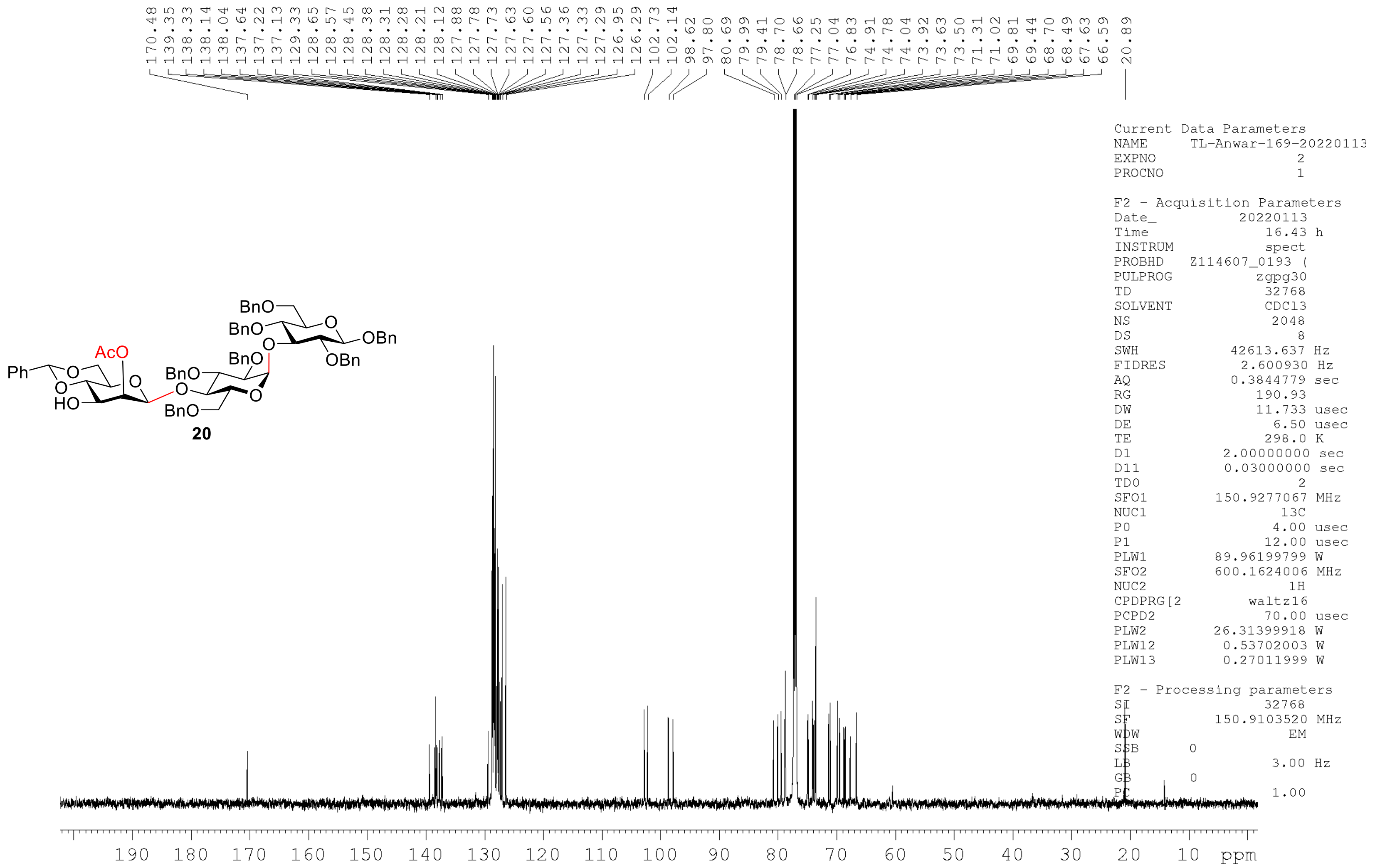

Figure S51. ^{13}C NMR Spectrum of compound **20**

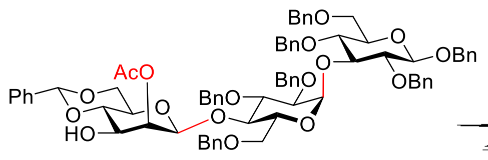

20

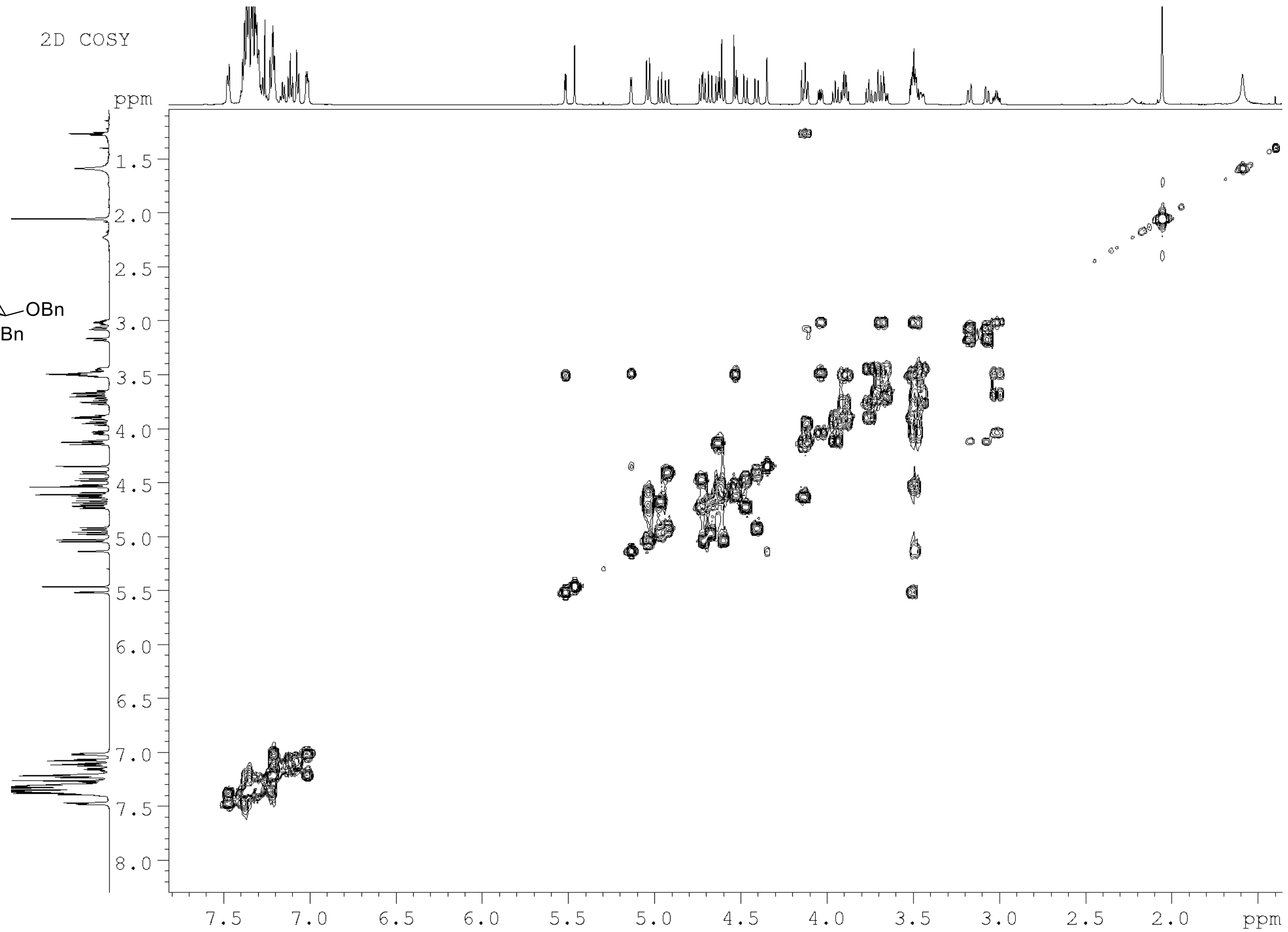

Figure S52. 2D COSY NMR Spectrum of compound **20**

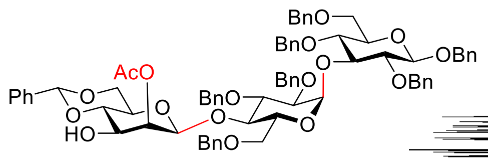

20

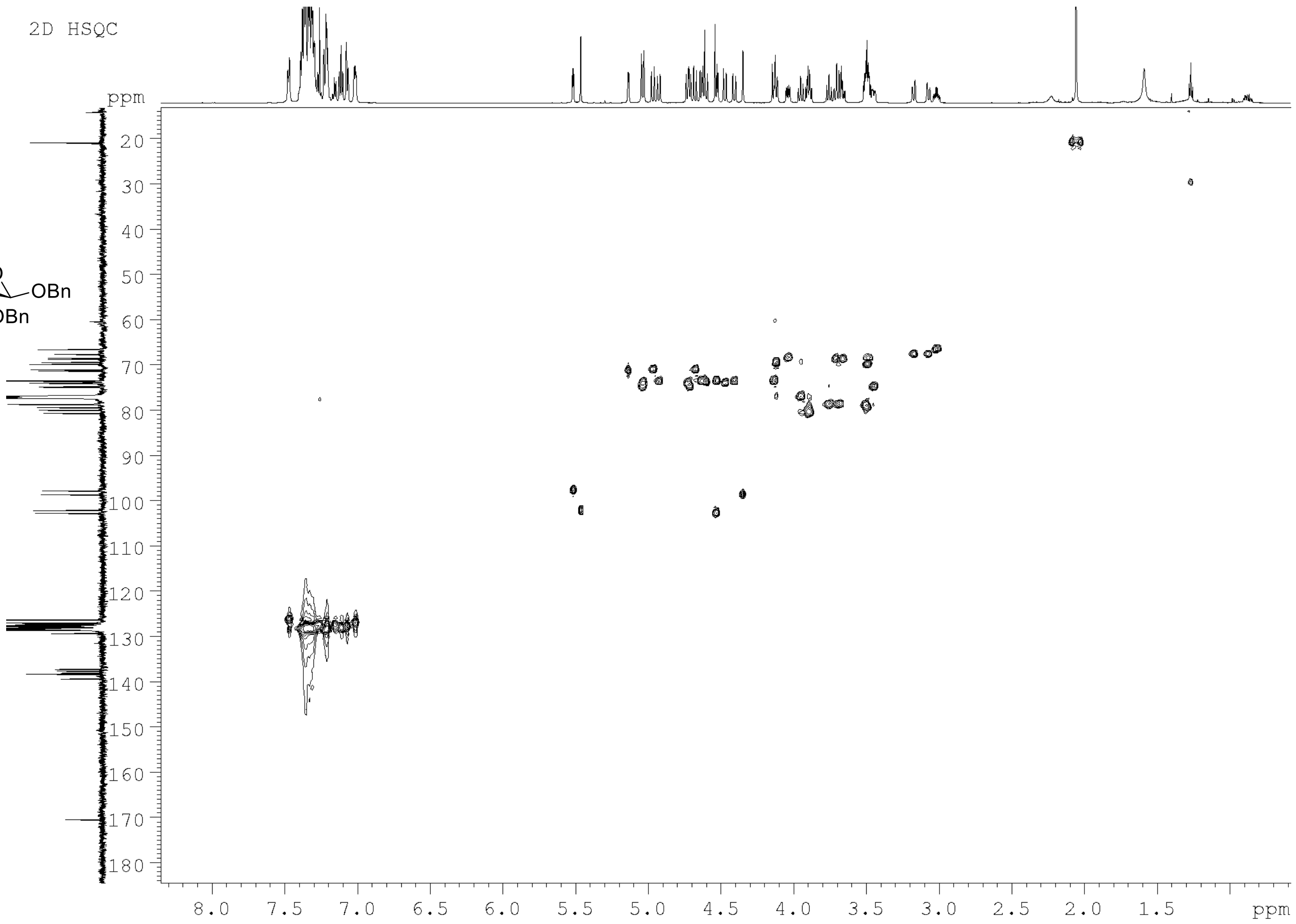

Figure S53. 2D HSQC NMR Spectrum of compound **20**

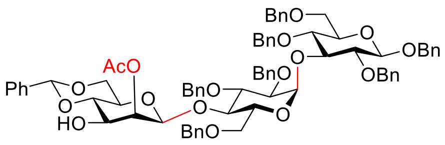

20

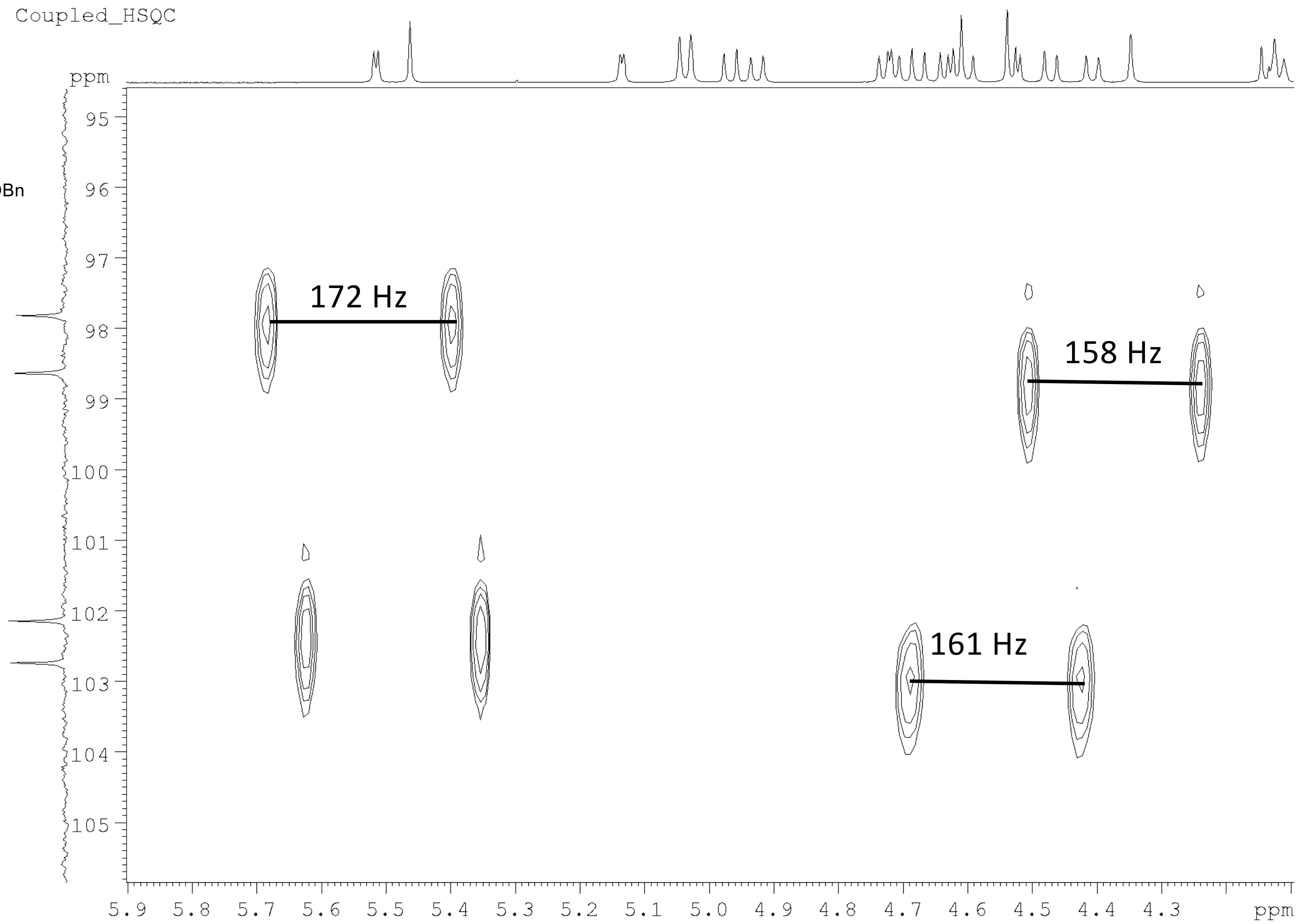

Figure S54. 2D Coupled HSQC NMR Spectrum of compound **20**

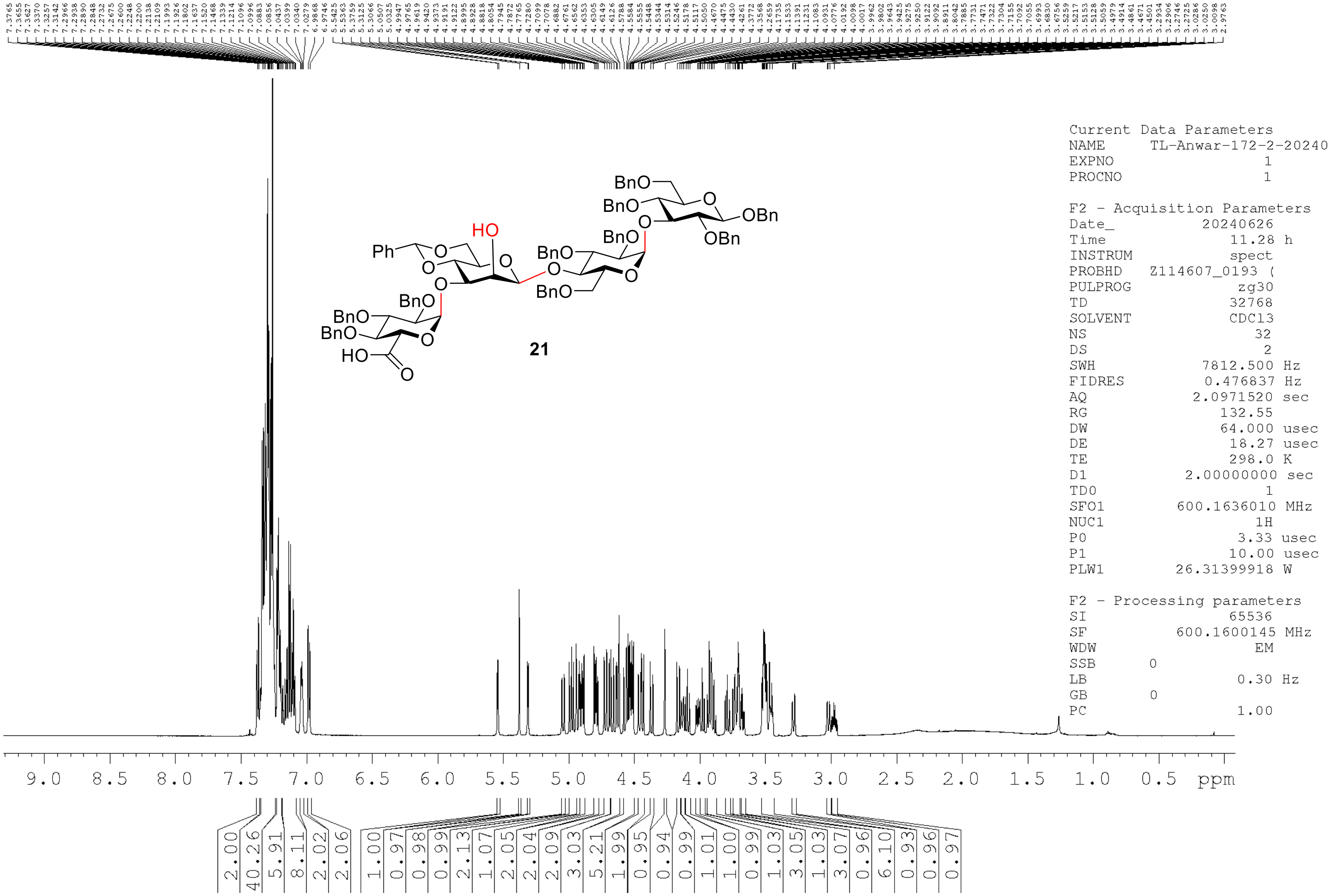

Figure S55. ^{1}H NMR Spectrum of compound **21**

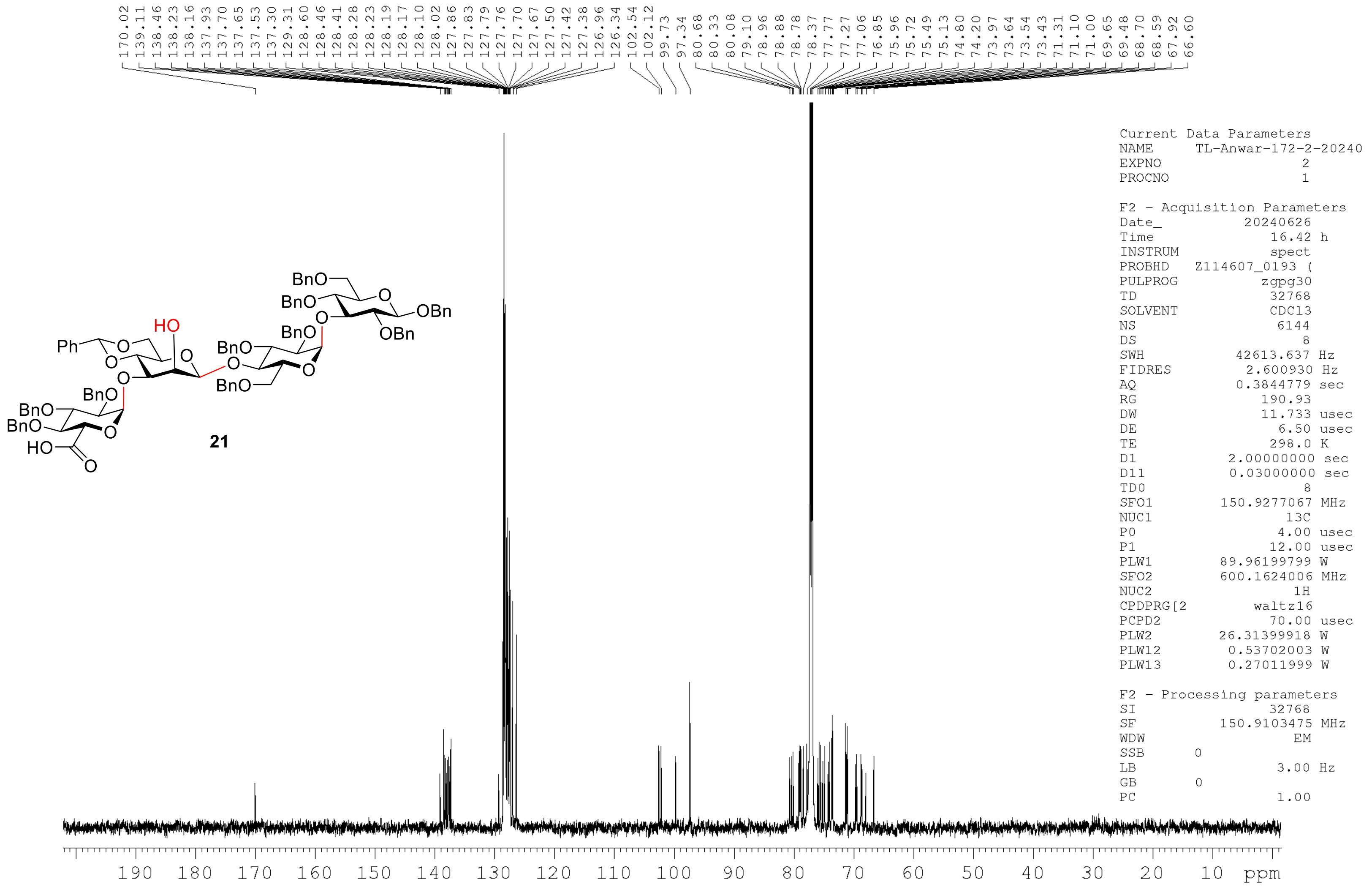

Figure S56. ^{13}C NMR Spectrum of compound **21**

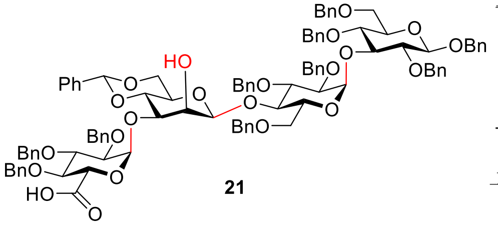

21

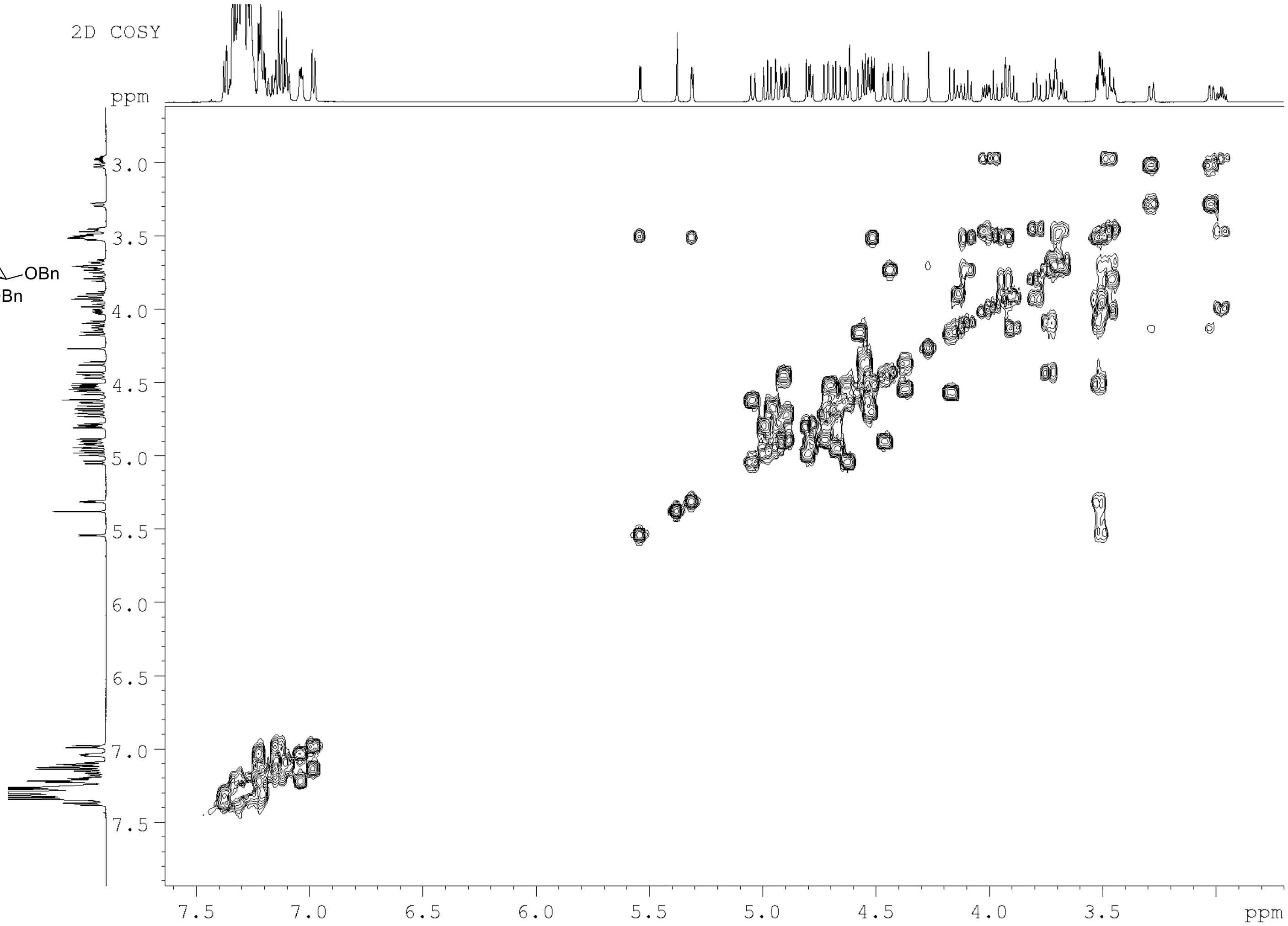

Figure S57. 2D COSY NMR Spectrum of compound **21**

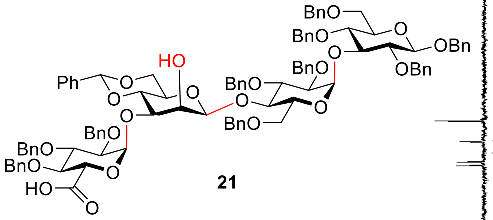

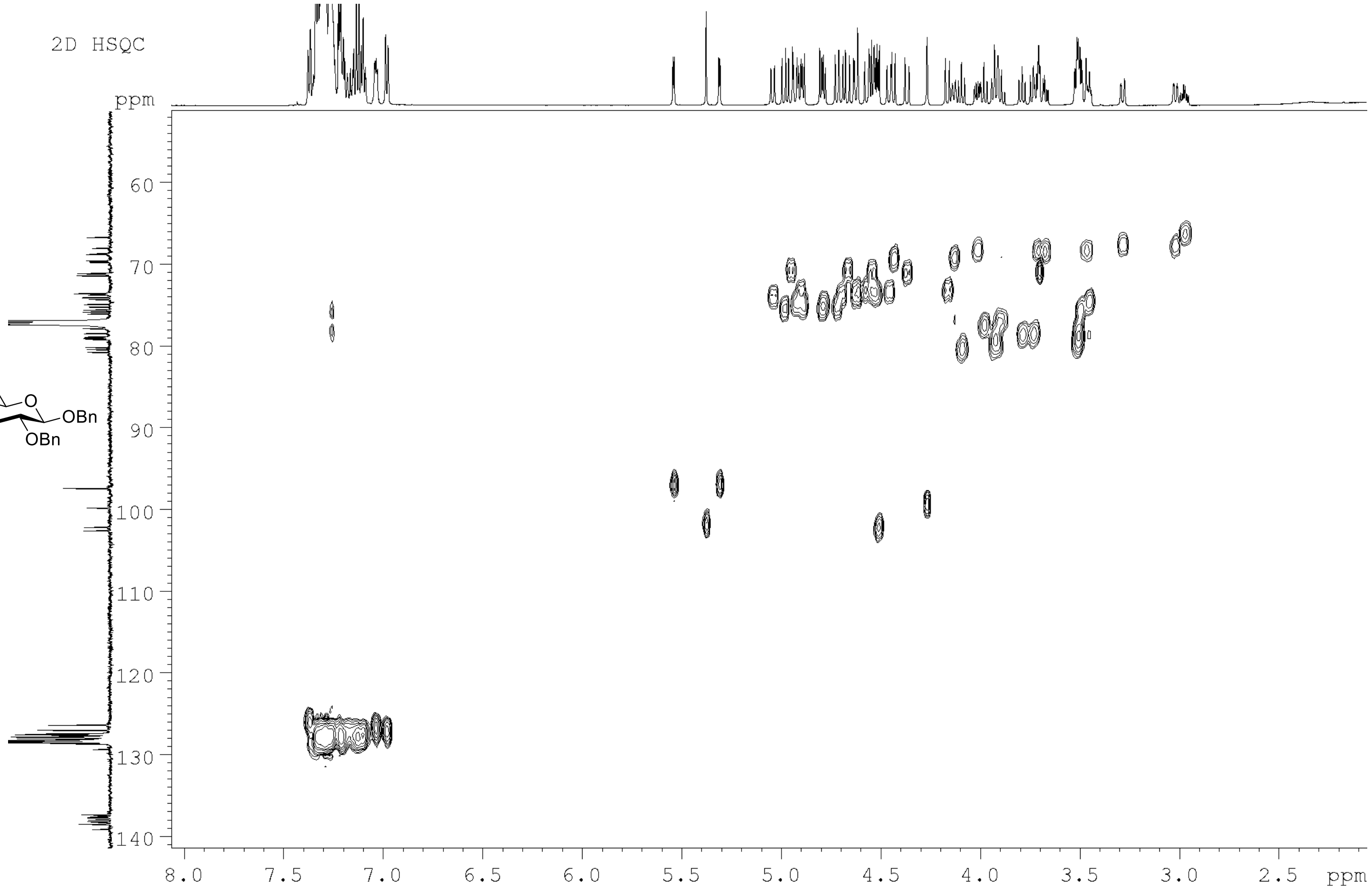

Figure S58. 2D HSQC NMR Spectrum of compound **21**
